# Maternal lipid levels across pregnancy impact the umbilical cord blood lipidome and infant birth weight

**DOI:** 10.1038/s41598-020-71081-z

**Published:** 2020-08-26

**Authors:** Jennifer L. LaBarre, Muraly Puttabyatappa, Peter X. K. Song, Jaclyn M. Goodrich, Ling Zhou, Thekkelnaycke M. Rajendiran, Tanu Soni, Steven E. Domino, Marjorie C. Treadwell, Dana C. Dolinoy, Vasantha Padmanabhan, Charles F. Burant

**Affiliations:** 1 Department of Nutritional Sciences, University of Michigan School of Public Health, Ann Arbor, MI USA; 2 Department of Pediatrics, University of Michigan, Ann Arbor, MI USA; 3 Department of Biostatistics, University of Michigan School of Public Health, Ann Arbor, MI USA; 4 Department of Environmental Health Sciences, University of Michigan School of Public Health, Ann Arbor, MI USA; 5 Center of Statistical Research, Southwestern University of Finance and Economics, Chengdu, Sichuan China; 6 Michigan Regional Comprehensive Metabolomics Resource Core, Ann Arbor, MI USA; 7 Department of Pathology, Michigan Regional Comprehensive Metabolomics Resource Core, Ann Arbor, MI USA; 8 Department of Obstetrics and Gynecology, University of Michigan Medical School, Ann Arbor, MI USA; 9 Department of Internal Medicine, University of Michigan Medical School, Ann Arbor, MI USA

**Keywords:** Lipidomics, Reproductive biology

## Abstract

Major alterations in metabolism occur during pregnancy enabling the mother to provide adequate nutrients to support infant development, affecting birth weight (BW) and potentially long-term risk of obesity and cardiometabolic disease. We classified dynamic changes in the maternal lipidome during pregnancy and identified lipids associated with Fenton BW z-score and the umbilical cord blood (CB) lipidome. Lipidomics was performed on first trimester maternal plasma (M1), delivery maternal plasma (M3), and CB plasma in 106 mother-infant dyads. Shifts in the maternal and CB lipidome were consistent with the selective transport of long-chain polyunsaturated fatty acids (PUFA) as well as lysophosphatidylcholine (LysoPC) and lysophosphatidylethanolamine (LysoPE) species into CB. Partial correlation networks demonstrated fluctuations in correlations between lipid groups at M1, M3, and CB, signifying differences in lipid metabolism. Using linear models, LysoPC and LysoPE groups in CB were positively associated with BW. M1 PUFA containing triglycerides (TG) and phospholipids were correlated with CB LysoPC and LysoPE species and total CB polyunsaturated TGs. These results indicate that early gestational maternal lipid levels influence the CB lipidome and its relationship with BW, suggesting an opportunity to modulate maternal diet and improve long-term offspring cardiometabolic health.

## Introduction

The Developmental Origins of Health and Disease (DOHaD) theory describes how insults during early life can permanently program the fetus/offspring, altering their risk of adult chronic disease^1^. To adapt to the intrauterine environment (i.e. maternal nutrient supply), structural changes and functional modifications occur in fetal organs and tissues to ensure survival of the newborn, indicative of their developmental plasticity^2^. Barker and Osmond developed this hypothesis from observations in England and Wales, confirmed by studies from the Dutch Famine, a well-documented famine in the Netherlands during World War II^3^. Within this cohort, nutrient restriction in utero in the first trimester was associated with increased risk of obesity, dyslipidemia and cardiovascular disease, while restriction in the third trimester was associated with decreased risk of metabolic disease independent of birth weight (BW)^4^, affirming existence of distinct susceptibility windows for producing differing outcomes. Infant BW is the most common health outcome studied to determine if the maternal intrauterine environment influences the development of the fetus. Both low and high BW have been associated with an increased risk of obesity and cardiometabolic diseases, including type 2 diabetes (T2D)^5,6^.

In recent years, profiling of small molecular weight compounds in a biological sample by metabolomics has been used to obtain an objective measurement of the metabolic environment to which the developing fetus is exposed. Metabolite levels are influenced by dietary intake^7^ and can both reflect and influence changes in metabolism^8^. The application of metabolomics to developmental studies can identify changes in maternal nutrient availability across pregnancy^9–12^. Using a targeted metabolomics approach, we previously reported an increase in plasma long-chain fatty acids and corresponding long-chain acylcarnitines (AC) in maternal plasma from first trimester to term^11^. These results reflect the increase in lipolysis during late-gestation to fuel rapid fetal growth. Using a larger cohort and a comprehensive lipidomics platform, we aim to expand our previous findings and detail changes in phospholipids, ceramides (CER), cholesteryl esters (CE), and triglycerides (TG) during pregnancy.

Metabolomics analyses have been applied to identify trimester-specific maternal metabolites associated with infant BW and adiposity^13–17^, accounting for maternal characteristics such as pre-pregnancy BMI^14^. Maternal metabolite levels, placental transfer, and interaction with other metabolites, such as inhibition by competition or metabolite-induced changes in placental transport activity, can all affect the relative levels of the umbilical cord blood (CB) metabolome. The CB metabolome is influenced by maternal characteristics such as pre-pregnancy BMI^18,19^.

The metabolome from CB has been correlated with BW finding positive associations with branched chain amino acids^20^, AC^19,21^, and phosphatidylcholines (PC)^21,22^ along with inverse associations with TGs^19^. Other studies have found that CB lysophosphatidylcholine (LysoPC) metabolites with varying chain length and saturation are positively associated with newborn BW^22–24^, as well as with weight at 6 months of age^22^. However, what remains unclear is how the maternal lipidome influences that establishment of CB lipids related to BW. In this study we profiled 573 lipid species in 106 mother-infant dyads from maternal first trimester (M1) and delivery blood (M3) as well as umbilical CB.

## Results

### Subject demographics

**Figure 1:**
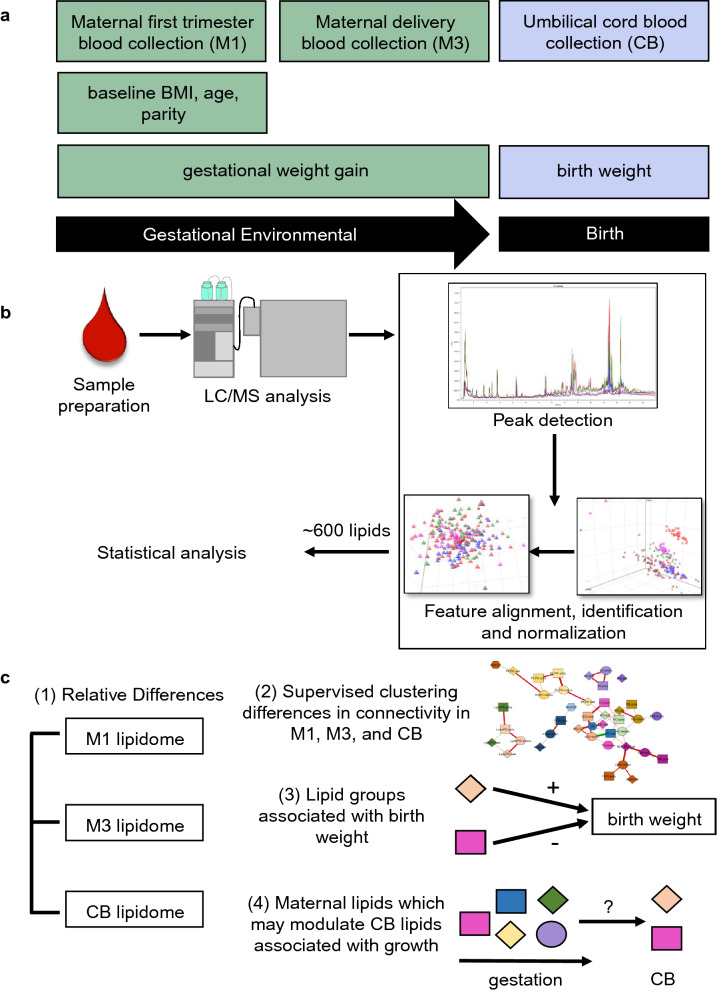
Study design and lipidomics analysis strategy. (**a**) Women were recruited into the Michigan Mother-Infant Pairs Study at their first prenatal appointment between 8 and 14 weeks pregnancy (M1). Venous blood samples were collected at this visit, along with weight, height, and demographic information. At delivery, maternal venous blood samples (M3) and cord blood samples via venipuncture from the umbilical cord (CB) were collected, as well as birth weight. (**b**) Untargeted shotgun lipidomics was conducted on maternal first trimester, delivery, and cord blood plasma. After peak detection and data normalization, 573 lipids of multiple classes were identified. (**c**) Statistical analysis for this manuscript classified (1) relative differences in the lipidome across pregnancy (paired t-test), (2) differences in the connections of lipid groups between M1, M3, and CB (debiased sparse partial correlations), (3) lipid groups associated to birth weight (linear regression), and (4) M1 and M3 lipids that may modulate CB lipids significantly related to birth weight (Pearson’s correlations).

Women were recruited during the first trimester and followed through delivery (Fig. 1a). Average maternal age at their first trimester visit was 32.1 ± 3.6 years and the majority of women had a lean baseline BMI (62%) (Table 1) and were white, non-Hispanic (89%). On average, women gained 13.2 ± 5.2 kg between the baseline visit and term and 72% of infants were born vaginally. Offspring analysis was stratified by males (n = 51) and females (n = 55). We observed no significant difference in Fenton BW percentile between males and females.

**Table 1:** Characteristics of the study populations among 106 MMIP participants, stratified by sex.

Categorical variables	All	Males	Females	p value^A^
	n (%)	n (%)	n (%)	
**Parity**				
0	33 (31%)	16 (31%)	17 (31%)	0.988
1	45 (42%)	21 (41%)	24 (44%)	
2	21 (20%)	11 (22%)	10 (18%)	
≥ 3	7 (7%)	3 (6%)	4 (7%)	
**Baseline BMI (kg/m**^**2**^**) (n = 103)**				
18.5–24.9	64 (62%)	30 (61%)	34 (63%)	0.504
25.0–29.9	18 (17%)	11 (22%)	7 (13%)	
30.0–34.9	12 (12%)	4 (8%)	8 (15%)	
≥ 35.0	9 (9%)	4 (8%)	5 (9%)	
**Delivery mode**				
Vaginal	76 (72%)	35 (69%)	41 (75%)	0.499
Caesarean Section	30 (28%)	16 (31%)	14 (25%)	

Continuous variables	All	Males	Females	p value^B^
	mean ± SD (n)	mean ± SD (n)	mean ± SD (n)	
**Maternal baseline characteristics**				
Age (years)	32.1 ± 3.6	32.4 ± 4.1	31.7 ± 3.2	0.328
Weight (kg)	71.1 ± 17.6	69.4 ± 13.9	72.6 ± 20.4	0.347
Height (cm)	165 ± 7 (103)	165 ± 6 (49)	165 ± 7 (54)	0.858
BMI (kg/m^2^)	25.8 ± 6.0 (103)	25.4 ± 5.3 (49)	26.2 ± 6.6 (54)	0.519
**Maternal and newborn delivery characteristics**				
Gestation age (days)	278 ± 7	278 ± 8	278 ± 6	0.981
GWG (kg)	13.2 ± 5.2	13.9 ± 4.5	12.6 ± 5.8	0.202
Birth weight (g)	3,510 ± 436 (105)	3,638 ± 467	3,390 ± 370 (54)	0.003
Fenton BW z score	0.09 ± 0.87 (105)	0.20 ± 0.95	− 0.01 ± 0.78 (54)	0.208
Fenton BW percentile	53.1 ± 26.1 (105)	56.0 ± 28.2	50.4 ± 23.8 (54)	0.273
Head circumference (cm)	35 ± 1 (101)	35.2 ± 1.2 (48)	34.7 ± 1.3 (53)	0.048
Fenton HC z score	0.10 ± 0.86 (101)	0.16 ± 0.72 (48)	0.04 ± 0.96 (53)	0.485
Fenton HC percentile	52.5 ± 25.5 (101)	54.9 ± 22.9 (48)	50.4 ± 27.6 (53)	0.371

Values are n (%) or mean ± SD. Stratified by males (n = 51) and females (n = 55). ^A^Represents Pearson's chi-square test for categorical variables. ^B^Represents unpaired t test for continuous variables. If sample size differs, mean ± SD (n).

Maternal BMI was inversely associated with gestational weight gain (GWG) (beta = − 0.360, r^2^ = 0.18, p < 0.001), indicating that leaner women gained more weight during pregnancy (Supplementary Figure S1a). In contrast, maternal BMI was positively associated with Fenton BW percentile (beta = 0.011, r^2^ = 0.06, p = 0.014) (Supplementary Figure S1b); and while not significant, maternal GWG trended towards a positive association with Fenton BW percentile (beta = 0.009, r^2^ = 0.03, p = 0.070) (Supplementary Figure S1c).

### Fluctuations in the lipidome between M1, M3, and CB

**Figure 2:**
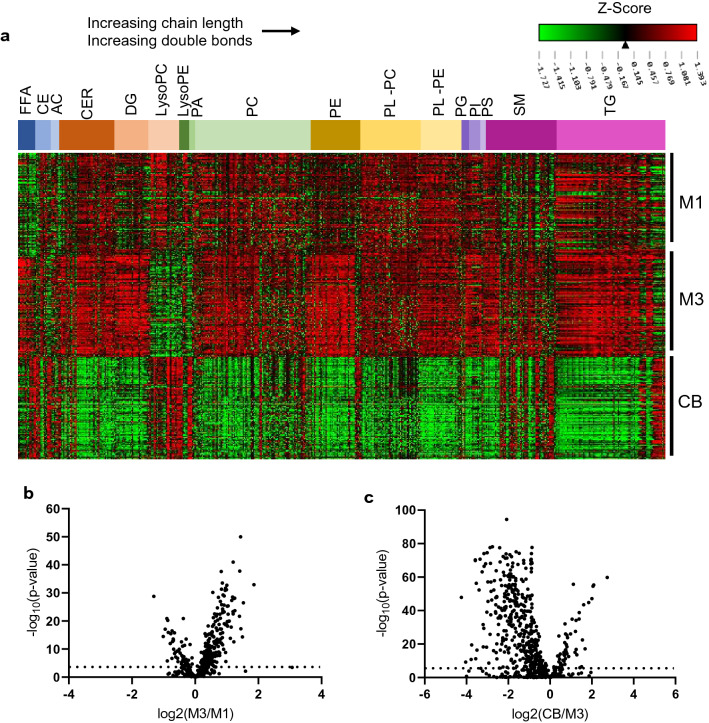
Dynamic changes in maternal and cord blood plasma lipidome during pregnancy. (**a**) Heatmap of standardized peak intensity for individual lipids (mean 0, standard deviation 1). Lipids are grouped by lipid class with increasing total chain length and double bond from left to right in each lipid class. (**b**) Differences between M1 and M3 lipidome using log2FC. Lipids that are greater in M1 than M3 have a positive log2FC. Of the 573 lipid species analyzed, 35% were significantly increased and 10% were decreased (Bonferroni adjusted, α = 0.05/573). (**c**) Differences between M3 and CB lipidome using log2FC. Of the 573 lipid species analyzed, 10% were significantly increased and 61% were decreased (Bonferroni adjusted, α = 0.05/573). Lipids that are greater in M3 than CB have a positive log2FC.

We next examined the changes in the plasma lipidome across pregnancy (M1–M3) and between maternal delivery plasma and cord blood (M3–CB), the latter as a potential surrogate for placental transfer and fetal exposure. Figure 2a displays a Heatmap of lipids organized by class with individual lipid peak intensities normalized across M1, M3, and CB. Figure 2b,c displays fold changes (FC) of lipids from M1 to M3 and between M3 and CB, respectively (quantified in Supplementary Table S1). Most classes of lipids increased in levels from M1 to M3, consistent with previous observations of generalized increase in lipoproteins and associated lipids with advancing pregnancy^9,25^. A subset of lipid species was reduced in M3 compared to M1 including polyunsaturated CEs and a variety of LysoPC and lysophosphatidylethanolamine (LysoPE) species. Most of the polyunsaturated fatty acid (PUFA) containing lipids that were lower in M3 were significantly increased in CB plasma (Fig. 2a,c, Supplementary Table S1). In addition, several lipid species that are statistically unchanged from M1 to M3 were higher in CB compared to M3, including sphingomyelins (SM) and TG enriched in PUFAs. These results are consistent with the known specific transfer of PUFAs across the placenta to the fetus^26^, perhaps mediated by lysophospholipids (LysoPLs) (see below)^27^.

### Partial correlation network of lipid groups within each time-point

**Figure 3:**
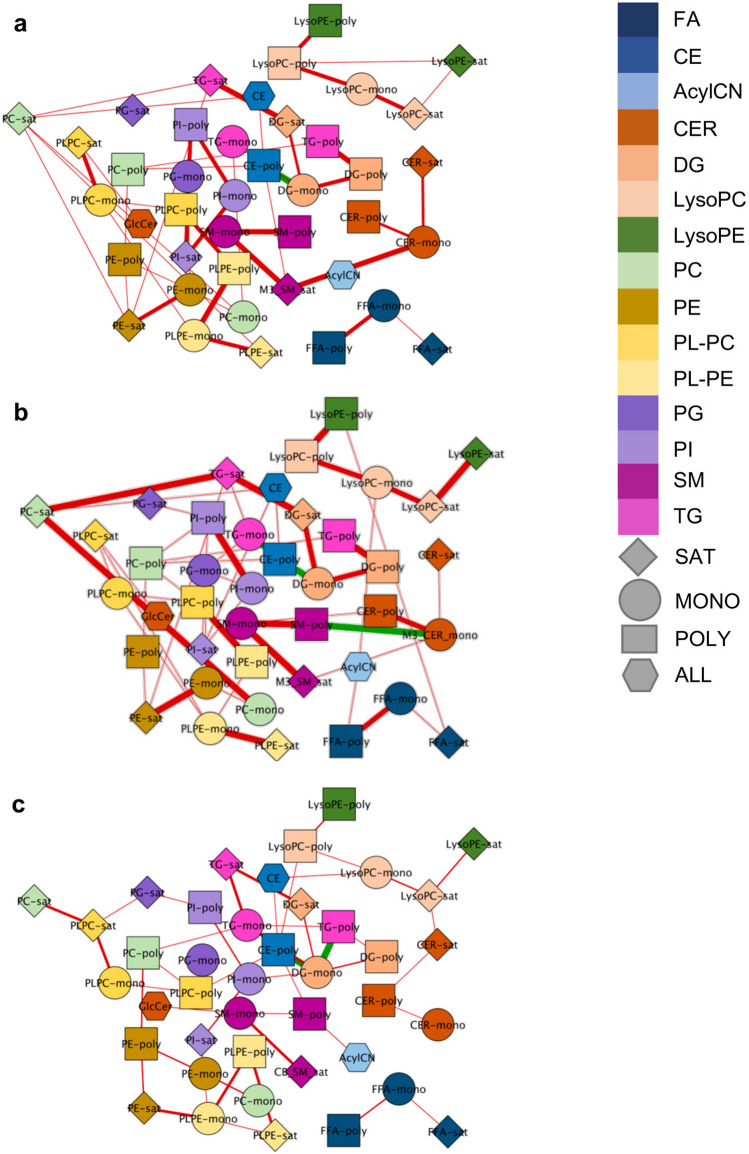
Correlation between lipid groups differs between maternal and cord blood samples. The relationship between lipid groups was estimated using debiased sparse partial correlations within (**a**) M1 (45 edges), (**b**) M3 (55 edges), and (**c**) CB (47 edges). Colors of nodes depict class of lipid. Shapes of nodes depict number of double bonds. Positive correlations are depicted using red edges. Inverse correlations are depicted using green edges. Weight of the edges signifies a more significant correlation. Edges represented have an adjusted p-value < 0.1.

Given the specific patterns within lipid classes containing PUFAs, lipid clusters were created by grouping lipids based on a priori knowledge of lipid class and the number of double bonds in each lipid species, resulting in 41 groups at each time point (Supplementary Table S2). Using a Debiased Sparse Partial Correlation algorithm^28^, we estimated partial correlation networks for lipid groups within M1, M3, and CB, using an adjusted p-value less than 0.1 as a threshold for including edges in the network (Fig. 3). At each time point, the majority of correlations were positive, signifying the connectivity of lipids across classes. In particular, multiple positive correlations were observed between (1) TGs, diacylglycerols (DG), and phospholipids; (2) CERs and SMs; (3) plasmenyl-phosphatidylcholine (PL-PC) and plasmenyl-phosphatidylethanolamine (PL-PE); and (4) LysoPCs and LysoPEs. Most positive correlations occurred between lipid groups containing lipids with similar number of double bonds. CEs are correlated with a variety of lipid classes including LysoPCs, phospholipids, DGs, SMs, and TGs, suggesting their interaction with a variety of lipid metabolic pathways. Of note, in M1, M3, and CB, CE-poly is inversely associated with DG-mono and, excluding M1, with TG-mono. M3 and CB have more significant correlations with each other than with M1 (Fig. 3), likely due to maternal lipolysis and delivery of fatty acids to the fetal circulation during late gestation resulting in proportional distribution to different lipids species. Additionally, eight partial correlations, all positive, were significant between lipid groups in M1 and M3, including between (1) M1 and M3 PE-poly, (2) M1 and M3 PC-poly, (3) M1 and M3 PLPE-poly, and (4) M1 and M3 SM-poly, suggesting that maternal polyunsaturated fatty acid levels track across pregnancy (Supplementary Figure S2). Partial correlations with an adjusted p-value less than 0.1 are reported in Supplementary Table S3.

### Relationship between maternal characteristics with the maternal and cord blood lipidome

The next objective was to assess the relationship between maternal characteristics, baseline BMI and GWG, and the lipidome at M1, M3, and CB. At each time point, multiple lipid classes were associated with BMI and GWG, adjusting for sex, maternal age, parity, and gestational age. These associations were largely in opposite directions, due to the inverse correlation between BMI and GWG (Supplementary Figure S1a). Maternal BMI was inversely associated with PCs and PL-PEs at both M1 and M3, while GWG was positively associated with LysoPC and LysoPE clusters at both M1 and M3, independent of the number of double bonds in the fatty acid tails, with stronger associations evident in M3 (FDR < 0.1) (Supplementary Figure S4; Supplementary Table S5). On the other hand, maternal BMI was positively associated with SM-mono and SM-poly at both M1 and M3, while GWG showed inverse associations with SM-mono and SM-poly, again at both M1 and M3 (Supplementary Figure S3, Supplementary Table S4). Finally, maternal BMI was positively associated with saturated phosphatidylglycerols (PG) and SM-poly in CB, whereas few associations were observed between GWG and lipids groups in CB.

### Relationship between maternal and cord blood lipidome and birth weight

**Figure 4:**
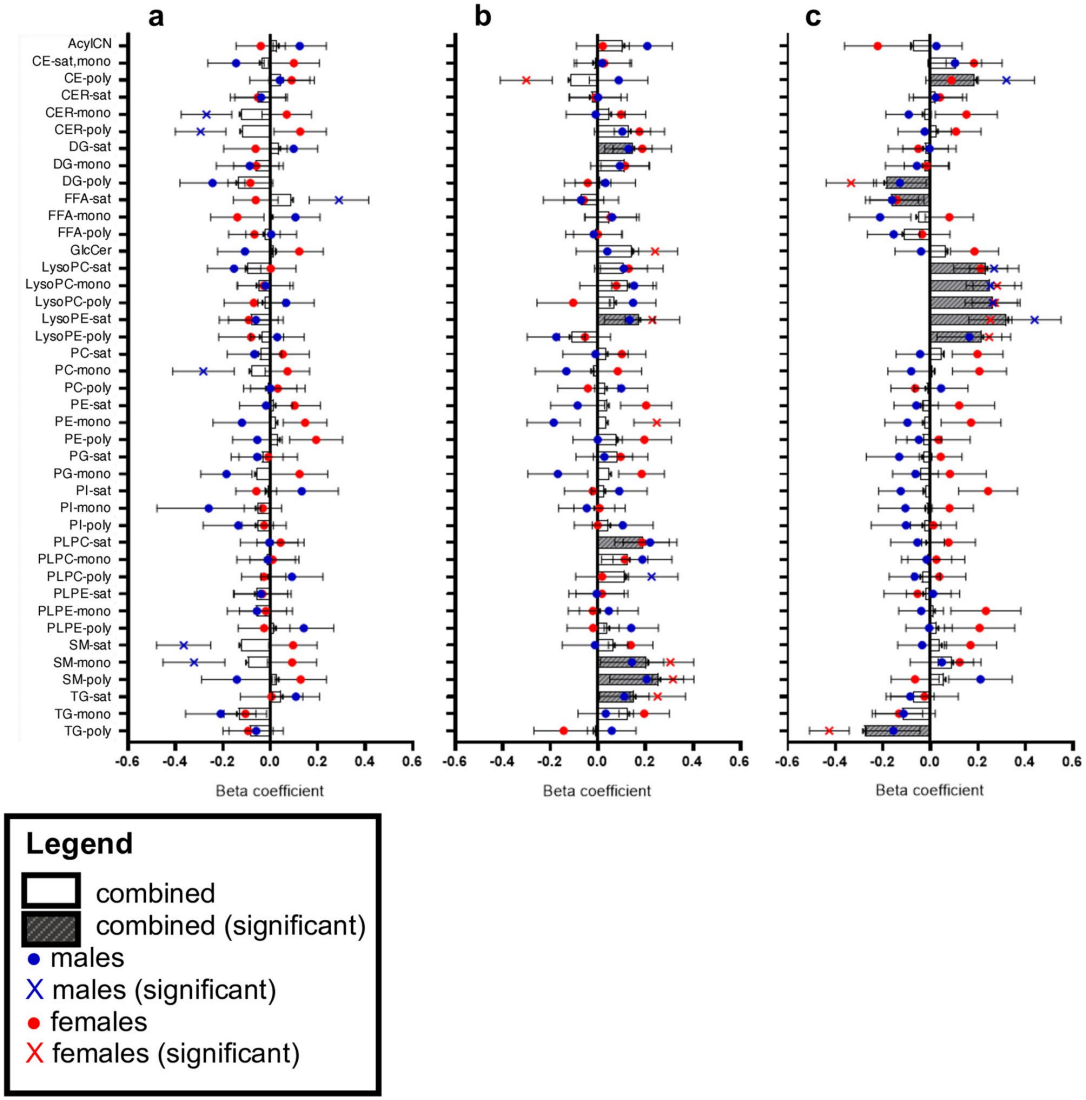
Distinct maternal and cord blood lipid groups are related to birth weight. Regression models estimated the linear relationship between lipid groups from (**a**) maternal first trimester, (**b**) maternal term, and (**c**) cord blood and Fenton BW z score, adjusting for sex, maternal age, parity, gestational age, baseline BMI, and GWG. For the combined model, beta coefficients plotted as gray bars (β ± SE) with significance depicted as gray striped bars (α = 0.05). Sex stratified models were run. For males, beta coefficients are plotted as blue dots (β ± SE) with significance depicted as blue “X” (α = 0.05). For females, beta coefficients are plotted as red dots (β ± SE) with significance depicted as red “X” (α = 0.05).

We identified maternal and CB lipid groups associated with Fenton BW z score (Fig. 4, Supplementary Table S6). In the combined model, the early gestation lipidome (M1) was not associated with infant BW, however, in the sex-stratified models, M1 lipid groups were associated with BW in males (unadjusted p value < 0.05) (Fig. 4a), including positive associations with saturated free fatty acids (FFA) and negative associations with CER-mono, CER-poly, PC-mono, SM-sat, and SM-mono. Within M3, significant positive correlations were observed between BW and DG-sat, LysoPE-sat, PL-PC-sat, SM-mono, SM-poly, and TG-sat (unadjusted p value < 0.05) (Fig. 4b). The direction of these associations was consistent within males and females, however in the sex-stratified models, the relationship reached significance only in females. Of interest, in females, M3 SM-mono and SM-poly were positively associated and CE-poly was inversely associated with BW (FDR < 0.1).

**Figure 5:**
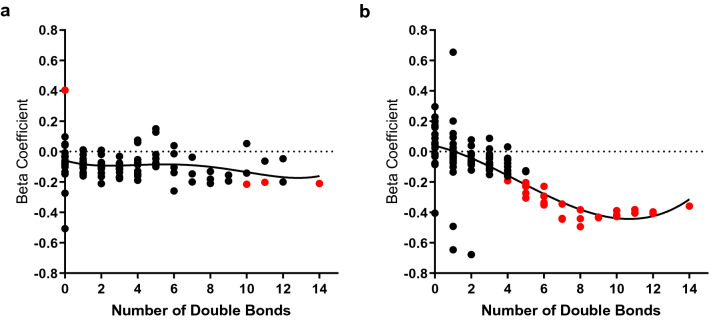
Association between triglycerides and birth weight in male and female infants. Regression models estimated the linear relationship between cord blood individual triglycerides and Fenton BW z score in (**a**) males and (**b**) females, adjusting for maternal age, parity, gestational age, baseline BMI, and GWG. Beta coefficients are plotted by the number of double bonds in the fatty acid tails of the TGs. Significant TGs are marked in red (α = 0.05). Non-parametric regression fit polynomial lines for each plot.

Within the CB, all LysoPC and LysoPE lipids groups were positively associated with BW (FDR < 0.1), independent of the number of double bonds, consistent in both male and female infants (Fig. 4c). Additionally, CB DG-poly and TG-poly were inversely associated with BW (FDR < 0.1), driven by female infants, and CE-poly was inversely associated with BW (unadjusted p-value < 0.05), driven by male infants. In response to the unique relationship between CB TG-poly and BW, sex-stratified regression models determined the relationship between individual CB TGs (97 triglycerides) and Fenton BW z score, adjusting for maternal age, parity, gestational age, GWG, and BMI (Supplementary Table S7). From the sex-stratified models, beta coefficients were plotted by the number of double bonds in the TGs (Fig. 5). Observing non-linear trends, we used non-parametric regression to fit polynomial lines to each curve, emphasizing the evident sex differences in the relationship between polyunsaturated TGs and BW, again driven by females.

### Influence of maternal lipidome on cord blood lysophospholipids

**Figure 6:**
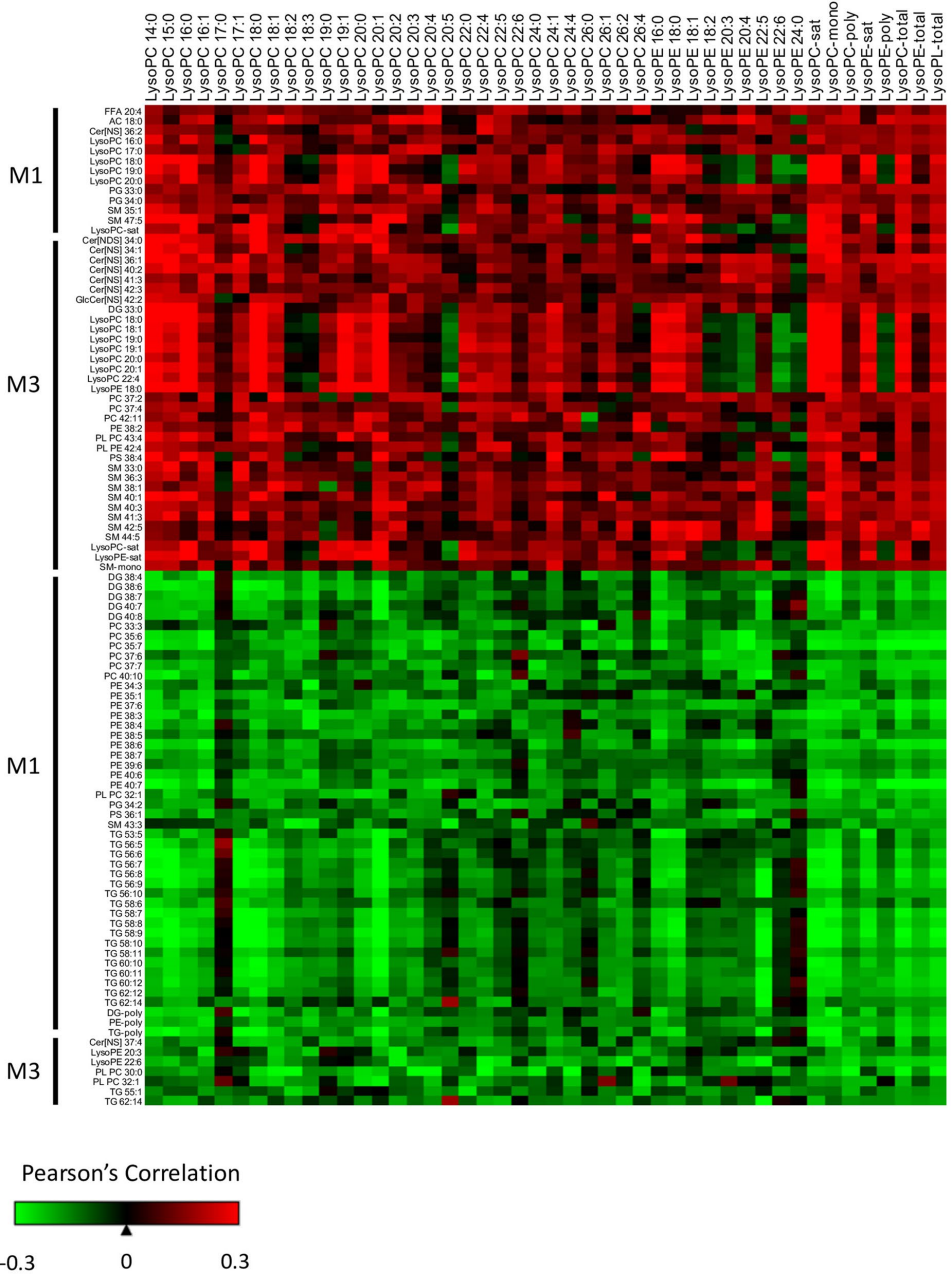
Maternal lipids correlate with cord blood lysophospholipids. The association between maternal individual lipids and lipid groups with CB LysoPCs, LysoPEs, and total LysoPLs was classified using Pearson’s correlations. Maternal lipids listed were significantly correlated with the lipid group CB LysoPL-total (unadjusted p-value α = 0.05). Maternal lipids (rows) are broken down by positive and inverse associations. Cord blood lysophospholipids (columns) are ordered by increasing chain length and number of double bonds. CB lipids groups LysoPC-sat, LysoPC-mono, LysoPC-poly, LysoPE-sat, LysoPE-poly, LysoPC-total, LysoPE-total, and LysoPL-total are listed in the last columns.

Our results indicated that CB LysoPLs of varying chain length and number of double bonds are elevated in the CB compared to M3 (Fig. 2) and are significantly associated with BW, independent of sex (Fig. 4). To assess if maternal lipids during pregnancy might influence the level of CB LysoPLs, the M1 and M3 lipidome was correlated with individual CB LysoPCs and LysoPEs, as well as the unsupervised Principal Component Analysis scores of LysoPC and LysoPE subgroups (Sat, Mono and Poly) and all LysoPLs (LysoPL-total). Lipids from M1 and M3 that were significantly correlated with the CB LysoPL-total group are displayed in Fig. 6. In M1, 13 lipids positively correlated and 47 lipids inversely correlated with CB total LysoPL levels. Lipids with a positive correlation include AC 18:0 and LysoPC species (16:0, 17:0, 18:0, 19:0, and 20:0), as well as the saturated LysoPC lipid group. Lipids with an inverse correlation mainly include individual lipids and lipid groups with PUFA containing DG, PC, phosphatidylethanolamine (PE), and TGs, highlighting the association between early maternal levels of PUFAs within multiple lipid classes and CB LysoPL-total.

In M3, 34 lipids were positively correlated, and 7 lipids were inversely correlated with CB LysoPL levels. M3 saturated LysoPC and LysoPE lipid groups, as well as individual LysoPL, were positively associated with CB LysoPL-total, implying that higher M3 levels results in higher CB levels. CER and SM containing saturated and monounsaturated TGs were positively associated with CB LysoPL-total. Interestingly, inverse associations were observed between M3 LysoPE 20:3 and 22:5 with CB LysoPL-total, suggesting differences based on the fatty acid tail.

Most correlations between the significant M1 and M3 individual lipids and individual CB LysoPCs and LysoPEs were in the same direction as CB LysoPL-total. Subtle variations in the correlation patterns are observed for LysoPC 18:2, 18:3, 20:5, and 26:4 and LysoPE 18:2, 20:3, 20:4, 22:6, and 24:0, potentially suggesting differences in how the maternal lipidome modulates these CB polyunsaturated LysoPLs.

### Influence of the maternal lipidome on cord blood polyunsaturated triglycerides

**Figure 7:**
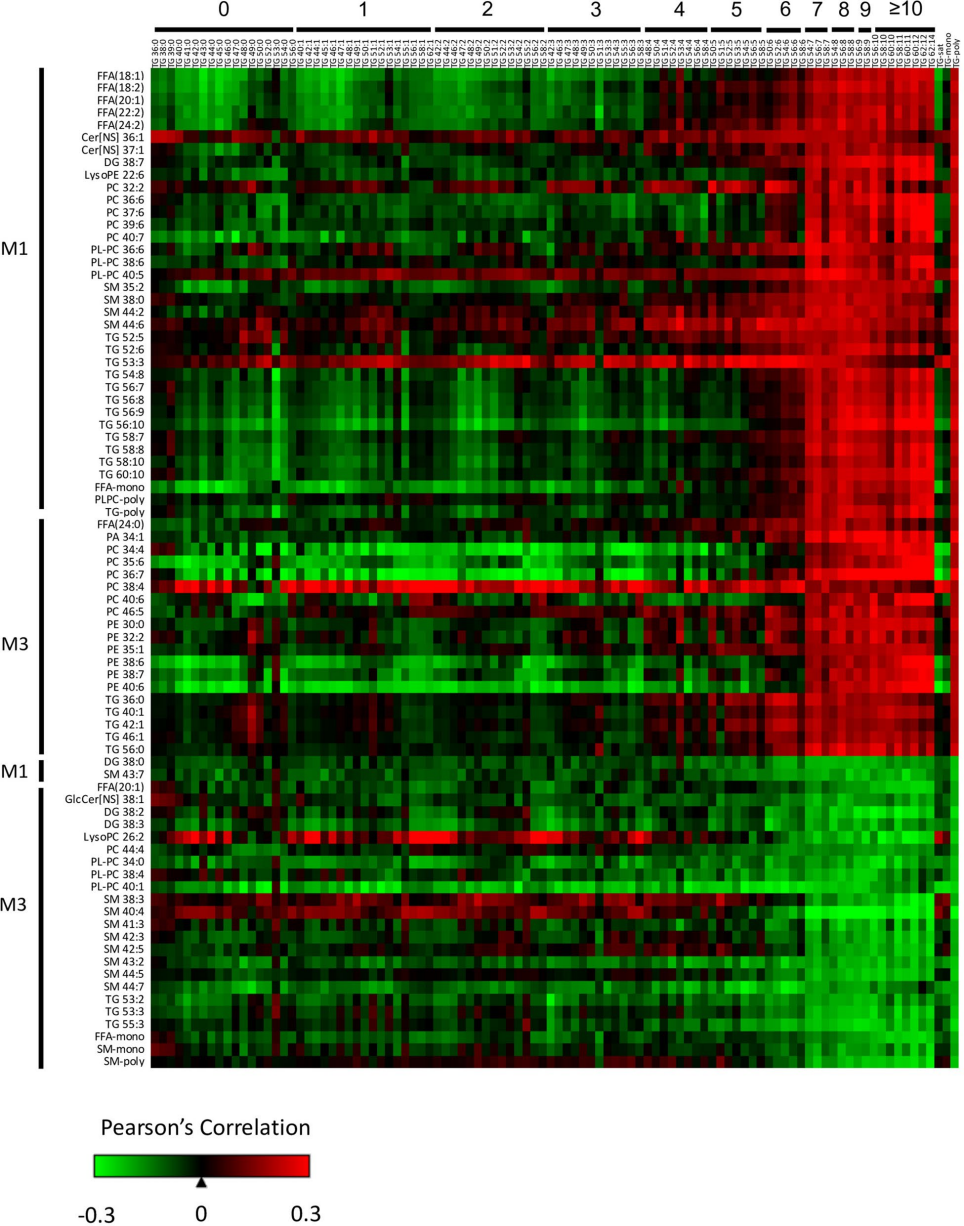
Maternal lipids correlate with cord blood polyunsaturated triglycerides. The association between maternal individual lipids and lipid groups with CB TGs was classified using Pearson’s correlations. Maternal lipids listed were significantly correlated with the lipid group CB TG-poly (unadjusted p-value α = 0.05). Maternal lipids (rows) are broken down by positive and inverse associations. Cord blood triglycerides (columns) are ordered by increasing chain length and number of double bonds. CB lipids groups TG-sat, TG-mono, and TG-poly are listed as the last three columns.

The apparent preferential transfer of lipids containing PUFAs into the CB (Fig. 2) and an inverse association between Fenton BW z score and CB lipid groups containing PUFAs (Figs. 4, 5) prompted us to investigate which maternal lipids are associated with the levels of PUFAs in CB. The M1 and M3 lipidome was correlated with CB TGs. Lipids from M1 and M3 that were significantly correlated with the CB TG-poly group are displayed in Fig. 7. A variety of M1 PUFA-containing lipids, including PC, PL-PC, DG, and TG, were positively associated with CB TG-poly. In M3, there were positive correlations between PCs and PEs, 75% of which contained PUFAs, with CB TG-poly, suggesting the mobilization of PUFAs within PCs and PEs to the placenta for transport. Lastly, M3 monounsaturated and polyunsaturated SM were inversely associated with CB TG-poly. Note that these lipids were found to be positively associated with maternal BMI (Supplementary Figure S3) and Fenton BW z score (Fig. 4).

## Discussion

Using a comprehensive lipidomics platform including almost 600 lipid species, this study objectively classified the maternal metabolic environment during the first trimester and at delivery as well as the infant lipidome, reflecting infant metabolism. We found dynamic shifts in the maternal lipidome across pregnancy and evidence for selective transfer of lipids containing PUFA from maternal to fetal circulation. We further identified associations between the lipidome and BW, emphasizing the inverse correlations observed with CB DGs and TGs containing PUFAs and positive correlations observed with CB LysoPL. Lastly, we identified maternal lipids which may modulate CB lipid levels that are influential in growth.

Early in gestation, insulin sensitivity is enhanced to increase maternal energy storage^29^. However, as pregnancy advances, maternal insulin resistance increases with elevations in lipid parameters such as lipoproteins, TGs, and total cholesterol to support the necessary metabolic changes for pregnancy maintenance and fetal growth^9^. Of the 573 lipids analyzed, 35% were significantly increased while only 10% were decreased in M3 compared to M1 (Fig. 2b, Supplementary Table S1). Between M1 and M3, we observed higher levels of PC, PE, DG, CER, SM, TG, and FFA, many containing fatty acids with 16–18 carbons. The majority of lipid species reduced in M3 are increased in CB and are enriched in long-chain PUFAs, suggesting specific transfer of these entities across the placenta to the fetus to provide LC-PUFAs to support brain development^30^. While several studies have demonstrated the apparent transfer of individual PUFAs into the fetal circulation during the later stages of pregnancy^26,31,32^, our study is the first to document the transfer of a wide variety of lipid species containing LC-PUFA including CE, LysoPL, TG, and SM as well as free LC-PUFA.

The transport of PUFA from maternal circulation to the fetus is dependent upon fetal requirements of PUFAs, which increase during late gestation. PUFAs such as arachidonic acid (20:4n-6) (AA) and docosahexaenoic acid (22:6n-3) (DHA) are essential for establishing cell membranes in the brain and retina^33^. Within the maternal liver, these fatty acids are derived from the essential fatty acids (EFA) linoleic acid and alpha-linolenic acid via elongation by liver Δ5 and Δ6 desaturases. Fetal supply of very LC-PUFAs is dependent on placental transfer because placenta lacks the enzymes Δ5 and Δ6 desaturases^34^ and the fetus only has limited desaturase activity^35^.

Correlations between the maternal and CB lipidome can indicate the role of transport proteins. Our results suggest that starting early in gestation, M1 PUFA levels within PC, PL-PC, DG, and TG are positively associated with CB TG-poly (Fig. 7). Therefore, maternal PUFA levels early in gestation are crucial to establish infant PUFA levels. Since there is no evidence of direct placental transport of TG from maternal to fetal circulation^36^, these results suggest a diversion of PUFAs from the maternal TG pool with re-esterification in CB TGs. This diversion could occur during hepatic TG elongation and desaturation of EFAs, with increased esterification of PUFA or by TG lipolysis in placenta and selective uptake of PUFA at the maternal–fetal interface^37,38^. Maternal fatty acids are transported to the fetus across the placenta via a variety of fatty acid transport proteins as well as passive diffusion^39^.

Using ^13^C-labeled fatty acids administered to women 12-h prior to scheduled Cesarean delivery, Gil-Sánchez et al. demonstrated that the esterification of fatty acids into different lipid fractions influences placental transfer to the fetus^39^. In maternal plasma at the time of delivery, ^13^C-fatty acid enrichment was detected with the incorporation of saturated fatty acid ^13^C-palmitate and monounsaturated fatty acid ^13^C-oleate in TGs while the PUFAs ^13^C-linoleic and ^13^C-DHA were found in both TGs and phospholipids^39^. Within the placenta, 90% of labeled FA were found in the phospholipid fraction with significant enrichment of ^13^C-DHA in CB compared to both maternal and placental samples. These results suggest that the incorporation of essential PUFA into maternal phospholipids may be important for placental transfer at the end of gestation. Our results found M3 polyunsaturated FA within PCs and PEs, rather than DGs and TGs, are positively associated with CB TG-poly (Fig. 7), supporting the importance of the phospholipid fraction in the transfer of PUFA late in gestation.

Our results indicated a sexual dimorphic effect of CB DG- and TG-poly with BW, with a larger effect seen in the females (Figs. 4, 5). Previous studies that have looked at the effect of PUFA supplementation during pregnancy on infant BW have yielded mixed results^40^, potentially due to differences in gestation length, baseline characteristics of the participants, and source, timing, and dose of n-3 PUFA supplement. PUFA supplementation has been associated with longer gestation and an increase in fetal weight, especially in women at risk for intrauterine growth retardation^41^. However, O’Tierney-Ginn, et al.^42^ found that total CB saturated fatty acids was positively associated with high skinfold thickness at birth (but not birthweight BMI z score), as well as increased BMI-z trajectory in early infancy, while PUFA levels were inversely correlated with BMI-z trajectory. Hauner, et al.^43^ demonstrated that dietary n-3 LC-PUFA supplementation of women had no effect on BW in a randomized trial of 208 women but had a larger effect on the gene expression profile in female placentas. Sedlmeier et al. observed placental gene expression to be more responsive to maternal omega-3 supplementation in females than males^44^. The reason for the sexually dimorphic differences is not yet clear.

We observed a positive association between CB LysoPE and LysoPCs and Fenton BW z score (Fig. 4), independent of the chain length and the number of double bonds. This confirms findings from other studies that CB LysoPCs with varying chain length and saturation are positively associated with newborn BW^22–24^, as well as with weight at age 6 months^22^. The associations between LysoPL and BW were not as apparent in M1 and M3, with an exception being M3 LysoPE-sat being positively associated with BW (Fig. 4). Additionally, we found that maternal GWG is positively associated with M1 and M3 LysoPC and LysoPE lipid groups of varying number of double bonds (Supplementary Table S4).

Recently, the Major Facilitator Superfamily Domain Containing 2a (MFSD2a) protein has been proposed as a NA^+^-dependent LysoPL transporter in the brain^45^ and placenta^46^. This transporter provides a mechanism for the active transport of LysoPL from maternal plasma, consistent with our study findings that (1) LysoPC and LysoPE are depleted in maternal plasma and elevated in CB (Fig. 2) and (2) M1 and M3 LysoPLs, mainly containing saturated FA, are positively correlated with total CB LysoPL levels (Fig. 6).

Since the sn-2 position of phospholipids tends to be occupied by PUFAs^47^, it has been hypothesized that specific placental endothelial lipases cleave sn-1 fatty acids, allowing transport of the PUFA-containing LysoPL to the fetal circulation, perhaps enhancing delivery to the developing fetal brain^27,48^. An intriguing possibility suggested by our data is that there is an interaction between PUFA during development and later transfer of LysoPL to the fetus. We see a significant inverse association of PUFA-containing lipids M1 and LysoPC levels in the CB of infants and conversely, a positive association of M1 (and M3) lipids enriched in saturated fatty acids (Fig. 6). This might imply early programming of LysoPL transport capacity. Control of MFSD2a expression in placenta has not been established, but is potentially regulated by diet. In mice, cortical and subcortical brain MFSD2a levels increased in rodents fed a high fat diet, with PUFA-containing diets having a greater effect at lower concentrations than a lard-containing diet^49^. Additionally, MFSD2a expression in placenta is positively correlated with CB DHA levels^46^, supporting its role in PUFA transport and implying it responds to the levels of DHA.

Of interest, polyunsaturated SM are decreased between M3 and CB (Fig. 2), suggesting that maternal late gestation SM may be (1) directly transferred to the fetus to provide PUFAs or (2) converted into LysoPL, the preferential transfer method of PUFAs. Multiple significant correlations were observed between M3 SM and the CB lipidome. CB polyunsaturated TGs are inversely correlated with M3 SM-mono and SM-poly individual lipids and lipid groups (Fig. 7), suggesting that elevated late gestation polyunsaturated SM may result in lower levels of polyunsaturated containing TG in the CB. Supporting these results, we found that M3 SM-mono and M3 SM-poly are positively associated with maternal baseline BMI (Supplementary Figure S4) and positively associated with newborn BW (Fig. 4). These results suggest that inability to convert SM to transfer the monounsaturated and polyunsaturated fatty acids across the placenta results in less favorable fetal development, potentially linked to maternal BMI. Lastly, M3 saturated and monounsaturated SM were positively associated with CB LysoPL-total levels. These results highlight the importance of the SM lipid fraction during gestation, which potentially is related to the pathogenesis of obesity, T2D, and various metabolic diseases^50^.

## Conclusions

The novel finding that the lipids that show the greatest differences between maternal and fetal circulation at term are also associated with BW suggests a complex interaction between PUFA intake, incorporation into specific lipid subtypes, and transport to the developing fetus. Future longitudinal studies with a larger sample size are desired to assess if changing the maternal metabolic environment to a more favorable lipidome will improve infant outcomes. It would be of particular interest to further explore the sex-specific influence of the maternal lipidome on the developing fetus.^51^ Collection of maternal dietary intake can help elucidate the relationship between nutrient intake and blood concentrations of very long-chain FA, as the metabolome does not solely represent dietary intake, but rather a combination of genetics, diet, and phenotypes. Maternal blood collection at delivery may not be representative of the metabolic states during the third trimester due to the stress of parturition, although we observed no differences in lipidome components based on route of delivery.

While our results are consistent with previous studies,^10,21,23^ a potential weakness of our work is the fact that the pragmatic recruitment of women during their first prenatal visit (M1) resulted in variable post-prandial times. In addition, it is obviously difficult to control the fed/fasted state in women being admitted into the hospital with spontaneous labor. In M1, the main associations of interest included lipid classes with polyunsaturated fatty acid tails (Figs. 6, 7). The levels of PUFAs in peripheral blood reflect the chronic intake of PUFAs^52^ and many of the lipid fractions with PUFAs were correlated between M1 and M3 (Supplementary Fig. S2), suggesting a relatively constant intake of PUFAs across pregnancy. Finally, while small, the meal-induced changes that can alter the levels of lipid species in the blood^53^ along with the variable post-prandial time would be expected to weaken any association. Despite these confounding factors, we do see robust association of PUFA-containing TGs, PCs and PEs and CB lipid related to birth weight.

Collecting multiple fasting blood samples throughout pregnancy will help to further classify lipidome changes throughout pregnancy. Collection of placenta samples and quantifying gene expression of key transport proteins (i.e. MFSD2a) would provide details on preferential transfer of lipid species. The results of this study indicate that starting in early gestation, the maternal metabolite environment may have an important effect on offspring weight and potentially, long-term cardiometabolic risk, consistent with developmental origins of health and disease^1^.

## Methods

### Michigan mother-infant pairs cohort (MMIP)

The subjects that participated in this study are part of the Michigan Mother-Infant Pair (MMIP) birth cohort study (2010–present). Briefly, pregnant women were recruited at their first prenatal appointment between 8 and 14 weeks of gestation. Eligibility criteria for MMIP include: age between 18 and 42 years old, had a spontaneously conceived singleton pregnancy, and intended to deliver at the University of Michigan Hospital. A subset of MMIP participants were chosen for lipidomics measures. Criteria for inclusion for the current study include the mother-infant pairs having complete demographic, survey and health information at their initial study visit and availability of all biospecimen at all-time points from mother and child. The MMIP study was approved by the University of Michigan Institutional Review Board (HUM00017941). The study was carried out in accordance with the approved guidelines and regulations. Written informed consent was received from all participants and all plasma samples and metadata were deidentified prior to analysis.

During the initial study visit at 8–14 weeks of pregnancy, participants provided a blood sample (M1) and weight and height were collected by a clinician to calculate their baseline BMI (kg/m^2^). Maternal weight at delivery was measured to calculate gestational weight gain (GWG) between the initial visit and delivery. Maternal venous blood samples (M3) and cord blood (CB) samples via venipuncture from the umbilical cord were collected. Women were not required to be fasted prior to blood sampling (M1 and M3). Physician-measured infant BW and head circumference were adjusted for gestation age and infant sex using Fenton growth curves for BW^54^ and growth curves developed by the Canadian Institute of Health Research for head circumference^55^. Additional maternal information collected includes race/ethnicity, parity, marital status, and delivery route (vaginal and planned or unplanned Cesarean section). All 106 mother-infant pairs met additional inclusion criteria for this study including infant BW greater than 2,500 g, maternal BMI > 18.5, full term, and no pregnancy complications (i.e. gestational diabetes mellitus).

### Lipidomics methodology

Plasma samples were stored at − 80 °C prior to lipidomics analysis. Untargeted shotgun lipidomics was performed as previously detailed^56^ using reconstituted lipid extract from M1, M3, and CB plasma samples. Samples were ionized in positive and negative ionization model using a Triple TOF 5600 (AB Sciex, Concord Canada). Chromatographic peaks that represent features were detected using a modified version of existing commercial software (Agilent MassHunter Qualitative Analysis). Pooled human plasma samples and pooled experimental samples were randomized and run for quality control across batches. Data normalization followed a recently described drift removal method^57^. Detected features were excluded with (1) a relative standard deviation greater than 40% in the pooled samples and/or (2) less than 70% presence in the samples. Missing data were imputed using the K-nearest neighbor method.

Lipids were classified using LIPIDBLAST, a computer-generated tandem mass spectral library containing 119,200 compounds from 26 lipid classes^58^. All lipids are annotated by the nomenclature X:Y, where X is the sum length of the fatty acid carbon chain and Y, the number of double bonds. After data processing, 573 biological lipids were identified, consisting of free fatty acids (FFA), cholesteryl esters (CE), acylcarnitines (AC), ceramides (CER), diacylglycerols (DG), lysophosphatidylcholine (LysoPC), lysophosphatidylethanolamine (LysoPE), phosphatidic acid (PA), phosphatidylcholine (PC), phosphatidylethanolamine (PE), plasmenyl-phosphatidylcholine (PL-PC), plasmenyl-phosphatidylethanolamine (PL-PE), phosphatidylglycerol (PG), phosphatidylinositol (PI), phosphatidylserine (PS), sphingomyelins (SM), and triglycerides (TG). Lipidomics methodology depicted in Fig. 1b.

### Statistics

Prior to the main analysis, we examined differences in maternal and newborn characteristics stratified by sex of the newborn. Pearson chi-square tests were used to identify sex differences in categorical variables. Unpaired t-tests were used to identify sex differences in continuous variables. Scatterplots depicted the linear relationship between maternal baseline BMI, gestational weight gain, and Fenton BW percentile, highlighting the slope of the best-fit line (beta coefficient) and the tightness (r^2^) of the trend.

Step 1: To observe changes in the lipid profile across pregnancy, raw peak intensities of each lipid were standardized (mean = 0, standard deviation = 1) across M1, M3, and CB. Paired t-tests were used to identify differences in raw peak intensities of individual lipids between M1 and M3, to represent change in maternal metabolome during pregnancy, and M3–CB, to represent transfer of lipids through the placenta. We calculated FC for M1–M3 (log2[M3/M1]) and for M3–CB (log2[CB/M3]) to quantify change between time points. Differential lipids were classified using a Bonferroni p-value adjustment accounting for the total number of lipids (α = 0.05/573).

Step 2: Due to biological constraints on metabolism, many metabolites are highly correlated. In response, we used prior knowledge to create lipid groups. Lipids were grouped by their class and number of double bonds, yielding 41 groups (Supplementary Table S2). For each lipid group, scores were created using unsupervised principal component analysis. The first principal component was retained, accounting for the largest portion of variance using PROC FACTOR in SAS. Within the principal component, each lipid receives a factor loading, which is the correlation coefficient between the lipid and the component. Scores were created by (1) multiplying the lipid factor loading by the lipid standardized peak intensity and (2) adding together these values for all lipids within a lipid group, using PROC SCORE in SAS. Mother-infant dyads received lipid group scores for M1, M3, and CB, separately. Using Debiased Sparse Partial Correlations^28^, the correlations between lipid groups within and between time points were measured. Significantly correlated lipid groups have a false discovery rate (FDR) adjusted p value < 0.1.

Step 3: Linear regression was used to identify the relationship between maternal baseline BMI and GWG with M1, M3, and CB lipid groups, adjusting for sex, parity, maternal age, and gestational age. Linear regression was used to identify M1, M3, and CB lipid groups associated with Fenton BW z score, adjusting for sex, parity, maternal age, gestational age, maternal baseline BMI, and GWG. Sex-stratified models were run. Associations were labeled using a FDR adjusted p value < 0.1 and, for exploratory analyses, an unadjusted p value < 0.05. Furthermore, linear models were used to explore sex differences between individual CB triglycerides (mean 0, standard deviation 1) and Fenton BW z score, stratified by infant sex and adjusted for parity, maternal age, gestational age, maternal baseline BMI, and GWG. Non-parametric regression curves, classified using varying coefficient models, were plotted to classify how the relationship between TGs and BW is related to the number of double bonds in TGs (R package ‘np’).

Step 4: Lastly, we sought to identify maternal lipids related to infant CB lipid groups associated with Fenton BW. CB lysophospholipids and triglycerides were correlated with M1 and M3 individual lipids and lipid groups to determine if maternal lipids modulate the levels with CB, using Pearson's correlations (unadjusted p value < 0.05).

Statistical methodology depicted in Fig. 1c. Unless otherwise stated, all statistical analyses were performed using SAS 9.4 (Cary, North Carolina). Figures were created using GraphPad Prism version 7.4 (La Jolla, California). Heatmaps were created using an in-house software package, CoolMap.

## Supplementary information

Supplementary Information
