# Supplementary material for: Maternal lipid levels across pregnancy impact the umbilical cord blood lipidome and infant birth weight
Source: Sci Rep. 2020 Aug 26;10:14209. doi: 10.1038/s41598-020-71081-z (PMC7449968; doi:10.1038/s41598-020-71081-z)
Supplement: Supplementary file 1 — Supplementary Information [file 41598_2020_71081_MOESM1_ESM.pdf]

TITLE PAGE

Maternal lipid levels across pregnancy impact the umbilical cord blood lipidome and infant birth weight

Jennifer L. LaBarre[1], Muraly Puttabyatappa[2], Peter X.K. Song[3], Jaclyn M. Goodrich[4], Ling Zhou[5], Thekkelnaycke M. Rajendiran[6,7], Tanu Soni[6], Steven E. Domino[8], Marjorie C. Treadwell[8], Dana C. Dolinoy[1,4], Vasantha Padmanabhan[1,2,4+], and Charles F. Burant[1,9,+,*]

Author Affiliations

[1]Department of Nutritional Sciences, University of Michigan School of Public Health, Ann Arbor MI, USA
[2]Department of Pediatrics, University of Michigan, Ann Arbor MI, USA
[3]Department of Biostatistics, University of Michigan School of Public Health, Ann Arbor MI, USA
[4]Department of Environmental Health Sciences, University of Michigan School of Public Health, Ann Arbor MI, USA
[5]Center of Statistical Research, Southwestern University of Finance and Economics, Chengdu, Sichuan, China
[6]Michigan Regional Comprehensive Metabolomics Resource Core, Ann Arbor, MI, USA.
[7]Department of Pathology, Michigan Regional Comprehensive Metabolomics Resource Core, Ann Arbor, MI, USA.
[8]Department of Obstetrics and Gynecology, University of Michigan Medical School, Ann Arbor MI, USA
[9]Department of Internal Medicine, University of Michigan Medical School, Ann Arbor MI, USA
+ These authors contributed equally to this work

*** Corresponding author information:**
Charles Burant, MD, PhD
6309 Brehm Tower
1000 Wall St.
Ann Arbor, MI, 48105-7415
Tel: 734-615-3481
Email: burantc@umich.edu

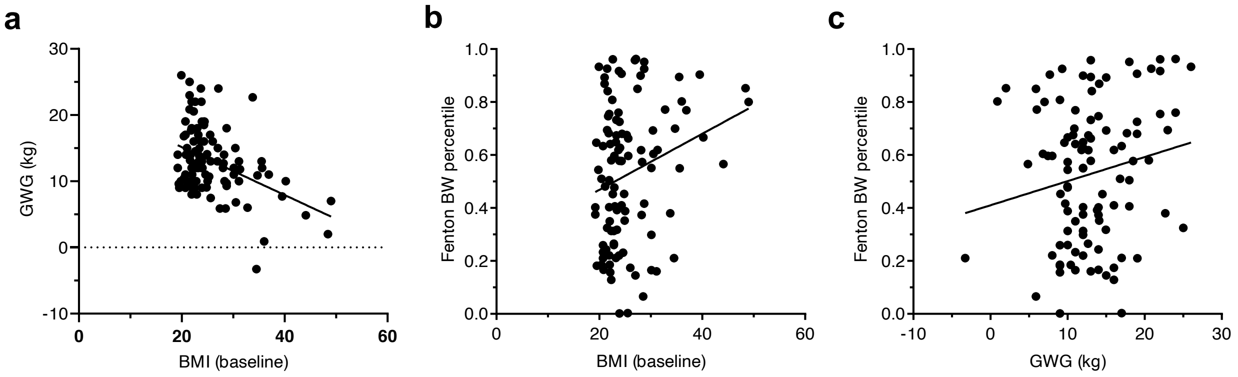

Supplementary Figure S1. Relationship between maternal baseline BMI, gestational weight gain, and Fenton BW percentile. (a) Maternal baseline BMI is inversely associated with gestational weight gain (beta=-0.360, r^2= 0.18, p<0.001). **(b)** Maternal baseline BMI is positively associated with Fenton BW percentile (beta= 0.011, r^2 = 0.06, p=0.0014). **(c)** Maternal gestational weight gain is trending towards a positive association with Fenton BW percentile (beta= 0.009, r^2= 0.03, p=0.070).

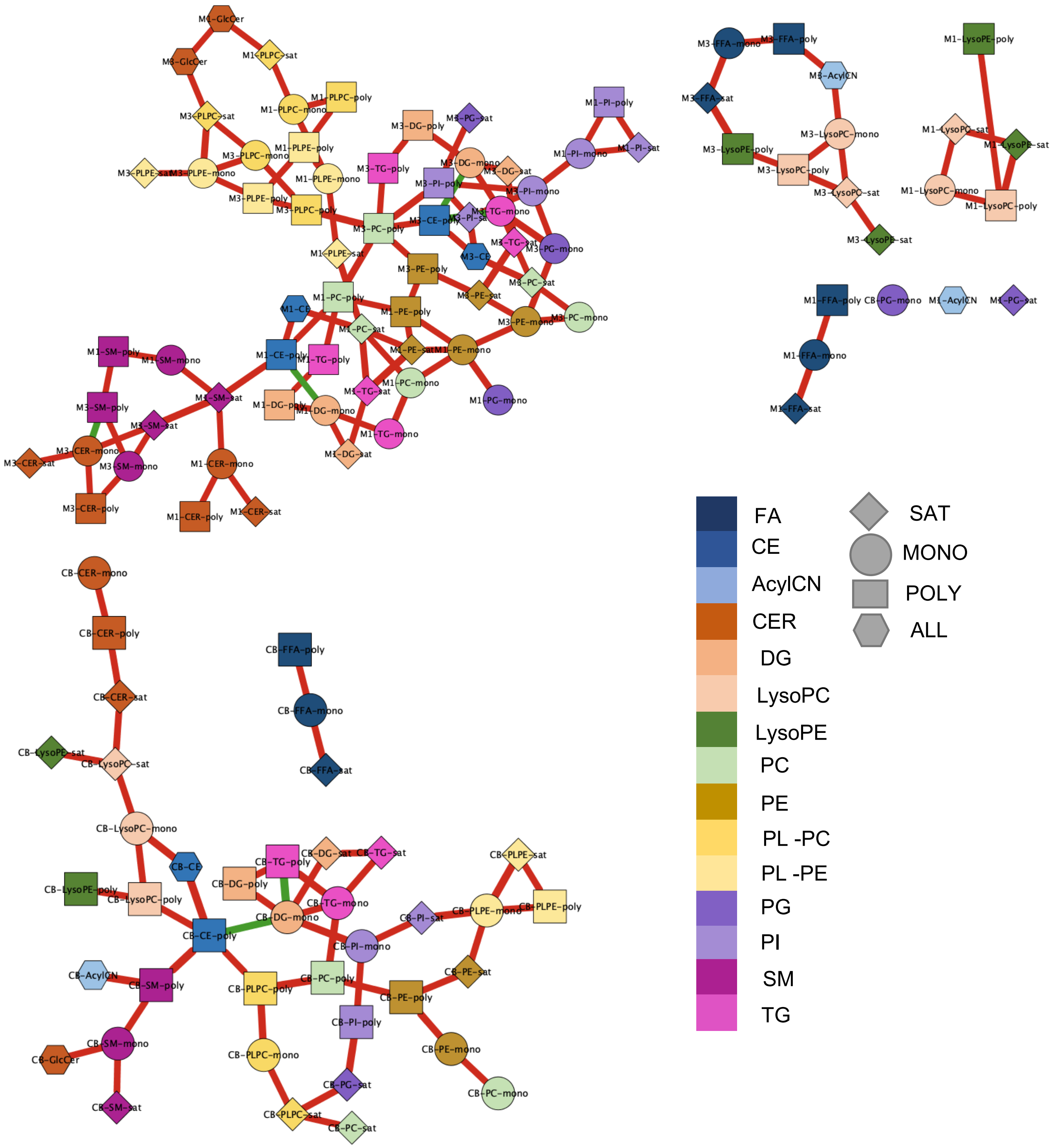

M1-GlcCer
M3-GlcCer
M1-PLPC-sat
M1-PLPC-poly
M1-PLPC-mono
M3-PLPC-sat
M3-PLPC-mono
M1-PLPE-poly
M3-PLPE-sat
M3-PLPE-mono
M1-PLPE-mono
M3-PLPE-poly
M3-PLPC-poly
M3-DG-poly
M3-PG-sat
M3-TG-poly
M3-DG-mono
M3-DG-sat
M3-PI-poly
M1-PI-poly
M1-PI-mono
M1-PI-sat
M3-PI-mono
M3-TG-mono
M3-PI-sat
M3-CE-poly
M3-PC-poly
M1-PLPE-sat
M3-TG-sat
M3-PG-mono
M3-CE
M3-PE-poly
M3-PC-sat
M3-PE-sat
M3-PE-mono
M3-PC-mono
M1-PC-poly
M1-CE
M1-PE-poly
M1-PC-sat
M1-CE-poly
M1-TG-poly
M1-PE-sat
M1-PE-mono
M1-PC-mono
M1-TG-sat
M1-DG-poly
M1-DG-mono
M1-TG-mono
M1-DG-sat
M1-PG-mono
M1-SM-poly
M1-SM-mono
M1-SM-sat
M3-SM-poly
M3-SM-sat
M3-CER-mono
M3-CER-sat
M3-SM-mono
M3-CER-poly
M1-CER-mono
M1-CER-poly
M1-CER-sat
M3-FFA-mono
M3-FFA-poly
M3-AcylCN
M3-FFA-sat
M3-LysoPC-mono
M3-LysoPE-poly
M3-LysoPC-poly
M3-LysoPC-sat
M3-LysoPE-sat
M1-LysoPE-poly
M1-LysoPC-sat
M1-LysoPE-sat
M1-LysoPC-mono
M1-LysoPC-poly
M1-FFA-poly
CB-PG-mono
M1-AcylCN
M1-PG-sat
M1-FFA-mono
M1-FFA-sat
CB-CER-mono
CB-CER-poly
CB-CER-sat
CB-LysoPE-sat
CB-LysoPC-sat
CB-LysoPC-mono
CB-CE
CB-LysoPE-poly
CB-LysoPC-poly
CB-TG-poly
CB-DG-sat
CB-TG-sat
CB-DG-poly
CB-TG-mono
CB-DG-mono
CB-PLPE-sat
CB-PLPE-mono
CB-PLPE-poly
CB-PI-sat
CB-CE-poly
CB-PI-mono
CB-AcylCN
CB-SM-poly
CB-PLPC-poly
CB-PC-poly
CB-PE-sat
CB-PE-poly
CB-PI-poly
CB-SM-mono
CB-GlcCer
CB-PLPC-mono
CB-PE-mono
CB-PG-sat
CB-SM-sat
CB-PLPC-sat
CB-PC-sat
CB-PC-mono
CB-FFA-poly
CB-FFA-mono
CB-FFA-sat
FA
CE
AcylCN
CER
DG
LysoPC
LysoPE
PC
PE
PL -PC
PL -PE
PG
PI
SM
TG
SAT
MONO
POLY
ALL

Supplementary Figure 2. Lipid groups exhibit positive correlation between M1 and M3. Network demonstrates significant associations within time points and between time points (M1-M3 [8 edges], M1-CB [0 edges], and M3-CB [0 edges]). The relationship between lipid groups at M1, M3, and CB was estimated using Debiased Sparse Partial Correlations. Nodes were clustered organically, demonstrating connection of nodes between M1 and M3. Colors of nodes depict class of lipid. Shapes of nodes depict number of double bonds. Positive correlations are depicted using red edges. Inverse correlations are depicted using green edges. Edges represented have an adjusted p-value < 0.1.

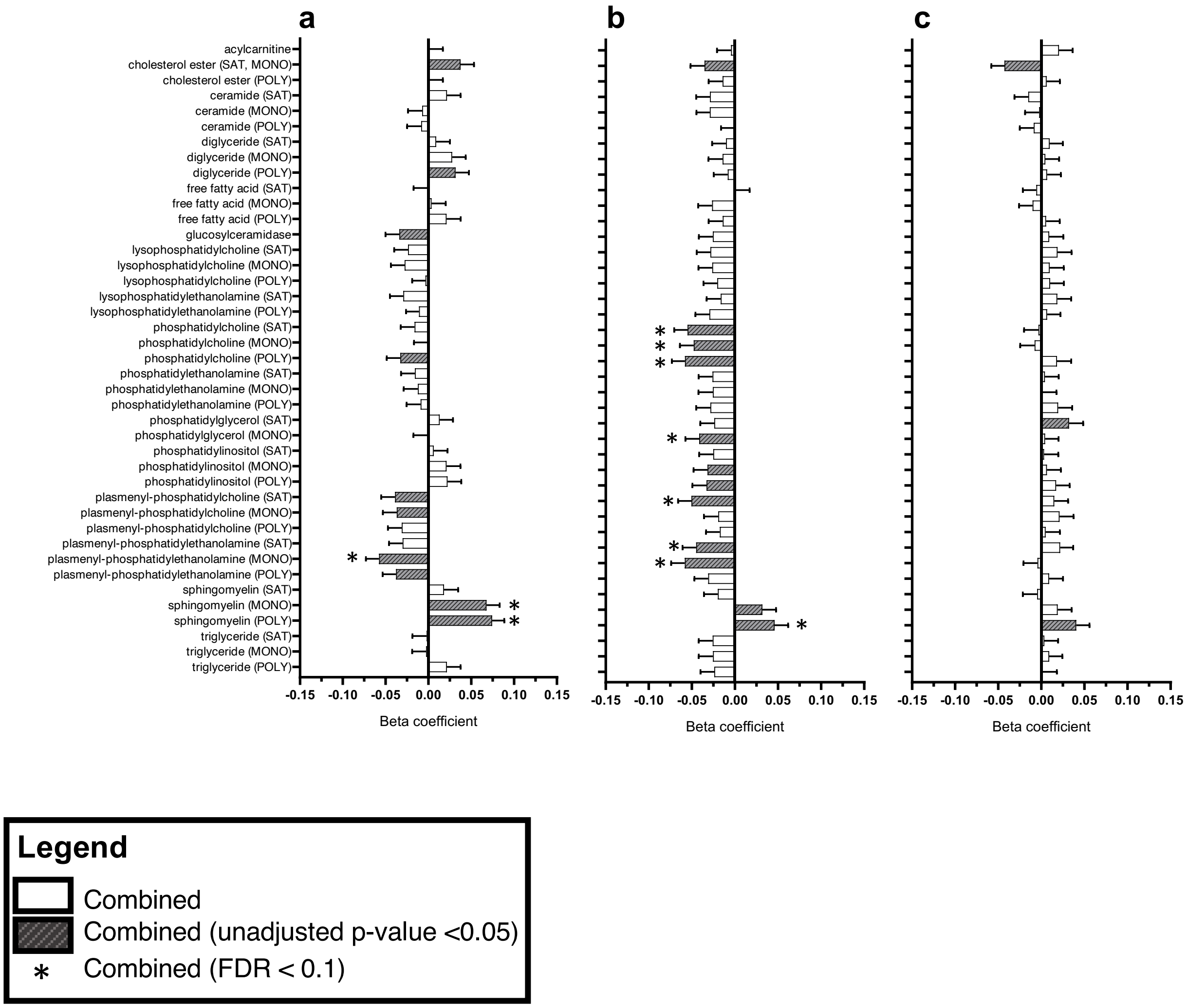

Supplementary Figure S3. Associations between maternal and cord blood lipid groups with maternal BMI at baseline. Regression models estimated the linear relationship between maternal BMI at baseline and **(a)** maternal first trimester, **(b)** maternal term, and **(c)** cord blood lipid groups, adjusting for sex, maternal age, parity, and gestational age. Beta coefficients plotted as white bars (β±SE) with significance depicted as gray stripped bars (unadjusted p-value α=0.05) and "*" (FDR <0.1).

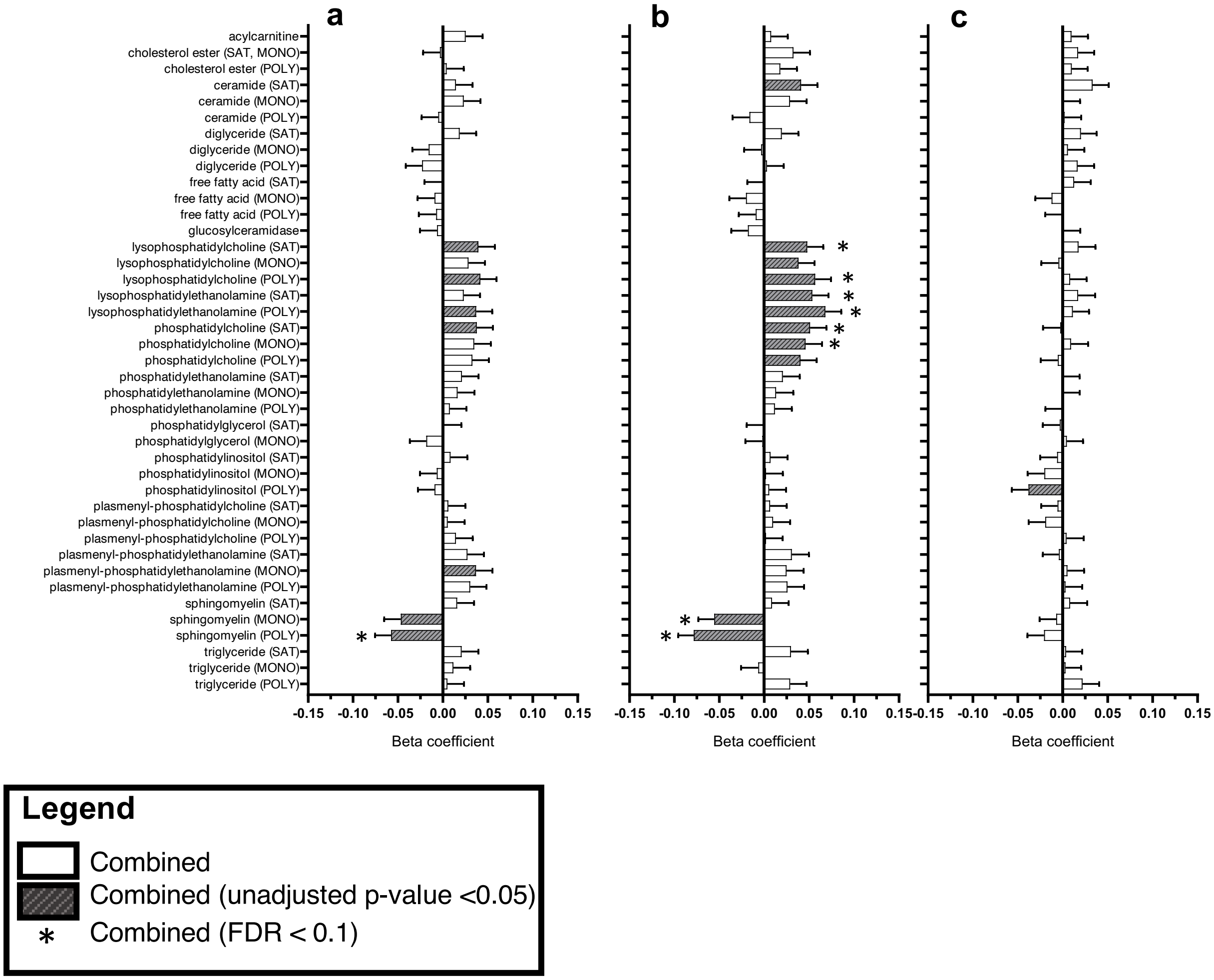

Supplementary Figure S4. Associations between maternal and cord blood lipid groups with maternal gestational weight gain. Regression models estimated the linear relationship between maternal GWG (kg) and **(a)** maternal first trimester, **(b)** maternal term, and **(c)** cord blood lipid groups, adjusting for sex, maternal age, parity, and gestational age. Beta coefficients plotted as white bars (β±SE) with significance depicted as gray stripped bars (unadjusted p-value α=0.05) and “*” (FDR<0.1).

Supplementary Table S1. Comparison of M1-M3 and M3-CB lipid raw peak intensities. Paired t-tests identified differences in raw peak intensities of individual plasma lipids between M1 and M3 and M3 to CB. The fold change between lipids in M1 and M3 was calculated by log2(M3/M1), where a positive FC indicates increases in that lipid between M1 and M3 and a negative FC indicates decreases in that lipid between M1 and M3. The fold change between lipids in M3 and CB was calculated by log2(CB/M3), where a positive FC indicates increases in that lipid between M3 and CB and a negative FC indicates decreases in that lipid between M3 and CB. Significance highlighted in red (Bonferroni adjusted, α=0.05/573).

Metabolite	M1		M3		CB		M1 M3 t-test			M3 CB t-test		
	Mean	Std dev	Mean	Std dev	Mean	Std dev	log2(M3/M1)	p-value	-log10(p-value)	log2(CB/M3)	p-value	-log10(p-value)
FFA(16:0)	5.68E+07	8.07E+06	7.06E+07	1.39E+07	6.08E+07	1.22E+07	0.314	3.46E-16	15.461	-0.217	3.89E-09	8.410
FFA(18:0)	6.89E+07	5.46E+06	7.23E+07	7.23E+06	6.83E+07	1.13E+07	0.071	1.13E-04	3.948	-0.083	6.06E-05	4.218
FFA(18:1)	2.37E+07	1.42E+07	5.46E+07	2.45E+07	2.18E+07	9.47E+06	1.203	3.16E-23	22.501	-1.324	5.17E-29	28.287
FFA(18:2)	1.62E+07	8.75E+06	3.37E+07	1.50E+07	1.43E+07	6.64E+06	1.055	1.29E-20	19.889	-1.232	7.54E-27	26.123
FFA(20:0)	1.99E+06	2.46E+05	2.10E+06	3.40E+05	1.86E+06	3.80E+05	0.083	4.17E-03	2.380	-0.178	6.75E-08	7.171
FFA(20:1)	4.66E+05	2.38E+05	1.34E+06	6.81E+05	3.65E+05	1.28E+05	1.526	3.46E-27	26.461	-1.880	6.52E-34	33.185
FFA(20:2)	4.17E+05	2.33E+05	9.58E+05	4.24E+05	4.79E+05	1.87E+05	1.199	4.63E-24	23.335	-1.001	3.69E-22	21.433
FFA(20:4) Arachidonic acid	1.45E+06	6.69E+05	1.73E+06	6.73E+05	2.61E+06	1.77E+06	0.260	2.22E-03	2.653	0.590	1.24E-05	4.908
FFA(22:0)	5.59E+05	1.43E+05	6.46E+05	2.71E+05	4.64E+05	1.45E+05	0.208	3.99E-03	2.399	-0.477	1.82E-09	8.741
FFA(22:1)	1.41E+05	4.34E+04	2.58E+05	1.57E+05	1.19E+05	4.13E+04	0.871	3.09E-12	11.510	-1.118	1.61E-16	15.792
FFA(22:2)	3.64E+04	1.30E+04	8.29E+04	3.39E+04	5.86E+04	2.78E+04	1.186	3.01E-29	28.521	-0.499	8.39E-07	6.076
FFA(22:3)	4.85E+04	1.69E+04	8.90E+04	3.17E+04	1.56E+05	6.06E+04	0.877	2.20E-24	23.657	0.810	4.24E-19	18.372
FFA(24:0)	3.76E+05	1.53E+05	3.86E+05	1.73E+05	3.73E+05	1.09E+05	0.039	6.47E-01	0.189	-0.049	3.45E-01	0.462
FFA(24:1)	2.62E+05	6.80E+04	3.83E+05	1.08E+05	3.24E+05	9.86E+04	0.548	7.64E-19	18.117	-0.243	1.16E-05	4.937
FFA(24:2)	7.47E+04	2.29E+04	1.21E+05	3.54E+04	1.62E+05	5.02E+04	0.690	4.02E-23	22.396	0.425	4.50E-10	9.347
FFA(24:3)	1.23E+04	4.71E+03	1.72E+04	6.76E+03	4.08E+04	1.46E+04	0.487	3.69E-09	8.433	1.245	7.19E-35	34.144
16:0 Cholesteryl ester	4.57E+04	1.20E+04	4.72E+04	1.76E+04	4.50E+04	2.03E+04	0.046	4.75E-01	0.324	-0.068	8.88E-01	0.052
16:1 Cholesteryl ester	2.22E+04	1.04E+04	3.32E+04	1.51E+04	6.15E+04	4.11E+04	0.582	2.96E-09	8.528	0.887	2.88E-13	12.540
17:1 Cholesteryl ester	6.55E+03	2.87E+03	7.14E+03	2.74E+03	7.54E+03	3.13E+03	0.123	1.32E-01	0.881	0.079	3.77E-01	0.424
18:0 Cholesteryl ester	1.89E+04	8.57E+03	1.78E+04	7.35E+03	2.84E+04	1.42E+04	-0.083	3.38E-01	0.471	0.670	1.49E-10	9.827
18:1 Cholesteryl ester	5.79E+05	1.19E+05	6.98E+05	1.59E+05	4.44E+05	1.15E+05	0.269	3.76E-09	8.424	-0.652	3.31E-31	30.480
18:2 Cholesteryl ester	3.30E+06	5.46E+05	3.19E+06	6.38E+05	1.15E+06	3.77E+05	-0.048	1.91E-01	0.720	-1.477	6.08E-75	74.216
18:3 Cholesteryl ester	1.15E+04	2.65E+04	9.06E+03	2.09E+04	2.87E+03	4.94E+03	-0.339	4.65E-01	0.333	-1.658	4.15E-03	2.382
20:3 Cholesteryl ester	3.50E+05	8.97E+04	3.10E+05	8.99E+04	3.82E+05	9.38E+04	-0.175	1.37E-03	2.863	0.300	9.52E-07	6.021
20:4 Cholesteryl ester	1.48E+04	9.05E+03	1.67E+04	1.00E+04	9.32E+03	8.05E+03	0.175	1.47E-01	0.832	-0.843	2.14E-07	6.670
20:5 Cholesteryl ester	9.86E+04	7.82E+04	5.35E+04	5.23E+04	4.13E+04	4.71E+04	-0.883	1.63E-06	5.789	-0.371	3.27E-01	0.485
22:4 Cholesteryl ester	1.52E+04	5.45E+03	1.17E+04	4.62E+03	1.95E+04	6.98E+03	-0.384	6.65E-07	6.177	0.737	4.07E-17	16.391
22:5 Cholesteryl ester	4.07E+04	1.25E+04	3.39E+04	1.24E+04	7.05E+04	2.22E+04	-0.263	1.03E-04	3.985	1.056	1.38E-35	34.861
22:6 Cholesteryl ester	2.33E+05	8.96E+04	1.77E+05	8.09E+04	2.87E+05	1.10E+05	-0.398	3.11E-06	5.507	0.697	5.82E-14	13.235
Acylcarnitine 16:0	6.54E+04	2.02E+04	6.95E+04	2.48E+04	1.10E+05	4.37E+04	0.089	1.81E-01	0.741	0.666	1.04E-15	14.985
Acylcarnitine 18:0	3.10E+04	1.05E+04	2.88E+04	1.39E+04	3.10E+04	1.69E+04	-0.108	1.89E-01	0.723	0.107	3.94E-01	0.405
Acylcarnitine 18:1	1.22E+05	5.05E+04	1.32E+05	5.46E+04	1.04E+05	6.00E+04	0.111	1.79E-01	0.747	-0.345	3.72E-04	3.429
Acylcarnitine 18:2	7.63E+04	3.84E+04	7.12E+04	3.93E+04	8.88E+04	4.97E+04	-0.100	3.40E-01	0.469	0.318	8.42E-03	2.074
Acylcarnitine 24:0	1.82E+03	1.08E+03	1.86E+03	9.37E+02	1.15E+03	3.77E+05	0.031	7.77E-01	0.109	-0.690	2.28E-01	0.643
Acylcarnitine 26:0	2.69E+03	1.69E+03	2.53E+03	1.42E+03	1.89E+03	7.42E+03	-0.090	4.47E-01	0.350	-0.423	7.19E-01	0.144
Cer[NDS] 34:0	1.07E+05	2.19E+04	1.20E+05	2.95E+04	1.32E+05	4.24E+04	0.168	2.68E-04	3.572	0.135	4.09E-03	2.389
Cer[NDS] 36:0	3.58E+04	1.20E+04	7.37E+04	2.97E+04	7.24E+04	1.03E+05	1.042	4.28E-26	25.369	-0.024	2.21E-01	0.655
Cer[NDS] 38:0	3.60E+03	1.19E+03	5.22E+03	1.99E+03	5.68E+03	3.86E+03	0.536	1.19E-11	10.924	0.123	2.56E-02	1.592
Cer[NDS] 40:0	3.17E+05	1.07E+05	5.04E+05	1.39E+05	2.94E+05	9.79E+04	0.667	1.86E-22	21.731	-0.774	3.12E-28	27.506
Cer[NDS] 41:0	2.10E+04	6.63E+03	3.03E+04	8.43E+03	1.26E+04	4.59E+03	0.532	1.61E-16	15.792	-1.271	2.49E-48	47.604
Cer[NDS] 42:0	5.53E+04	1.80E+04	8.59E+04	2.46E+04	8.64E+04	2.94E+04	0.637	1.55E-20	19.809	0.009	6.42E-01	0.193
Cer[NDS] 42:1	3.19E+05	8.69E+04	4.38E+05	1.19E+05	1.19E+05	6.62E+04	0.454	1.85E-14	13.732	-1.877	1.23E-62	61.911
Cer[NS] 32:1	3.63E+04	1.06E+04	3.90E+04	1.14E+04	1.36E+04	6.31E+03	0.102	7.91E-02	1.102	-1.519	1.47E-50	49.833
Cer[NS] 33:1	2.97E+04	6.80E+03	2.79E+04	8.19E+03	3.21E+04	1.24E+04	-0.095	6.88E-02	1.163	0.205	8.26E-03	2.083
Cer[NS] 33:4	3.42E+03	2.51E+03	3.45E+03	2.51E+03	3.07E+03	1.29E+05	0.011	9.38E-01	0.028	-0.166	1.88E-01	0.726
Cer[NS] 34:1	1.59E+04	3.85E+03	2.09E+04	5.73E+03	9.68E+03	8.34E+03	0.398	1.56E-12	11.806	-1.112	2.90E-21	20.538
Cer[NS] 34:2	2.34E+04	6.03E+03	2.49E+04	7.48E+03	1.75E+04	9.27E+03	0.088	1.18E-01	0.928	-0.508	3.82E-10	9.418
Cer[NS] 35:1	1.53E+07	1.62E+06	1.37E+07	1.52E+06	1.83E+07	3.43E+06	-0.161	2.35E-12	11.630	0.425	1.51E-25	24.822
Cer[NS] 36:1	1.34E+05	3.17E+04	2.18E+05	6.55E+04	9.40E+04	3.96E+04	0.705	2.21E-25	24.655	-1.215	1.64E-40	39.785
Cer[NS] 36:2	2.02E+04	5.61E+03	2.62E+04	9.87E+03	2.05E+04	8.98E+03	0.375	1.45E-07	6.839	-0.358	1.61E-05	4.793
Cer[NS] 37:1	6.74E+04	1.52E+04	8.99E+04	3.30E+04	5.31E+04	1.39E+04	0.416	1.11E-09	8.956	-0.759	4.32E-22	21.364
Cer[NS] 37:4	1.80E+04	8.47E+03	1.51E+04	6.43E+03	6.05E+04	2.32E+04	-0.258	4.64E-03	2.334	2.005	7.52E-48	47.124
Cer[NS] 38:1	2.14E+05	5.79E+04	3.19E+05	8.17E+04	7.21E+04	4.33E+04	0.576	6.52E-22	21.186	-2.146	5.37E-72	71.270
Cer[NS] 38:2	2.88E+04	7.67E+03	4.12E+04	1.20E+04	1.90E+04	1.04E+04	0.514	1.77E-16	15.751	-1.114	7.88E-32	31.104
Cer[NS] 39:1	2.70E+05	7.50E+04	3.53E+05	9.12E+04	6.63E+04	3.65E+04	0.386	8.94E-12	11.049	-2.412	2.82E-78	77.550
Cer[NS] 39:2	3.59E+04	1.12E+04	4.50E+04	1.32E+04	1.40E+04	7.30E+03	0.326	1.88E-07	6.727	-1.684	4.55E-53	52.342
Cer[NS] 40:1	1.24E+06	3.51E+05	1.76E+06	4.98E+05	3.35E+05	2.32E+05	0.508	4.65E-16	15.333	-2.393	8.99E-70	69.046
Cer[NS] 40:2	2.29E+05	6.42E+04	2.95E+05	7.92E+04	9.43E+04	3.99E+04	0.365	2.34E-10	9.630	-1.646	7.45E-60	59.128
Cer[NS] 41:1	8.06E+04	2.31E+04	1.06E+05	2.94E+04	1.46E+04	1.11E+04	0.401	1.66E-11	10.779	-2.869	2.50E-78	77.602
Cer[NS] 41:2	4.06E+05	1.04E+05	4.99E+05	1.31E+05	1.19E+05	5.51E+04	0.298	3.08E-08	7.512	-2.063	4.53E-72	71.344
Cer[NS] 41:3	5.03E+04	1.54E+04	6.46E+04	2.18E+04	2.51E+04	1.15E+04	0.362	9.47E-08	7.024	-1.367	7.07E-41	40.151
Cer[NS] 42:1	3.19E+05	8.69E+04	4.38E+05	1.19E+05	1.19E+05	6.87E+04	0.454	1.85E-14	13.732	-1.877	5.72E-61	60.242
Cer[NS] 42:2	1.43E+06	3.51E+05	1.95E+06	4.83E+05	5.64E+05	2.43E+05	0.441	4.24E-16	15.373	-1.786	4.30E-69	68.367
Cer[NS] 42:3	8.38E+03	2.99E+03	1.10E+04	4.27E+03	5.71E+03	6.93E+03	0.389	6.82E-07	6.166	-0.943	5.09E-08	7.293
Cer[NS] 43:1	6.74E+05	2.14E+05	6.77E+05	2.27E+05	2.13E+05	9.93E+04	0.006	9.28E-01	0.032	-1.664	1.25E-48	47.902
Cer[NS] 43:2	1.56E+04	5.20E+03	1.63E+04	5.83E+03	5.95E+03	4.37E+03	0.065	3.48E-01	0.458	-1.452	1.39E-31	30.856
Cer[NS] 43:3	7.39E+03	2.98E+03	1.02E+04	3.55E+03	6.00E+03	2.43E+03	0.459	3.96E-09	8.402	-0.760	5.16E-19	18.287
Cer[NS] 44:1	2.63E+04	5.83E+03	2.62E+04	7.21E+03	2.15E+04	7.92E+03	-0.009	8.51E-01	0.070	-0.285	4.68E-04	3.330
Cer[AS] 41:1	2.26E+04	9.70E+03	3.75E+04	1.71E+04	8.72E+03	3.36E+05	0.726	3.98E-13	12.401	-2.102	6.87E-01	0.163
Cer[AS] 42:2	8.56E+04	2.81E+04	9.28E+04	3.52E+04	2.13E+04	1.22E+04	0.116	1.02E-01	0.990	-2.124	6.64E-50	49.178
Cer[EODS] 49:1	4.93E+03	2.44E+03	8.42E+03	3.86E+03	3.20E+03	1.89E+03	0.772	1.87E-13	12.727	-1.398	8.10E-27	26.091
Cer[EODS] 53:0	1.03E+04	3.45E+03	7.86E+03	2.90E+03	1.28E+04	3.81E+03	-0.386	1.06E-07	6.976	0.706	1.27E-21	20.895
Cer[EODS] 57:2	9.93E+03	3.94E+03	9.66E+03	4.45E+03	3.63E+03	4.43E+03	-0.040	6.41E-01	0.193	-1.413	3.88E-17	16.411
Cer[NP] 34:0	5.17E+03	2.63E+03	4.12E+03	1.99E+03	2.44E+03	7.06E+03	-0.328	1.18E-03	2.930	-0.757	2.38E-01	0.623
Cer[NP] 40:0	6.95E+04	2.58E+04	7.25E+04	2.53E+04	1.95E+04	1.31E+04	0.061	3.94E-01	0.404	-1.898	4.11E-47	46.386
Cer[NP] 42:0	1.12E+05	3.24E+04	1.07E+05	3.07E+04	2.96E+04	1.43E+04	-0.062	2.78E-01	0.557	-1.856	3.00E-61	60.522
Cer[NP] 42:1	6.83E+04	2.58E+04	6.41E+04	2.34E+04	2.52E+04	1.06E+04	-0.092	2.14E-01	0.670	-1.345	3.29E-37	36.483
GlcCer[NS] 32:1	3.15E+03	1.60E+03	3.77E+03	1.69E+03	1.39E+03	1.08E+03	0.256	7.17E-03	2.145	-1.441	1.48E-25	24.828
GlcCer[NS] 34:1	1.43E+05	3.15E+04	1.92E+05	5.62E+04	6.05E+04	2.50E+04	0.426	2.00E-13	12.699	-1.669	2.19E-56	55.660
GlcCer[NS] 38:1	8.90E+03	4.03E+03	1.19E+04	3.71E+03	5.24E+03	2.23E+03	0.418	5.68E-08	7.245	-1.183	8.76E-38	37.057
GlcCer[NS] 40:1	1.90E+05	5.23E+04	2.47E+05	7.97E+04	5.42E+04	2.52E+04	0.380	3.48E-09	8.459	-2.188	1.06E-61	60.974
GlcCer[NS] 41:1	1.56E+04	4.32E+03	1.84E+04	5.62E+03	3.91E+03	2.17E+03	0.237	7.59E-05	4.120	-2.233	1.29E-63	62.889
GlcCer[NS] 41:2	8.91E+03	4.44E+03	1.15E+04	5.62E+03	4.07E+03	2.70E+03	0.368	2.55E-04	3.593	-1.500	3.08E-26	25.512
GlcCer[NS] 42:1	5.01E+04	1.29E+04	6.22E+04	1.56E+04	3.38E+04	1.02E+04	0.310	4.44E-09	8.352	-0.878	9.08E-37	36.042
GlcCer[NS] 42:2	3.98E+04	8.63E+03	4.67E+04	1.24E+04	2.55E+04	8.61E+03	0.231	4.40E-06	5.357	-0.873	1.52E-34	33.819
GlcCer[NS] 43:1	6.97E+03	4.72E+03	7.32E+03	3.69E+03	3.84E+03	3.30E+03	0.071	5.46E-01	0.263	-0.930	5.93E-10	9.227
MG 26:4	2.53E+04	7.84E+03	2.82E+04	9.60E+03	1.77E+04	6.37E+03	0.157	1.63E-02	1.786	-0.667	4.52E-17	16.345

DG 30:0	9.81E+03	1.07E+04	1.92E+04	1.69E+04	3.70E+03	3.23E+03	0.969	2.57E-06	5.591	-2.377	4.07E-17	16.391
DG 30:1	5.04E+03	4.75E+03	7.92E+03	6.32E+03	1.75E+03	2.67E+03	0.651	2.36E-04	3.628	-2.180	5.67E-16	15.246
DG 31:0	2.56E+03	2.31E+03	4.10E+03	2.47E+03	1.82E+03	1.18E+03	0.680	4.77E-06	5.321	-1.174	2.52E-15	14.599
DG 32:0	2.57E+04	2.42E+04	7.25E+04	5.16E+04	1.46E+04	1.99E+04	1.496	4.77E-15	14.321	-2.310	1.06E-20	19.976
DG 32:1	3.57E+04	2.92E+04	8.35E+04	5.58E+04	1.70E+04	1.68E+05	1.225	2.70E-13	12.569	-2.299	1.13E-02	1.948
DG 32:2	1.56E+04	1.04E+04	2.62E+04	1.35E+04	5.03E+03	1.01E+05	0.746	1.10E-09	8.959	-2.378	4.26E-01	0.371
DG 33:0	3.24E+03	2.15E+03	5.29E+03	2.92E+03	2.17E+03	1.11E+03	0.709	2.10E-08	7.677	-1.285	1.01E-19	18.997
DG 33:1	6.15E+03	4.88E+03	1.15E+04	6.45E+03	2.67E+03	2.37E+03	0.901	1.13E-10	9.946	-2.105	1.44E-28	27.842
DG 34:0	2.57E+04	1.47E+04	4.58E+04	2.29E+04	1.85E+04	9.80E+03	0.835	8.80E-13	12.056	-1.304	8.87E-23	22.052
DG 34:2	1.39E+05	7.30E+04	3.33E+05	1.56E+05	6.04E+04	4.52E+04	1.257	2.19E-24	23.660	-2.464	7.13E-43	42.147
DG 34:3	3.50E+04	1.82E+04	6.81E+04	2.90E+04	1.58E+04	1.03E+04	0.961	2.10E-19	18.678	-2.112	4.55E-43	42.342
DG 35:1	6.72E+03	3.75E+03	1.18E+04	5.17E+03	2.72E+03	1.59E+03	0.812	2.52E-14	13.599	-2.116	2.66E-42	41.574
DG 35:2	7.43E+03	3.41E+03	1.36E+04	6.14E+03	2.82E+03	3.33E+05	0.869	1.45E-16	15.840	-2.268	2.85E-01	0.545
DG 35:3	4.03E+03	2.12E+03	6.92E+03	3.10E+03	1.53E+03	1.15E+03	0.780	1.28E-13	12.894	-2.174	4.91E-41	40.309
DG 36:0	1.17E+04	3.30E+03	1.31E+04	3.47E+03	1.13E+04	1.60E+04	0.167	2.38E-03	2.623	-0.218	9.04E-01	0.044
DG 36:1	4.31E+04	2.17E+04	8.75E+04	4.14E+04	1.99E+04	1.55E+04	1.023	6.93E-19	18.159	-2.138	1.38E-37	36.861
DG 36:2	2.14E+05	8.10E+04	4.33E+05	2.02E+05	7.17E+04	5.57E+04	1.015	1.61E-20	19.792	-2.595	3.29E-44	43.483
DG 36:3	2.49E+05	9.74E+04	4.97E+05	2.38E+05	6.35E+04	4.86E+04	0.996	2.80E-19	18.553	-2.969	3.37E-46	45.472
DG 36:4	1.65E+05	7.44E+04	2.68E+05	1.15E+05	4.58E+04	3.29E+04	0.704	2.90E-13	12.538	-2.550	1.13E-48	47.946
DG 36:5	1.99E+04	1.04E+04	2.72E+04	1.26E+04	7.46E+03	4.70E+03	0.447	8.37E-06	5.077	-1.865	1.45E-35	34.839
DG 38:0	1.91E+03	1.05E+03	2.18E+03	1.29E+03	1.53E+03	3.20E+03	0.192	9.33E-02	1.030	-0.516	4.90E-01	0.309
DG 38:1	8.37E+03	4.37E+03	1.00E+04	5.02E+03	2.73E+03	1.70E+03	0.257	1.24E-02	1.907	-1.871	7.37E-32	31.133
DG 38:2	9.77E+03	3.62E+03	2.18E+04	8.99E+03	3.15E+03	4.41E+03	1.158	4.64E-28	27.334	-2.791	4.74E-47	46.324
DG 38:3	1.33E+04	4.85E+03	2.52E+04	9.48E+03	8.01E+03	6.31E+03	0.918	6.15E-24	23.211	-1.652	8.82E-37	36.054
DG 38:4	2.09E+04	7.51E+03	3.53E+04	1.28E+04	2.04E+04	2.13E+04	0.756	1.68E-19	18.775	-0.792	1.32E-09	8.881
DG 38:5	3.91E+04	2.01E+04	6.20E+04	2.42E+04	2.80E+04	2.04E+05	0.664	1.96E-12	11.708	-1.147	7.22E-01	0.142
DG 38:6	3.47E+04	1.92E+04	5.16E+04	2.16E+04	2.54E+04	1.89E+04	0.572	7.70E-09	8.114	-1.021	3.41E-16	15.467
DG 38:7	4.72E+03	3.52E+03	5.74E+03	4.20E+03	3.65E+03	3.45E+03	0.282	5.64E-02	1.249	-0.652	1.46E-03	2.837
DG 40:7	1.39E+04	8.78E+03	2.38E+04	1.38E+04	1.38E+04	9.38E+03	0.775	2.41E-09	8.619	-0.792	1.88E-08	7.725
DG 40:8	6.18E+03	4.52E+03	9.19E+03	6.13E+03	5.53E+03	3.62E+03	0.572	6.62E-05	4.179	-0.733	8.60E-07	6.066
LysoPC 14:0	3.99E+04	1.99E+04	2.54E+04	1.39E+04	4.28E+04	1.52E+04	-0.652	3.78E-09	8.423	0.751	3.14E-17	16.503
LysoPC 15:0	5.10E+04	1.94E+04	2.80E+04	1.18E+04	2.76E+04	1.06E+04	-0.868	6.30E-21	20.201	-0.019	7.25E-01	0.140
LysoPC 16:0	5.11E+06	1.26E+06	3.79E+06	1.21E+06	3.75E+06	1.18E+06	-0.433	2.58E-13	12.589	-0.015	5.55E-01	0.255
LysoPC 16:1	9.38E+04	4.87E+04	6.13E+04	3.52E+04	2.61E+05	8.48E+04	-0.615	7.06E-08	7.151	2.091	5.46E-56	55.263
LysoPC 17:0	1.71E+06	1.72E+05	1.59E+06	1.36E+05	1.63E+06	2.82E+05	-0.102	1.38E-07	6.861	0.037	6.88E-01	0.162
LysoPC 17:1	2.28E+04	7.89E+03	1.45E+04	5.35E+03	2.55E+04	7.75E+03	-0.654	1.62E-16	15.790	0.813	5.30E-26	25.276
LysoPC 18:0	3.87E+06	1.24E+06	2.50E+06	1.85E+06	1.97E+06	7.39E+05	-0.628	1.43E-09	8.844	-0.343	4.28E-03	2.369
LysoPC 18:1	1.81E+06	5.76E+05	1.09E+06	5.57E+05	1.55E+06	4.69E+05	-0.730	3.46E-17	16.461	0.506	3.83E-09	8.417
LysoPC 18:2	2.12E+06	9.32E+05	8.57E+05	3.15E+05	1.47E+06	4.84E+05	-1.309	1.62E-29	28.791	0.775	1.92E-20	19.716
LysoPC 18:3	2.71E+04	1.22E+04	1.50E+04	6.29E+03	2.69E+04	7.91E+03	-0.851	1.14E-16	15.943	0.839	1.73E-24	23.763
LysoPC 19:0	3.41E+04	8.11E+03	2.59E+04	8.42E+03	2.56E+04	8.37E+03	-0.397	9.64E-12	11.016	-0.016	5.31E-01	0.275
LysoPC 19:1	1.08E+04	3.58E+03	7.28E+03	4.33E+03	7.47E+03	3.02E+03	-0.573	5.56E-10	9.255	0.037	8.20E-01	0.086
LysoPC 20:0	2.18E+04	7.88E+03	1.35E+04	6.94E+03	1.15E+04	3.80E+03	-0.696	2.77E-14	13.558	-0.222	1.10E-02	1.958
LysoPC 20:1	3.83E+04	1.54E+04	2.61E+04	1.76E+04	1.88E+04	6.19E+03	-0.556	1.86E-07	6.730	-0.474	9.33E-05	4.030
LysoPC 20:2	2.99E+04	1.01E+04	1.85E+04	1.04E+04	3.23E+04	1.24E+04	-0.692	5.50E-14	13.260	0.807	4.84E-15	14.316
LysoPC 20:3	2.20E+05	8.09E+04	1.29E+05	6.79E+04	5.36E+05	1.75E+05	-0.771	3.46E-16	15.461	2.059	2.63E-55	54.580
LysoPC 20:4	5.37E+05	2.00E+05	2.78E+05	1.99E+05	1.85E+06	6.44E+05	-0.947	8.73E-18	17.059	2.734	1.55E-60	59.811
LysoPC 20:5	1.28E+05	7.98E+04	7.71E+04	5.79E+04	9.50E+04	5.31E+04	-0.736	2.23E-07	6.652	0.301	3.93E-02	1.406
LysoPC 22:0	9.32E+03	4.06E+03	6.36E+03	3.83E+03	5.09E+03	3.04E+03	-0.552	1.28E-07	6.894	-0.322	4.39E-02	1.358
LysoPC 22:4	1.93E+04	8.41E+03	1.33E+04	9.91E+03	3.78E+04	6.30E+04	-0.544	2.89E-06	5.539	1.509	3.49E-07	6.457
LysoPC 22:5	3.69E+04	1.45E+04	2.24E+04	1.22E+04	5.23E+04	1.99E+04	-0.718	2.18E-13	12.662	1.219	2.81E-28	27.551
LysoPC 22:6	1.06E+05	4.29E+04	6.59E+04	2.97E+04	1.73E+05	5.89E+04	-0.691	9.88E-14	13.005	1.397	1.68E-39	38.774
LysoPC 24:0	1.60E+04	5.61E+03	1.13E+04	4.50E+03	1.35E+04	3.71E+03	-0.504	1.21E-10	9.916	0.253	1.47E-04	3.833
LysoPC 24:1	5.54E+03	2.03E+03	3.98E+03	1.61E+03	6.06E+03	2.06E+05	-0.477	2.75E-09	8.561	0.605	1.52E-01	0.817
LysoPC 24:4	1.71E+03	1.13E+03	1.66E+03	1.05E+03	6.45E+03	2.51E+04	-0.038	7.65E-01	0.116	1.959	8.66E-04	3.063
LysoPC 26:0	4.15E+03	1.86E+03	2.58E+03	1.46E+03	8.25E+03	7.06E+03	-0.682	1.08E-10	9.968	1.675	7.74E-18	17.111
LysoPC 26:1	3.10E+03	1.43E+03	2.29E+03	1.25E+03	5.25E+03	3.94E+03	-0.435	1.91E-05	4.718	1.195	8.98E-16	15.047
LysoPC 26:2	1.97E+03	1.02E+03	1.50E+03	9.88E+02	3.95E+03	1.45E+03	-0.391	8.22E-04	3.085	1.393	1.02E-35	34.991
LysoPC 26:4	3.24E+03	1.33E+03	3.44E+03	1.53E+03	6.39E+03	2.56E+04	0.087	3.10E-01	0.508	0.894	1.02E-02	1.991
LysoPE 16:0	1.55E+05	6.14E+04	1.55E+05	6.77E+04	1.28E+05	4.68E+04	-0.004	9.63E-01	0.016	-0.273	3.38E-04	3.472
LysoPE 18:0	8.39E+04	2.66E+04	8.77E+04	4.12E+04	5.51E+04	1.64E+04	0.064	4.27E-01	0.369	-0.672	2.24E-12	11.650
LysoPE 18:1	1.95E+05	8.73E+04	1.46E+05	6.46E+04	9.01E+04	3.11E+04	-0.424	4.32E-06	5.364	-0.693	2.23E-14	13.652
LysoPE 18:2	2.06E+05	1.18E+05	1.02E+05	4.49E+04	1.04E+05	4.05E+04	-1.011	3.51E-15	14.454	0.017	8.95E-01	0.048
LysoPE 20:3	3.49E+04	1.69E+04	2.02E+04	1.07E+04	5.71E+04	2.20E+05	-0.790	1.06E-12	11.974	1.502	1.98E-03	2.704
LysoPE 20:4	1.67E+05	6.62E+04	8.94E+04	3.36E+04	3.23E+05	1.23E+05	-0.897	1.35E-21	20.870	1.851	1.64E-45	44.785
LysoPE 22:5	4.24E+04	1.93E+04	3.50E+04	1.80E+04	7.44E+04	2.99E+05	-0.279	3.97E-03	2.401	1.090	6.95E-03	2.158
LysoPE 22:6	6.29E+04	2.52E+04	5.04E+04	1.80E+04	1.53E+05	5.94E+04	-0.320	4.62E-05	4.335	1.607	3.66E-44	43.436
LysoPE 24:0	9.07E+03	3.88E+03	7.91E+03	4.07E+03	8.04E+03	4.32E+03	-0.198	3.39E-02	1.469	0.023	4.06E-01	0.392
PA 34:1	9.75E+03	7.24E+03	9.89E+03	6.66E+03	8.59E+03	4.74E+03	0.020	8.89E-01	0.051	-0.203	2.04E-01	0.690
PA 34:2	1.64E+04	5.27E+03	1.35E+04	4.59E+03	8.23E+03	3.30E+03	-0.281	2.84E-05	4.546	-0.715	1.96E-17	16.708
PA 36:4	9.95E+03	3.41E+03	9.76E+03	4.57E+03	7.08E+03	5.22E+03	-0.028	7.30E-01	0.137	-0.463	1.48E-03	2.829
PA 38:6	7.13E+03	4.15E+03	7.91E+03	3.48E+03	4.24E+03	2.24E+03	0.150	1.40E-01	0.854	-0.901	1.12E-15	14.949
PC 22:0	1.59E+03	1.41E+03	1.35E+03	1.31E+03	1.50E+03	9.51E+03	-0.237	1.99E-01	0.701	0.161	1.43E-01	0.844
PC 23:0	1.85E+03	1.21E+03	2.02E+03	1.22E+03	1.68E+03	9.87E+04	0.126	3.13E-01	0.505	-0.268	1.81E-01	0.743
PC 24:0	4.10E+03	4.20E+03	2.87E+03	2.12E+03	1.76E+03	1.54E+04	-0.516	7.52E-03	2.124	-0.705	5.14E-01	0.289
PC 25:1	2.28E+03	3.17E+03	1.50E+03	1.35E+03	1.67E+03	2.23E+05	-0.600	2.16E-02	1.666	0.156	1.55E-01	0.810
PC 26:0	1.28E+04	1.35E+04	9.75E+03	7.57E+03	2.54E+03	1.94E+05	-0.395	4.20E-02	1.377	-1.939	3.97E-01	0.401
PC 26:1	1.73E+03	1.39E+03	1.29E+03	1.05E+03	7.91E+02	6.12E+03	-0.422	1.04E-02	1.984	-0.706	5.80E-01	0.237
PC 28:0	8.03E+04	7.80E+04	9.11E+04	6.28E+04	1.89E+04	1.29E+04	0.182	2.69E-01	0.570	-2.266	2.04E-24	23.691
PC 28:1	8.05E+03	6.91E+03	7.82E+03	5.30E+03	2.17E+03	4.91E+03	-0.043	7.79E-01	0.109	-1.849	1.39E-11	10.857
PC 29:0	2.40E+04	1.60E+04	2.65E+04	1.47E+04	7.26E+03	1.99E+04	0.139	2.49E-01	0.604	-1.865	6.52E-11	10.186
PC 30:0	1.64E+05	7.27E+04	1.90E+05	7.95E+04	1.22E+05	5.60E+04	0.208	1.56E-02	1.806	-0.638	4.11E-12	11.386
PC 30:1	1.25E+05	7.92E+04	1.83E+05	1.18E+05	5.21E+04	2.97E+04	0.547	4.07E-05	4.390	-1.813	5.52E-23	22.258
PC 30:2	1.72E+04	1.46E+04	1.77E+04	1.04E+04	3.17E+03	5.27E+03	0.040	7.81E-01	0.107	-2.485	2.18E-26	25.662
PC 30:3	1.29E+04	9.52E+03	1.32E+04	6.80E+03	4.30E+03	2.48E+03	0.040	7.52E-01	0.124	-1.624	5.97E-28	27.224
PC 31:0	8.26E+04	3.49E+04	7.40E+04	2.69E+04	7.50E+04	1.95E+05	-0.160	4.37E-02	1.360	0.021	1.45E-01	0.840
PC 31:1	4.51E+04	2.32E+04	6.01E+04	2.94E+04	2.45E+04	3.23E+04	0.412	5.75E-05	4.241	-1.293	3.35E-12	11.475
PC 31:2	2.26E+05	8.75E+04	6.02E+05	2.24E+05	7.64E+04	9.43E+04	1.411	1.60E-38	37.796	-2.978	7.38E-58	57.132
PC 32:0	1.68E+06	2.75E+05	1.92E+06	2.82E+05	1.96E+06	5.50E+05	0.195	1.45E-09	8.837	0.033	9.05E-01	0.043
PC 32:1	1.61E+06	6.87E+05	2.54E+06	9.93E+05	1.98E+06	6.41E+05	0.662	9.78E-14	13.010	-0.361	2.97E-07	6.527
PC 32:2	1.36E+06	5.07E+05	1.90E+06	6.89E+05	2.00E+05	1.84E+05	0.481	6.26E-10	9.203	-3.249	7.10E-64	63.149
PC 32:3	4.11E+04	2.06E+04	5.38E+04	2.72E+04	8.91E+03	6.81E+03	0.388	1.71E-04	3.766	-2.594	3.60E-39	38.443
PC 32:4	1.80E+04	9.48E+03	2.19E+04	1.06E+04	1.11E+04	4.37E+03	0.279	5.62E-03	2.250	-0.976	1.94E-18	17.712
PC 33:0	1.12E+05	3.65E+04	1.13E+05	3.21E+04	8.48E+04	3.30E+04	0.010	8.73E-01	0.059	-0.412	1.39E-09	8.858

PC 33:1	8.58E+04	4.12E+04	1.01E+05	3.96E+04	3.06E+04	1.81E+04	0.237	6.33E-03	2.198	-1.726	7.63E-41	40.117
PC 33:2	5.05E+05	1.75E+05	1.01E+06	3.15E+05	8.49E+04	1.56E+05	1.004	1.96E-33	32.708	-3.575	5.42E-71	70.266
PC 33:3	3.83E+04	1.32E+04	4.32E+04	1.43E+04	9.80E+03	6.83E+03	0.174	1.00E-02	1.999	-2.139	1.33E-55	54.875
PC 34:0	4.29E+05	1.14E+05	5.48E+05	2.09E+05	4.21E+05	1.36E+05	0.356	5.40E-07	6.267	-0.382	6.19E-08	7.208
PC 34:1	1.99E+06	1.21E+06	3.89E+06	2.85E+06	8.24E+05	3.95E+05	0.964	1.64E-09	8.784	-2.239	1.05E-22	21.977
PC 34:2	1.05E+07	1.92E+07	8.78E+07	2.18E+08	5.95E+06	1.33E+07	3.068	3.38E-04	3.471	-3.883	1.47E-04	3.834
PC 34:3	3.37E+06	8.83E+05	4.51E+06	1.16E+06	6.66E+05	5.60E+05	0.417	8.90E-14	13.051	-2.759	7.68E-79	78.115
PC 34:4	4.91E+05	2.05E+05	5.28E+05	2.02E+05	7.45E+04	5.15E+04	0.104	1.91E-01	0.720	-2.827	9.13E-58	57.039
PC 34:5	1.11E+04	8.95E+03	9.07E+03	7.99E+03	2.09E+03	4.36E+04	-0.287	8.74E-02	1.058	-2.114	8.24E-01	0.084
PC 35:1	3.78E+04	2.42E+04	3.47E+04	1.01E+04	1.50E+04	2.98E+04	-0.121	2.34E-01	0.631	-1.208	1.88E-07	6.726
PC 35:2	1.06E+05	5.47E+04	1.14E+05	5.57E+04	2.91E+04	2.58E+04	0.099	3.20E-01	0.495	-1.965	1.60E-32	31.795
PC 35:3	2.56E+05	5.62E+04	2.22E+05	4.20E+04	9.25E+04	2.87E+04	-0.209	9.09E-07	6.041	-1.260	5.01E-69	68.301
PC 35:4	3.41E+05	1.00E+05	2.94E+05	8.74E+04	1.18E+05	3.74E+04	-0.212	3.82E-04	3.418	-1.314	8.56E-49	48.068
PC 35:5	2.97E+04	1.89E+04	2.69E+04	1.67E+04	6.42E+03	4.29E+03	-0.143	2.53E-01	0.596	-2.067	3.91E-26	25.408
PC 35:6	3.24E+05	1.32E+05	6.11E+05	1.79E+05	2.33E+05	1.25E+05	0.916	1.15E-29	28.938	-1.389	1.69E-44	43.773
PC 35:7	1.28E+04	6.29E+03	2.64E+04	1.07E+04	5.45E+03	3.16E+05	1.042	3.56E-23	22.449	-2.274	5.08E-01	0.295
PC 36:0	1.38E+05	3.98E+04	1.48E+05	4.18E+04	1.08E+05	3.67E+04	0.101	7.41E-02	1.130	-0.456	3.86E-13	12.414
PC 36:1	3.31E+06	9.70E+05	4.02E+06	1.07E+06	2.18E+06	6.37E+05	0.280	9.13E-07	6.040	-0.884	6.05E-37	36.218
PC 36:2	4.42E+05	7.54E+05	7.46E+05	1.48E+06	1.72E+05	2.01E+06	0.756	6.00E-02	1.222	-2.115	2.20E-01	0.657
PC 36:3	2.90E+06	5.33E+05	3.29E+06	6.56E+05	1.47E+06	3.61E+05	0.179	5.40E-06	5.268	-1.159	5.35E-66	65.271
PC 36:4	3.39E+06	8.01E+05	3.52E+06	7.26E+05	2.38E+06	6.53E+05	0.051	2.43E-01	0.615	-0.561	7.09E-27	26.150
PC 36:5	2.18E+04	6.35E+04	1.34E+04	3.57E+04	9.62E+03	1.62E+04	-0.700	2.38E-01	0.623	-0.478	3.57E-01	0.447
PC 36:6	2.56E+05	1.13E+05	2.71E+05	1.26E+05	5.30E+04	3.25E+04	0.083	3.61E-01	0.443	-2.353	4.61E-42	41.336
PC 36:7	5.00E+04	1.61E+04	4.55E+04	1.24E+04	1.22E+04	6.76E+05	-0.135	2.51E-02	1.600	-1.901	3.66E-01	0.436
PC 37:1	3.78E+04	1.43E+04	3.79E+04	1.22E+04	2.21E+04	9.09E+03	0.003	9.63E-01	0.016	-0.779	9.70E-22	21.013
PC 37:2	5.18E+04	3.34E+04	5.13E+04	3.66E+04	3.33E+04	2.46E+04	-0.013	9.21E-01	0.036	-0.626	1.04E-04	3.984
PC 37:3	7.08E+04	3.42E+04	6.88E+04	3.05E+04	4.35E+04	2.17E+04	-0.042	6.49E-01	0.188	-0.663	1.56E-11	10.807
PC 37:4	6.32E+05	2.21E+05	4.73E+05	1.74E+05	3.55E+05	1.47E+05	-0.419	2.11E-08	7.676	-0.412	8.78E-08	7.057
PC 37:5	9.04E+04	3.49E+04	1.79E+05	5.53E+04	3.94E+04	5.17E+04	0.987	7.36E-32	31.133	-2.188	4.50E-46	45.347
PC 37:6	1.70E+05	6.28E+04	1.49E+05	5.64E+04	4.81E+04	1.63E+04	-0.195	9.11E-03	2.040	-1.630	1.25E-43	42.903
PC 37:7	5.07E+04	2.20E+04	8.81E+04	2.84E+04	2.73E+04	1.54E+04	0.798	1.04E-21	20.981	-1.690	2.90E-49	48.538
PC 38:1	5.42E+04	1.49E+04	5.84E+04	1.49E+04	2.87E+04	2.76E+04	0.107	4.19E-02	1.378	-1.024	8.01E-16	15.096
PC 38:2	1.48E+05	3.85E+04	1.51E+05	4.51E+04	1.28E+05	1.75E+05	0.036	5.19E-01	0.285	-0.241	9.65E-01	0.015
PC 38:3	1.18E+06	3.13E+05	1.10E+06	2.54E+05	1.27E+06	2.81E+05	-0.100	4.49E-02	1.348	0.212	5.09E-05	4.293
PC 38:4	1.21E+07	2.62E+06	9.57E+06	2.38E+06	1.57E+07	3.50E+06	-0.337	4.83E-12	11.316	0.714	9.46E-33	32.024
PC 38:5	8.91E+05	2.14E+05	8.46E+05	2.05E+05	4.86E+05	1.48E+05	-0.075	1.20E-01	0.922	-0.799	4.43E-35	34.354
PC 38:6	1.18E+07	3.38E+06	1.20E+07	3.04E+06	9.43E+06	2.73E+06	0.023	6.70E-01	0.174	-0.348	7.92E-11	10.101
PC 38:7	1.41E+05	5.15E+04	1.65E+05	5.24E+04	4.74E+04	2.90E+04	0.227	8.81E-04	3.055	-1.803	7.84E-52	51.106
PC 39:3	3.37E+04	1.17E+04	3.03E+04	1.10E+04	3.12E+04	1.22E+05	-0.154	2.90E-02	1.538	0.041	1.46E-01	0.836
PC 39:4	9.13E+04	2.26E+04	7.48E+04	2.36E+04	8.30E+04	3.55E+04	-0.288	4.64E-07	6.334	0.151	7.37E-02	1.132
PC 39:5	3.76E+03	4.20E+03	3.90E+03	5.10E+03	2.50E+03	9.98E+03	0.054	8.22E-01	0.085	-0.642	9.05E-01	0.043
PC 39:6	2.06E+05	7.64E+04	1.49E+05	5.71E+04	1.11E+05	4.10E+04	-0.469	3.25E-09	8.488	-0.421	2.41E-07	6.618
PC 39:7	2.12E+04	8.48E+03	1.81E+04	6.38E+03	1.34E+04	5.88E+04	-0.228	2.93E-03	2.534	-0.438	5.89E-01	0.230
PC 40:0	3.98E+03	2.10E+03	4.83E+03	3.48E+03	2.70E+03	2.29E+06	0.281	3.15E-02	1.502	-0.839	1.62E-01	0.790
PC 40:1	1.20E+04	6.27E+03	1.32E+04	6.40E+03	7.86E+03	1.02E+04	0.139	1.67E-01	0.777	-0.744	4.42E-04	3.355
PC 40:2	3.82E+04	2.49E+04	4.37E+04	2.78E+04	2.05E+04	1.56E+04	0.193	1.32E-01	0.878	-1.089	5.89E-12	11.230
PC 40:3	6.18E+04	2.86E+04	6.68E+04	2.66E+04	4.92E+04	2.40E+05	0.112	1.89E-01	0.724	-0.440	5.11E-01	0.292
PC 40:4	1.49E+05	5.06E+04	1.46E+05	3.56E+04	1.26E+05	3.94E+04	-0.032	5.90E-01	0.229	-0.214	3.15E-05	4.502
PC 40:5	2.61E+05	7.93E+04	2.57E+05	6.99E+04	2.07E+05	7.56E+04	-0.023	6.88E-01	0.163	-0.311	3.17E-07	6.498
PC 40:6	2.67E+06	7.44E+05	2.39E+06	6.05E+05	2.86E+06	9.08E+05	-0.160	3.02E-03	2.520	0.263	4.05E-05	4.392
PC 40:7	9.51E+04	2.64E+04	8.81E+04	2.03E+04	4.43E+04	1.10E+04	-0.112	2.98E-02	1.526	-0.990	2.56E-49	48.592
PC 40:8	2.32E+05	5.99E+04	2.05E+05	4.74E+04	1.38E+05	3.91E+04	-0.179	3.22E-04	3.492	-0.571	5.48E-23	22.261
PC 40:9	1.44E+04	6.30E+03	1.38E+04	6.02E+03	7.63E+03	4.96E+03	-0.067	4.39E-01	0.358	-0.852	1.21E-13	12.917
PC 40:10	3.06E+04	8.89E+03	2.50E+04	7.74E+03	1.70E+04	1.05E+05	-0.291	1.97E-06	5.705	-0.560	5.62E-01	0.250
PC 41:5	1.19E+04	9.14E+03	1.15E+04	1.03E+04	8.14E+03	5.80E+03	-0.038	8.19E-01	0.087	-0.505	6.32E-03	2.200
PC 41:6	1.87E+04	8.05E+03	1.55E+04	7.24E+03	1.48E+04	1.23E+05	-0.266	3.08E-03	2.512	-0.068	1.80E-01	0.744
PC 42:1	1.29E+04	1.03E+04	1.51E+04	2.06E+04	7.33E+03	6.90E+03	0.225	3.30E-01	0.481	-1.042	8.76E-04	3.058
PC 42:2	1.81E+04	1.02E+04	1.71E+04	1.05E+04	8.63E+03	6.28E+03	-0.078	5.02E-01	0.299	-0.988	2.18E-10	9.661
PC 42:3	1.85E+04	1.19E+04	1.93E+04	1.32E+04	1.40E+04	1.52E+04	0.059	6.52E-01	0.186	-0.459	6.72E-02	1.173
PC 42:4	1.47E+04	7.62E+03	1.73E+04	1.09E+04	1.54E+04	2.12E+05	0.241	4.11E-02	1.386	-0.167	1.87E-01	0.727
PC 42:5	4.65E+04	1.41E+04	6.18E+04	1.93E+04	3.65E+04	1.69E+05	0.409	3.65E-10	9.438	-0.757	8.85E-01	0.053
PC 42:6	5.08E+04	1.73E+04	5.40E+04	1.62E+04	3.19E+04	5.15E+04	0.088	1.66E-01	0.780	-0.760	3.28E-01	0.484
PC 42:7	4.94E+04	1.86E+04	4.44E+04	1.79E+04	2.36E+04	8.20E+03	-0.154	4.78E-02	1.320	-0.914	2.28E-22	21.642
PC 42:8	2.81E+04	9.27E+03	2.19E+04	6.56E+03	3.00E+04	9.06E+04	-0.360	5.62E-08	7.250	0.456	2.01E-02	1.698
PC 42:9	2.99E+04	1.03E+04	3.26E+04	1.01E+04	2.55E+04	4.64E+04	0.122	6.10E-02	1.215	-0.355	7.76E-01	0.110
PC 42:10	5.13E+04	1.99E+04	7.00E+04	2.78E+04	3.24E+04	4.28E+04	0.448	5.59E-08	7.253	-1.114	5.06E-10	9.296
PC 42:11	1.11E+04	3.96E+03	8.85E+03	3.94E+03	1.02E+04	9.33E+06	-0.320	7.05E-05	4.152	0.197	1.58E-01	0.801
PC 44:2	3.07E+03	1.69E+03	3.58E+03	2.78E+03	1.87E+03	1.84E+04	0.222	1.07E-01	0.971	-0.939	6.58E-01	0.182
PC 44:3	1.62E+03	9.88E+02	1.69E+03	8.60E+02	1.64E+03	2.44E+05	0.057	6.10E-01	0.215	-0.045	1.58E-01	0.802
PC 44:4	1.50E+04	1.01E+04	1.44E+04	9.18E+03	1.71E+04	9.27E+03	-0.062	6.34E-01	0.198	0.248	3.03E-02	1.519
PC 44:5	9.51E+03	7.25E+03	8.86E+03	5.99E+03	9.57E+03	1.54E+04	-0.103	4.73E-01	0.325	0.112	9.46E-02	1.024
PC 44:6	4.52E+03	2.51E+03	4.30E+03	2.24E+03	7.34E+03	2.96E+03	-0.071	5.07E-01	0.295	0.772	3.77E-16	15.424
PC 46:4	1.87E+03	1.66E+03	1.65E+03	9.69E+02	2.60E+03	9.09E+04	-0.179	2.44E-01	0.613	0.653	1.29E-01	0.890
PC 46:5	1.16E+03	6.62E+02	1.22E+03	9.34E+02	1.52E+03	9.10E+04	0.071	5.97E-01	0.224	0.321	1.48E-01	0.830
PE 30:0	1.03E+04	7.85E+03	1.53E+04	9.99E+03	3.56E+03	3.08E+03	0.571	7.44E-05	4.128	-2.101	1.43E-23	22.846
PE 32:0	2.08E+04	1.03E+04	2.95E+04	1.04E+04	2.20E+04	1.45E+04	0.502	5.00E-09	8.301	-0.424	2.99E-05	4.524
PE 32:1	6.62E+04	3.84E+04	1.81E+05	1.18E+05	5.31E+04	1.38E+05	1.446	5.98E-18	17.223	-1.766	2.52E-09	8.599
PE 32:2	1.14E+04	6.31E+03	3.00E+04	1.63E+04	3.98E+03	7.87E+04	1.401	2.23E-22	21.651	-2.911	5.19E-02	1.285
PE 33:0	9.31E+03	3.80E+03	8.46E+03	3.93E+03	1.06E+04	3.23E+04	-0.137	1.13E-01	0.946	0.323	4.21E-02	1.376
PE 33:1	1.50E+04	6.70E+03	2.67E+04	1.08E+04	7.70E+03	4.61E+03	0.830	7.37E-18	17.132	-1.795	4.26E-40	39.371
PE 33:2	1.47E+04	7.15E+03	3.50E+04	1.43E+04	3.32E+03	5.70E+03	1.253	7.23E-29	28.141	-3.397	4.97E-53	52.304
PE 34:0	3.63E+06	3.90E+05	3.39E+06	3.90E+05	3.85E+06	6.61E+05	-0.096	2.07E-05	4.683	0.183	4.14E-07	6.382
PE 34:1	9.46E+04	3.91E+04	2.56E+05	7.33E+04	5.39E+04	3.25E+04	1.439	1.07E-50	49.971	-2.250	5.42E-07	6.266
PE 34:2	1.26E+06	4.28E+05	2.91E+06	8.93E+05	2.85E+05	4.25E+05	1.203	1.10E-41	40.957	-3.350	2.02E-71	70.695
PE 34:3	2.94E+05	1.67E+05	1.07E+06	5.21E+05	5.64E+04	1.37E+05	1.858	1.25E-33	32.904	-4.242	1.36E-48	47.867
PE 34:4	1.00E+04	5.61E+03	1.82E+04	8.32E+03	3.56E+03	4.28E+03	0.859	8.46E-15	14.072	-2.353	4.69E-37	36.329
PE 35:0	1.46E+04	8.57E+03	1.66E+04	1.05E+04	1.67E+04	2.26E+04	0.186	1.30E-01	0.885	0.010	2.44E-01	0.613
PE 35:1	2.49E+04	1.33E+04	3.93E+04	1.94E+04	1.59E+04	1.04E+04	0.660	1.55E-09	8.809	-1.303	3.22E-22	21.492
PE 35:2	8.06E+04	3.14E+04	1.52E+05	5.08E+04	1.70E+04	1.75E+04	0.912	2.23E-26	25.651	-3.156	3.33E-67	66.478
PE 35:3	1.66E+04	7.70E+03	2.70E+04	1.05E+04	4.62E+03	4.85E+03	0.698	2.91E-14	13.536	-2.547	4.34E-49	48.362
PE 35:4	2.79E+04	1.28E+04	5.29E+04	2.21E+04	7.40E+03	1.72E+04	0.921	9.99E-20	19.000	-2.837	3.75E-38	37.426
PE 36:0	6.36E+04	1.58E+04	5.99E+04	1.77E+04	6.19E+04	1.45E+04	-0.086	1.10E-01	0.960	0.048	6.36E-01	0.197
PE 36:1	4.51E+05	1.76E+05	8.77E+05	2.56E+05	1.03E+05	1.57E+05	0.960	2.13E-32	31.672	-3.085	1.09E-68	67.964
PE 36:2	5.05E+05	1.75E+05	1.01E+06	3.15E+05	8.49E+04	1.58E+05	1.004	1.96E-33	32.708	-3.575	1.27E-70	69.895

PE 36:3	1.35E+06	4.76E+05	2.35E+06	6.55E+05	2.91E+05	3.35E+05	0.798	1.05E-27	26.980	-3.013	2.94E-75	74.532
PE 36:4	1.65E+06	5.88E+05	2.99E+06	7.34E+05	8.58E+05	4.50E+05	0.862	3.04E-34	33.517	-1.803	6.13E-67	66.212
PE 36:5	3.39E+05	2.16E+05	7.35E+05	4.43E+05	7.97E+04	1.78E+05	1.114	1.57E-14	13.804	-3.204	2.53E-32	31.598
PE 36:6	1.06E+04	5.98E+03	2.35E+04	1.23E+04	2.89E+03	3.32E+03	1.142	1.50E-18	17.824	-3.023	9.82E-40	39.008
PE 37:2	2.31E+04	9.38E+03	3.48E+04	1.22E+04	8.15E+03	6.56E+03	0.592	2.32E-13	12.634	-2.093	3.70E-50	49.432
PE 37:3	3.04E+04	1.19E+04	4.11E+04	1.44E+04	1.24E+04	1.24E+05	0.435	1.42E-08	7.848	-1.731	3.25E-01	0.489
PE 37:4	1.01E+05	3.20E+04	1.51E+05	4.12E+04	4.13E+04	5.76E+04	0.587	1.87E-19	18.728	-1.874	5.48E-35	34.262
PE 37:5	6.38E+04	1.96E+04	6.41E+04	2.08E+04	4.55E+04	1.19E+05	0.007	9.13E-01	0.039	-0.494	8.40E-01	0.075
PE 37:6	7.32E+04	4.42E+04	1.54E+05	6.70E+04	1.65E+04	2.65E+05	1.076	1.10E-20	19.960	-3.223	1.67E-04	3.778
PE 38:1	3.46E+04	1.67E+04	4.47E+04	1.78E+04	1.49E+04	8.31E+03	0.370	2.92E-05	4.534	-1.591	2.85E-37	36.545
PE 38:2	8.85E+04	2.55E+04	1.20E+05	3.73E+04	3.39E+04	1.95E+04	0.439	1.17E-11	10.932	-1.824	2.26E-53	52.645
PE 38:3	4.86E+05	1.87E+05	7.88E+05	2.79E+05	2.52E+05	1.30E+05	0.697	2.39E-17	16.621	-1.642	5.51E-45	44.259
PE 38:4	5.19E+05	1.36E+05	9.21E+05	2.19E+05	2.70E+05	2.64E+05	0.826	2.37E-38	37.626	-1.770	1.56E-46	45.808
PE 38:5	2.48E+05	7.58E+04	4.42E+05	1.20E+05	1.19E+05	6.05E+04	0.834	4.69E-32	31.328	-1.898	6.56E-65	64.183
PE 38:6	3.24E+05	1.32E+05	6.11E+05	1.79E+05	2.33E+05	1.24E+05	0.916	1.15E-29	28.938	-1.389	1.44E-44	43.841
PE 38:7	6.38E+04	3.21E+04	1.50E+05	6.01E+04	2.32E+04	1.83E+04	1.232	9.52E-29	28.021	-2.691	5.34E-53	52.272
PE 39:6	7.47E+04	3.27E+04	1.02E+05	3.48E+04	2.46E+04	1.17E+04	0.444	2.48E-08	7.606	-2.048	2.72E-55	54.565
PE 40:2	6.70E+03	3.90E+03	6.84E+03	4.46E+03	5.84E+03	1.09E+04	0.029	8.12E-01	0.091	-0.227	7.75E-01	0.111
PE 40:4	1.05E+05	5.53E+04	1.61E+05	7.98E+04	7.32E+04	3.95E+04	0.611	1.60E-08	7.795	-1.133	2.94E-20	19.532
PE 40:5	3.99E+05	1.42E+05	7.35E+05	2.15E+05	1.81E+05	1.09E+05	0.881	5.03E-30	29.299	-2.025	1.77E-61	60.752
PE 40:6	1.31E+06	5.44E+05	2.26E+06	6.80E+05	5.70E+05	2.89E+05	0.785	3.64E-23	22.438	-1.987	4.89E-61	60.310
PE 40:7	3.89E+05	1.78E+05	7.11E+05	2.29E+05	1.19E+05	8.74E+04	0.868	1.08E-23	22.966	-2.580	1.54E-64	63.812
PE 40:8	8.89E+04	3.10E+04	1.41E+05	4.64E+04	5.32E+04	3.51E+05	0.663	3.02E-18	17.520	-1.404	2.31E-01	0.637
PE 42:10	5.86E+03	3.39E+03	7.82E+03	4.25E+03	1.05E+04	1.62E+04	0.418	2.52E-04	3.599	0.422	7.72E-03	2.112
PL-PC 24:0	4.61E+03	1.96E+03	3.41E+03	2.83E+03	4.80E+03	5.29E+04	-0.435	4.08E-04	3.389	0.493	9.24E-02	1.034
PL-PC 26:0	2.59E+03	1.69E+03	2.19E+03	1.64E+03	3.58E+03	2.09E+03	-0.241	8.25E-02	1.083	0.708	1.31E-08	7.884
PL-PC 30:0	1.29E+04	5.65E+03	1.09E+04	5.67E+03	1.57E+04	2.15E+04	-0.240	1.18E-02	1.930	0.526	7.53E-04	3.123
PL-PC 32:0	9.44E+04	2.66E+04	1.03E+05	4.53E+04	1.06E+05	3.54E+04	0.127	9.04E-02	1.044	0.043	9.67E-01	0.014
PL-PC 32:1	3.85E+04	2.76E+04	4.20E+04	2.76E+04	3.45E+04	2.49E+04	0.124	3.63E-01	0.440	-0.284	6.74E-02	1.171
PL-PC 34:0	5.59E+05	1.65E+05	6.26E+05	1.25E+05	3.24E+05	1.09E+05	0.163	9.99E-04	3.001	-0.949	1.34E-47	46.874
PL-PC 34:1	4.77E+05	1.39E+05	4.77E+05	1.65E+05	1.45E+05	8.19E+04	0.000	9.96E-01	0.002	-1.714	2.21E-46	45.655
PL-PC 34:2	1.00E+06	2.84E+05	8.45E+05	2.27E+05	1.79E+05	9.73E+04	-0.247	1.33E-05	4.875	-2.241	5.59E-73	72.252
PL-PC 34:3	2.81E+04	1.03E+04	2.75E+04	9.83E+03	1.42E+04	5.46E+03	-0.034	6.40E-01	0.194	-0.954	1.07E-26	25.972
PL-PC 34:4	3.13E+03	1.72E+03	3.67E+03	2.31E+03	1.27E+03	4.74E+03	0.228	5.72E-02	1.243	-1.525	7.32E-04	3.136
PL-PC 35:0	1.86E+04	4.99E+03	1.99E+04	5.59E+03	9.32E+03	1.91E+04	0.101	6.67E-02	1.176	-1.095	2.67E-05	4.573
PL-PC 35:1	1.06E+04	4.37E+03	1.32E+04	6.17E+03	4.90E+03	4.75E+03	0.324	3.55E-04	3.450	-1.433	1.90E-20	19.722
PL-PC 35:2	5.83E+04	2.22E+04	5.12E+04	1.99E+04	1.13E+04	7.25E+03	-0.186	1.60E-02	1.796	-2.176	5.40E-49	48.268
PL-PC 35:3	1.65E+04	8.36E+03	1.26E+04	5.50E+03	1.08E+04	4.44E+03	-0.395	6.39E-05	4.194	-0.224	1.34E-02	1.872
PL-PC 36:0	7.42E+04	2.12E+04	8.51E+04	2.25E+04	2.64E+04	1.73E+05	0.196	3.92E-04	3.406	-1.686	4.11E-02	1.386
PL-PC 36:1	3.02E+05	8.09E+04	3.14E+05	7.47E+04	8.28E+04	3.91E+04	0.060	2.36E-01	0.628	-1.925	3.86E-74	73.413
PL-PC 36:2	3.17E+05	1.07E+05	2.87E+05	1.01E+05	1.57E+05	8.64E+04	-0.143	3.74E-02	1.427	-0.866	4.89E-20	19.311
PL-PC 36:3	1.68E+06	3.67E+05	1.45E+06	2.81E+05	1.26E+06	2.48E+05	-0.211	7.83E-07	6.106	-0.206	3.31E-08	7.481
PL-PC 36:4	1.14E+06	3.13E+05	9.89E+05	2.07E+05	8.88E+05	2.18E+05	-0.207	4.04E-05	4.394	-0.155	1.34E-04	3.874
PL-PC 36:5	6.84E+04	3.31E+04	6.57E+04	2.99E+04	4.48E+04	2.43E+04	-0.058	5.36E-01	0.271	-0.551	2.13E-08	7.672
PL-PC 36:6	2.18E+04	7.49E+03	2.35E+04	6.92E+03	1.25E+04	4.40E+03	0.105	9.63E-02	1.016	-0.911	5.70E-31	30.244
PL-PC 37:1	1.36E+04	4.48E+03	1.53E+04	4.88E+03	8.32E+03	3.89E+03	0.164	1.13E-02	1.948	-0.877	1.51E-23	22.821
PL-PC 37:2	1.06E+04	4.08E+03	1.12E+04	4.32E+03	4.75E+03	2.73E+03	0.079	3.04E-01	0.517	-1.232	1.05E-28	27.978
PL-PC 37:3	1.35E+04	5.41E+03	1.46E+04	4.60E+03	9.86E+03	5.51E+03	0.106	1.37E-01	0.864	-0.564	9.02E-11	10.045
PL-PC 37:4	8.98E+04	3.32E+04	7.11E+04	2.17E+04	4.50E+04	3.23E+04	-0.337	2.30E-06	5.638	-0.660	1.24E-08	7.906
PL-PC 37:6	1.66E+05	6.23E+04	2.05E+05	6.36E+04	6.56E+04	3.79E+05	0.305	1.07E-05	4.972	-1.641	2.05E-02	1.689
PL-PC 38:3	9.36E+05	2.01E+05	8.78E+05	2.49E+05	6.88E+05	5.15E+05	-0.091	6.66E-02	1.177	-0.352	4.21E-04	3.376
PL-PC 38:4	9.85E+05	2.66E+05	8.94E+05	2.78E+05	5.73E+05	1.50E+05	-0.141	1.50E-02	1.824	-0.642	2.05E-21	20.688
PL-PC 38:5	9.67E+04	2.50E+04	9.21E+04	2.31E+04	5.46E+04	1.37E+04	-0.069	1.74E-01	0.760	-0.755	1.46E-33	32.837
PL-PC 38:6	1.66E+05	6.20E+04	1.57E+05	5.12E+04	1.60E+05	4.18E+04	-0.078	2.67E-01	0.574	0.026	9.39E-01	0.028
PL-PC 39:5	3.04E+05	1.28E+04	2.78E+04	1.32E+04	2.11E+04	2.99E+05	-0.128	1.50E-01	0.823	-0.400	2.35E-01	0.630
PL-PC 40:0	5.27E+04	3.08E+04	6.05E+04	3.15E+04	2.56E+04	2.02E+04	0.199	6.93E-02	1.159	-1.239	1.35E-18	17.868
PL-PC 40:1	8.58E+03	4.71E+03	9.10E+03	3.82E+03	4.63E+03	2.74E+03	0.085	3.78E-01	0.422	-0.974	3.81E-19	18.419
PL-PC 40:2	1.99E+04	5.70E+03	2.16E+04	5.70E+03	9.83E+03	1.83E+04	0.117	3.20E-02	1.494	-1.137	4.56E-07	6.341
PL-PC 40:3	5.85E+04	2.34E+04	6.17E+04	2.61E+04	2.73E+04	9.53E+03	0.077	3.49E-01	0.458	-1.176	3.37E-28	27.473
PL-PC 40:4	3.11E+05	1.09E+05	3.39E+05	9.67E+04	1.58E+05	5.37E+04	0.124	5.05E-02	1.297	-1.098	2.58E-40	39.589
PL-PC 40:5	2.35E+05	6.80E+04	2.72E+05	8.40E+04	1.68E+05	4.55E+04	0.214	4.45E-04	3.351	-0.699	2.32E-23	22.635
PL-PC 40:6	1.98E+05	6.30E+04	2.00E+05	6.24E+04	1.05E+05	3.20E+04	0.011	8.66E-01	0.062	-0.930	2.05E-32	31.687
PL-PC 41:4	2.70E+04	1.99E+04	3.89E+04	2.04E+04	2.52E+04	2.70E+05	0.528	2.53E-05	4.597	-0.626	3.77E-01	0.424
PL-PC 42:0	2.55E+04	1.55E+04	2.69E+04	2.25E+04	1.20E+04	9.58E+03	0.081	5.79E-01	0.237	-1.169	1.29E-09	8.891
PL-PC 42:1	2.67E+04	1.36E+04	2.45E+04	1.55E+04	1.34E+04	1.40E+04	-0.122	2.80E-01	0.553	-0.870	9.37E-07	6.028
PL-PC 42:2	1.52E+05	1.07E+05	1.67E+05	1.15E+05	7.54E+04	6.54E+04	0.138	3.16E-01	0.500	-1.149	1.19E-11	10.924
PL-PC 42:3	6.60E+04	2.58E+04	6.71E+04	2.47E+04	3.21E+04	3.59E+04	0.023	7.61E-01	0.119	-1.062	2.30E-12	11.638
PL-PC 42:4	2.80E+05	1.74E+05	3.47E+05	1.96E+05	1.45E+05	1.01E+05	0.311	8.83E-03	2.054	-1.259	1.51E-17	16.821
PL-PC 42:5	1.40E+05	5.04E+04	1.30E+05	4.43E+04	5.56E+04	2.46E+04	-0.113	1.06E-01	0.974	-1.223	9.34E-36	35.030
PL-PC 43:3	1.08E+04	6.39E+03	1.11E+04	5.70E+03	5.29E+03	8.46E+06	0.043	6.94E-01	0.159	-1.071	1.61E-01	0.794
PL-PC 43:4	1.10E+04	4.90E+03	1.01E+04	5.17E+03	7.55E+03	4.04E+03	-0.116	2.22E-01	0.654	-0.422	3.40E-04	3.468
PL-PC 44:1	9.38E+03	8.70E+03	8.72E+03	1.05E+04	2.90E+03	2.72E+03	-0.105	6.19E-01	0.209	-1.587	2.14E-07	6.669
PL-PC 44:2	4.36E+04	2.28E+04	4.52E+04	2.66E+04	1.94E+04	1.49E+04	0.053	6.35E-01	0.197	-1.223	4.10E-16	15.387
PL-PC 44:3	9.00E+04	4.30E+04	8.82E+04	5.36E+04	4.34E+04	2.76E+04	-0.029	7.92E-01	0.101	-1.024	3.79E-13	12.421
PL-PC 44:4	3.09E+05	1.53E+05	3.20E+05	1.40E+05	1.59E+05	8.36E+04	0.053	5.65E-01	0.248	-1.014	1.15E-20	19.940
PL-PC 44:5	2.73E+05	8.85E+04	2.54E+05	8.36E+04	1.22E+05	5.60E+04	-0.104	1.11E-01	0.956	-1.062	1.79E-31	30.746
PL-PC 44:6	1.04E+05	4.49E+04	1.06E+05	3.74E+04	6.05E+04	3.39E+05	0.022	7.76E-01	0.110	-0.805	9.82E-01	0.008
PL-PC 46:4	5.62E+04	3.48E+04	5.81E+04	3.52E+04	3.98E+04	2.09E+04	0.049	6.88E-01	0.162	-0.546	5.54E-06	5.256
PL-PE 32:0	2.47E+04	7.51E+03	2.91E+04	9.89E+03	1.67E+04	1.42E+04	0.238	3.15E-04	3.502	-0.806	5.81E-10	9.236
PL-PE 32:1	1.57E+04	5.19E+03	1.66E+04	5.13E+03	1.35E+04	2.51E+05	0.081	2.00E-01	0.700	-0.295	2.02E-01	0.694
PL-PE 32:2	9.58E+03	4.46E+03	1.23E+04	8.09E+03	2.56E+03	2.88E+03	0.357	3.07E-03	2.513	-2.258	9.24E-24	23.034
PL-PE 34:1	2.73E+05	7.03E+04	3.02E+05	7.28E+04	2.18E+05	1.00E+05	0.142	4.37E-03	2.360	-0.472	1.50E-11	10.823
PL-PE 34:2	5.78E+05	1.58E+05	6.67E+05	2.08E+05	9.27E+04	8.18E+04	0.207	5.42E-04	3.266	-2.848	6.30E-69	68.201
PL-PE 34:3	2.41E+04	9.61E+03	2.57E+04	1.18E+04	5.51E+03	7.54E+04	0.095	2.67E-01	0.573	-2.224	1.81E-01	0.742
PL-PE 34:4	2.44E+04	1.08E+04	3.69E+04	5.66E+04	4.45E+03	6.44E+04	0.595	2.67E-02	1.573	-3.053	4.84E-03	2.315
PL-PE 35:1	2.18E+04	9.03E+03	2.56E+04	8.77E+03	9.95E+03	5.57E+03	0.229	2.45E-03	2.611	-1.361	1.07E-36	35.970
PL-PE 35:2	4.89E+04	1.83E+04	4.58E+04	1.76E+04	6.47E+03	8.04E+03	-0.095	2.10E-01	0.679	-2.822	3.51E-52	51.454
PL-PE 35:4	2.20E+04	1.11E+04	2.16E+04	2.90E+04	4.03E+03	1.58E+04	-0.025	9.00E-01	0.046	-2.423	2.55E-06	5.594
PL-PE 36:1	2.26E+05	8.75E+04	2.59E+05	7.82E+04	8.49E+04	5.88E+04	0.196	4.32E-03	2.365	-1.606	1.72E-45	44.764
PL-PE 36:2	7.21E+05	1.98E+05	8.49E+05	2.54E+05	9.47E+04	9.25E+04	0.234	6.75E-05	4.171	-3.164	8.32E-75	74.080
PL-PE 36:3	8.63E+05	2.06E+05	8.46E+05	1.97E+05	2.36E+05	1.10E+05	-0.030	5.28E-01	0.277	-1.844	5.96E-73	72.225
PL-PE 36:4	2.11E+06	6.14E+05	1.94E+06	4.68E+05	9.64E+05	3.96E+05	-0.118	2.84E-02	1.546	-1.009	2.31E-40	39.637
PL-PE 36:5	1.46E+05	1.17E+05	1.21E+05	1.15E+05	2.02E+04	3.00E+04	-0.274	1.14E-01	0.944	-2.580	1.23E-15	14.909
PL-PE 36:6	2.54E+04	1.35E+04	3.67E+04	1.73E+04	1.08E+04	7.27E+03	0.532	2.66E-07	6.576	-1.761	4.43E-32	31.353

PL-PE 37:2	1.57E+04	6.27E+03	1.87E+04	6.50E+03	3.60E+03	6.90E+03	0.250	8.51E-04	3.070	-2.374	2.83E-36	35.548
PL-PE 37:4	1.99E+05	8.29E+04	1.38E+05	4.80E+04	6.28E+04	2.74E+04	-0.528	4.53E-10	9.344	-1.135	1.18E-30	29.927
PL-PE 37:5	4.19E+04	2.41E+04	3.82E+04	2.28E+04	1.36E+04	7.89E+03	-0.133	2.53E-01	0.596	-1.495	2.15E-20	19.668
PL-PE 37:6	2.38E+04	1.08E+04	2.97E+04	1.90E+04	9.74E+03	1.34E+04	0.321	5.77E-03	2.239	-1.608	3.75E-14	13.426
PL-PE 38:1	4.17E+04	3.79E+04	5.05E+04	3.98E+04	1.16E+04	7.03E+03	0.275	1.03E-01	0.988	-2.120	3.07E-19	18.512
PL-PE 38:2	1.15E+05	3.74E+04	1.39E+05	4.80E+04	2.78E+04	2.18E+04	0.275	6.35E-05	4.197	-2.318	1.03E-55	54.986
PL-PE 38:3	5.14E+05	1.60E+05	5.33E+05	1.52E+05	1.67E+05	8.21E+04	0.050	3.96E-01	0.402	-1.677	1.47E-55	54.833
PL-PE 38:4	4.85E+05	1.83E+05	4.49E+05	1.39E+05	1.87E+05	9.04E+04	-0.112	1.07E-01	0.969	-1.265	1.92E-39	38.717
PL-PE 38:5	2.66E+06	6.87E+05	2.34E+06	5.17E+05	8.07E+05	3.15E+05	-0.187	1.50E-04	3.825	-1.534	2.65E-68	67.577
PL-PE 38:6	1.65E+06	4.85E+05	1.87E+06	4.78E+05	8.38E+05	2.98E+05	0.178	1.24E-03	2.906	-1.155	9.01E-48	47.046
PL-PE 39:4	5.15E+04	1.92E+04	4.49E+04	1.50E+04	2.64E+04	2.98E+04	-0.196	6.16E-03	2.211	-0.765	1.10E-05	4.959
PL-PE 39:6	1.09E+05	3.72E+04	1.14E+05	3.42E+04	4.72E+04	1.86E+04	0.070	2.73E-01	0.564	-1.276	6.74E-44	43.171
PL-PE 40:1	1.61E+04	7.83E+03	1.85E+04	8.95E+03	5.86E+03	3.99E+03	0.202	3.73E-02	1.429	-1.663	3.87E-30	29.413
PL-PE 40:2	3.82E+04	1.35E+04	4.55E+04	1.67E+04	1.42E+04	3.75E+04	0.251	5.88E-04	3.230	-1.683	2.72E-10	9.566
PL-PE 40:3	1.26E+05	4.37E+04	1.25E+05	3.12E+04	8.61E+04	2.64E+05	-0.010	8.67E-01	0.062	-0.538	6.99E-01	0.155
PL-PE 40:4	3.87E+05	1.12E+05	3.88E+05	1.02E+05	2.59E+05	1.42E+05	0.000	9.94E-01	0.003	-0.584	3.07E-13	12.512
PL-PE 40:5	5.01E+05	1.45E+05	5.50E+05	1.26E+05	1.92E+05	1.95E+05	0.132	1.03E-02	1.986	-1.518	1.28E-34	33.891
PL-PE 40:6	1.66E+05	6.23E+04	2.05E+05	6.36E+04	6.56E+04	2.55E+04	0.305	1.07E-05	4.972	-1.641	1.40E-53	52.854
PL-PE 42:4	7.49E+04	3.02E+04	7.04E+04	2.35E+04	5.05E+04	1.32E+05	-0.090	2.22E-01	0.654	-0.478	6.89E-01	0.162
PL-PE 42:6	1.05E+05	4.11E+04	1.36E+05	4.77E+04	4.44E+04	1.87E+04	0.372	1.03E-06	5.988	-1.609	7.41E-46	45.130
PG 32:0	8.05E+03	4.14E+03	9.91E+03	6.93E+03	6.54E+03	4.48E+03	0.300	1.84E-02	1.736	-0.601	1.89E-04	3.724
PG 32:1	3.55E+03	1.85E+03	4.70E+03	2.51E+03	2.19E+03	7.65E+03	0.402	2.17E-04	3.664	-1.100	6.01E-02	1.221
PG 33:0	3.48E+04	1.21E+04	3.05E+04	1.13E+04	3.00E+04	2.30E+05	-0.193	7.30E-03	2.136	-0.025	2.48E-01	0.606
PG 34:0	4.72E+06	7.29E+05	3.87E+06	7.75E+05	4.98E+06	1.08E+06	-0.285	2.70E-14	13.568	0.364	1.41E-13	12.852
PG 34:1	4.93E+04	1.49E+04	8.81E+04	2.89E+04	1.71E+04	1.41E+04	0.839	1.50E-26	25.823	-2.369	4.13E-59	58.384
PG 34:2	1.56E+04	5.81E+03	2.08E+04	7.34E+03	4.59E+03	5.74E+03	0.410	5.18E-08	7.286	-2.179	1.67E-42	41.778
PG 36:0	7.34E+04	2.86E+04	5.97E+04	1.23E+04	1.18E+05	4.67E+04	-0.298	9.76E-06	5.011	0.982	8.79E-27	26.056
PG 36:2	9.73E+04	3.14E+04	1.46E+05	5.07E+04	3.45E+04	2.31E+04	0.588	4.57E-15	14.340	-2.083	2.24E-52	51.650
PI 34:1	7.04E+04	3.45E+04	1.23E+05	4.98E+04	2.20E+04	4.14E+04	0.800	3.41E-16	15.467	-2.481	2.64E-36	35.579
PI 34:2	7.86E+04	4.24E+04	1.19E+05	4.30E+04	1.88E+04	1.52E+04	0.599	5.95E-11	10.226	-2.665	1.32E-58	57.881
PI 36:1	7.01E+04	3.39E+04	9.89E+04	4.84E+04	1.62E+04	1.72E+04	0.497	1.07E-06	5.970	-2.610	5.25E-40	39.280
PI 36:2	3.69E+05	1.85E+05	5.12E+05	1.70E+05	4.43E+04	1.27E+05	0.475	1.46E-08	7.836	-3.531	5.50E-57	56.260
PI 36:3	8.82E+04	5.07E+04	1.05E+05	3.70E+04	3.51E+04	1.56E+04	0.247	7.37E-03	2.132	-1.578	1.52E-44	43.819
PI 36:4	8.89E+04	4.16E+04	1.00E+05	3.94E+04	7.91E+04	3.14E+04	0.170	4.76E-02	1.322	-0.339	6.81E-06	5.167
PI 38:3	7.72E+05	4.67E+05	8.35E+05	3.38E+05	3.76E+05	1.72E+05	0.114	2.58E-01	0.588	-1.153	3.39E-28	27.470
PI 38:4	1.10E+06	4.29E+05	1.06E+06	3.54E+05	6.19E+05	2.39E+05	-0.050	4.87E-01	0.312	-0.780	1.32E-22	21.879
PI 38:5	8.44E+04	3.71E+04	8.16E+04	2.97E+04	4.61E+04	2.50E+04	-0.048	5.49E-01	0.260	-0.823	1.71E-16	15.768
PI 38:6	2.00E+04	1.08E+04	2.27E+04	1.14E+04	1.33E+04	2.05E+04	0.182	7.72E-02	1.112	-0.772	2.19E-03	2.659
PI 40:5	9.35E+04	3.82E+04	1.07E+05	3.85E+04	6.11E+04	3.24E+04	0.192	1.24E-02	1.907	-0.807	8.69E-17	16.061
PI 40:6	7.18E+04	3.38E+04	9.38E+04	3.76E+04	3.45E+04	1.41E+04	0.386	1.18E-05	4.929	-1.443	2.76E-36	35.559
PS 36:1	8.23E+04	3.73E+04	1.66E+05	6.08E+04	4.04E+04	3.04E+04	1.015	5.50E-26	25.260	-2.042	4.72E-48	47.326
PS 38:4	1.35E+04	1.94E+04	1.60E+04	9.77E+03	3.21E+04	1.08E+06	0.249	2.30E-01	0.638	1.000	2.15E-01	0.667
SM 29:1	2.44E+03	1.13E+03	2.18E+03	8.45E+02	9.17E+02	7.86E+03	-0.160	6.38E-02	1.196	-1.251	8.11E-01	0.091
SM 30:0	6.26E+03	3.19E+03	7.00E+03	3.28E+03	4.33E+03	5.53E+03	0.161	9.71E-02	1.013	-0.693	1.75E-03	2.756
SM 30:1	6.19E+04	2.08E+04	6.85E+04	2.25E+04	1.73E+04	9.99E+03	0.145	2.87E-02	1.542	-1.982	3.35E-54	53.475
SM 30:2	7.68E+03	3.47E+03	7.05E+03	2.52E+03	3.39E+03	1.80E+03	-0.125	1.28E-01	0.892	-1.055	7.05E-25	24.152
SM 31:1	3.97E+04	1.42E+04	4.17E+04	1.49E+04	1.04E+04	5.88E+03	0.070	3.21E-01	0.494	-1.998	2.48E-50	49.605
SM 32:0	7.04E+04	2.50E+04	9.13E+04	3.70E+04	5.61E+04	3.94E+04	0.375	2.77E-06	5.558	-0.702	5.43E-09	8.265
SM 32:1	1.35E+06	3.67E+05	1.73E+06	4.72E+05	4.26E+05	2.09E+05	0.354	6.63E-10	9.179	-2.022	3.89E-68	67.410
SM 32:2	1.62E+05	4.44E+04	1.78E+05	4.71E+04	7.44E+04	1.94E+05	0.135	1.23E-02	1.909	-1.257	3.32E-05	4.479
SM 33:0	6.21E+04	1.79E+04	7.74E+04	2.32E+04	2.00E+04	5.03E+04	0.318	1.90E-07	6.721	-1.954	2.60E-18	17.584
SM 33:1	4.68E+05	1.39E+05	5.44E+05	1.84E+05	1.81E+05	7.03E+04	0.218	8.01E-04	3.096	-1.590	5.46E-48	47.263
SM 33:2	2.74E+04	8.41E+03	3.18E+04	1.01E+04	9.84E+03	4.65E+03	0.212	8.14E-04	3.089	-1.691	1.80E-50	49.745
SM 34:0	2.63E+05	8.26E+04	3.45E+05	1.04E+05	2.26E+05	1.02E+05	0.393	1.26E-09	8.898	-0.607	2.20E-15	14.658
SM 34:1	2.70E+06	4.07E+05	3.41E+06	7.58E+05	1.64E+06	4.62E+05	0.339	2.38E-15	14.624	-1.061	1.74E-53	52.759
SM 34:2	2.01E+06	4.27E+05	2.67E+06	5.44E+05	1.20E+06	3.82E+05	0.409	6.06E-19	18.217	-1.148	1.60E-59	58.796
SM 34:3	1.41E+04	5.00E+03	1.77E+04	5.72E+03	6.24E+03	6.23E+04	0.323	2.96E-06	5.529	-1.502	5.53E-01	0.257
SM 35:1	1.39E+06	2.22E+05	1.07E+06	2.12E+05	2.33E+06	5.32E+05	-0.376	1.28E-21	20.894	1.119	2.05E-56	55.689
SM 35:2	3.30E+04	1.23E+04	3.56E+04	1.56E+04	1.72E+04	3.24E+04	0.109	1.82E-01	0.741	-1.049	2.30E-05	4.639
SM 35:6	4.31E+03	2.74E+03	5.58E+03	3.29E+03	2.99E+03	2.63E+03	0.372	2.59E-03	2.586	-0.902	6.81E-08	7.167
SM 36:0	5.74E+04	1.85E+04	8.36E+04	2.30E+04	1.32E+05	5.41E+04	0.542	6.36E-17	16.196	0.665	1.86E-14	13.729
SM 36:1	2.20E+06	4.68E+05	2.75E+06	5.96E+05	2.37E+06	5.25E+05	0.327	9.74E-13	12.011	-0.220	4.93E-08	7.308
SM 36:2	1.20E+06	3.09E+05	1.48E+06	4.16E+05	1.70E+06	4.72E+05	0.303	7.36E-08	7.133	0.204	2.42E-03	2.617
SM 36:3	1.58E+05	4.76E+04	2.01E+05	6.01E+04	5.46E+04	3.37E+04	0.342	4.33E-08	7.364	-1.877	5.15E-55	54.288
SM 36:4	3.16E+04	7.64E+03	3.48E+04	8.72E+03	1.81E+04	6.04E+03	0.138	5.25E-03	2.280	-0.943	4.16E-40	39.381
SM 37:1	1.65E+05	7.19E+04	1.84E+05	8.26E+04	1.12E+05	4.65E+04	0.158	7.36E-02	1.133	-0.711	3.10E-13	12.509
SM 37:2	5.11E+04	1.56E+04	4.73E+04	1.49E+04	4.27E+04	1.33E+04	-0.110	7.51E-02	1.124	-0.150	9.15E-03	2.039
SM 38:0	1.63E+05	3.76E+04	2.16E+05	4.94E+04	2.97E+05	9.73E+04	0.403	8.36E-16	15.078	0.460	8.66E-12	11.063
SM 38:1	2.46E+05	1.09E+05	3.24E+05	1.48E+05	2.59E+05	1.25E+05	0.401	1.67E-05	4.778	-0.323	7.71E-04	3.113
SM 38:2	5.86E+05	1.22E+05	5.99E+05	1.54E+05	8.04E+05	1.92E+05	0.030	5.25E-01	0.280	0.425	1.19E-13	12.924
SM 38:3	5.11E+04	1.90E+04	5.13E+04	1.73E+04	4.78E+04	1.27E+06	0.005	9.42E-01	0.026	-0.102	2.69E-01	0.570
SM 38:4	2.02E+04	8.26E+03	2.53E+04	8.87E+03	1.54E+04	7.30E+03	0.326	2.19E-05	4.659	-0.712	1.35E-14	13.869
SM 38:5	1.56E+04	6.44E+03	1.63E+04	7.30E+03	2.72E+04	1.52E+04	0.068	4.29E-01	0.368	0.734	5.47E-12	11.262
SM 39:1	2.20E+05	6.58E+04	2.53E+05	7.11E+04	1.25E+05	1.48E+05	0.200	6.08E-04	3.216	-1.016	6.84E-12	11.165
SM 39:2	1.34E+05	3.08E+04	1.40E+05	3.29E+04	6.65E+04	2.16E+04	0.068	1.44E-01	0.842	-1.075	2.53E-49	48.597
SM 39:3	5.32E+04	1.30E+04	4.76E+04	1.35E+04	4.02E+04	1.15E+04	-0.160	2.48E-03	2.605	-0.244	8.24E-06	5.084
SM 40:0	2.53E+05	6.68E+04	3.10E+05	6.20E+04	3.14E+05	7.62E+04	0.293	8.29E-10	9.081	0.023	9.95E-01	0.002
SM 40:1	1.91E+06	3.93E+05	2.92E+06	4.31E+06	1.16E+06	4.16E+05	0.612	1.71E-02	1.767	-1.334	3.37E-05	4.472
SM 40:2	1.91E+06	1.03E+06	2.06E+06	1.02E+06	1.56E+06	1.25E+06	0.115	2.63E-01	0.581	-0.400	1.03E-03	2.989
SM 40:3	2.43E+05	8.05E+04	2.64E+05	7.22E+04	1.92E+05	5.10E+04	0.117	5.26E-02	1.279	-0.459	5.69E-16	15.245
SM 40:4	3.69E+04	1.02E+04	3.90E+04	1.21E+04	6.54E+04	1.79E+04	0.079	1.81E-01	0.743	0.746	9.76E-27	26.011
SM 40:5	2.17E+04	8.37E+03	3.23E+04	9.63E+03	3.34E+04	1.19E+04	0.573	2.76E-15	14.559	0.049	3.17E-01	0.499
SM 41:0	1.74E+05	4.30E+04	2.18E+05	4.67E+04	8.92E+04	3.68E+04	0.328	1.08E-11	10.968	-1.291	3.52E-58	57.453
SM 41:1	1.28E+06	2.58E+05	1.45E+06	2.61E+05	3.43E+05	1.58E+05	0.176	5.57E-06	5.254	-2.079	3.66E-95	94.437
SM 41:2	7.52E+05	1.19E+05	7.43E+05	1.34E+05	3.09E+05	9.86E+04	-0.017	6.10E-01	0.215	-1.265	1.15E-70	69.939
SM 41:3	1.76E+05	4.03E+04	1.86E+05	4.44E+04	1.18E+05	3.37E+04	0.079	9.13E-02	1.040	-0.660	1.00E-28	27.998
SM 42:1	1.91E+06	4.22E+05	2.26E+06	5.43E+05	1.48E+06	6.11E+05	0.240	4.95E-07	6.305	-0.608	8.14E-20	19.090
SM 42:2	3.79E+06	6.13E+05	1.14E+07	2.91E+07	2.81E+06	9.60E+05	1.593	7.37E-03	2.133	-2.025	2.38E-03	2.623
SM 42:3	2.42E+06	4.58E+05	2.80E+06	6.13E+05	1.96E+06	4.20E+05	0.207	1.07E-06	5.970	-0.509	3.54E-25	24.451
SM 42:4	3.40E+05	8.52E+04	3.68E+05	1.10E+05	5.16E+05	1.32E+05	0.116	3.66E-02	1.436	0.488	1.00E-14	14.000
SM 42:5	8.54E+04	3.69E+04	1.50E+05	7.90E+04	1.58E+05	4.87E+04	0.815	6.95E-13	12.158	0.074	4.99E-01	0.302
SM 42:6	4.89E+04	2.10E+04	7.78E+04	3.32E+04	8.62E+04	3.42E+04	0.668	1.34E-12	11.874	0.148	3.51E-02	1.455
SM 42:7	2.13E+04	9.33E+03	3.02E+04	1.42E+04	2.03E+04	3.80E+05	0.506	1.66E-07	6.779	-0.572	3.56E-01	0.449
SM 43:1	4.34E+04	1.48E+04	4.80E+04	1.69E+04	2.98E+04	1.04E+04	0.145	3.79E-02	1.422	-0.685	5.69E-18	17.245

SM 43:2	1.82E+05	5.42E+04	1.69E+05	5.61E+04	1.04E+05	3.28E+04	-0.105	9.43E-02	1.026	-0.697	9.56E-21	20.020
SM 43:3	6.38E+04	1.73E+04	5.97E+04	2.13E+04	5.39E+04	1.65E+04	-0.095	1.29E-01	0.888	-0.149	1.73E-02	1.761
SM 43:4	1.42E+04	9.96E+03	1.40E+04	1.05E+04	1.87E+04	2.20E+04	-0.024	8.67E-01	0.062	0.414	5.59E-02	1.252
SM 43:7	1.04E+04	4.09E+03	1.18E+04	5.21E+03	7.37E+03	4.16E+03	0.174	3.83E-02	1.417	-0.677	2.14E-10	9.669
SM 44:1	1.36E+04	6.16E+03	1.32E+04	4.71E+03	1.91E+04	3.33E+05	-0.034	6.78E-01	0.169	0.530	1.10E-01	0.959
SM 44:2	9.18E+03	4.58E+03	9.90E+03	5.42E+03	1.20E+04	8.47E+03	0.110	2.95E-01	0.531	0.280	4.68E-03	2.330
SM 44:3	4.04E+04	1.98E+04	3.73E+04	2.23E+04	5.14E+04	2.55E+04	-0.116	2.80E-01	0.553	0.463	4.06E-05	4.391
SM 44:4	1.96E+04	9.00E+03	1.85E+04	9.77E+03	4.40E+04	3.05E+04	-0.082	4.03E-01	0.394	1.253	1.66E-17	16.780
SM 44:5	1.97E+04	7.85E+03	2.90E+04	1.83E+04	4.46E+04	1.73E+04	0.558	2.86E-06	5.544	0.623	5.32E-11	10.274
SM 44:6	1.23E+04	6.37E+03	2.10E+04	1.40E+04	2.69E+04	1.18E+05	0.775	1.79E-08	7.748	0.358	6.13E-02	1.213
SM 44:7	6.45E+03	4.04E+03	7.69E+03	4.55E+03	1.14E+04	5.57E+03	0.254	3.69E-02	1.432	0.566	1.49E-06	5.828
SM 45:4	1.35E+04	7.58E+03	1.38E+04	8.08E+03	7.03E+03	1.72E+04	0.031	7.86E-01	0.105	-0.976	1.34E-02	1.872
SM 47:5	1.15E+04	7.91E+03	1.38E+04	2.00E+04	6.52E+03	6.24E+04	0.260	2.78E-01	0.556	-1.076	9.68E-01	0.014
TG 36:0	7.49E+04	2.07E+05	4.95E+04	9.20E+04	1.63E+04	3.06E+04	-0.598	2.48E-01	0.605	-1.601	5.05E-04	3.297
TG 38:0	1.43E+05	3.36E+05	7.93E+04	1.68E+05	1.82E+04	3.42E+04	-0.854	8.06E-02	1.094	-2.124	4.50E-04	3.347
TG 39:0	2.15E+04	2.68E+04	1.44E+04	1.26E+04	5.23E+03	2.93E+04	-0.575	1.49E-02	1.827	-1.465	4.93E-02	1.307
TG 40:0	2.49E+05	4.71E+05	1.41E+05	2.69E+05	1.41E+04	2.88E+04	-0.822	4.13E-02	1.384	-3.320	3.72E-06	5.429
TG 40:1	1.11E+05	1.69E+05	1.02E+05	1.29E+05	8.62E+03	2.23E+04	-0.123	6.62E-01	0.179	-3.558	6.00E-12	11.222
TG 41:0	3.41E+04	4.68E+04	2.18E+04	2.02E+04	4.83E+03	2.85E+05	-0.641	1.45E-02	1.840	-2.177	5.58E-01	0.253
TG 42:0	3.59E+05	5.33E+05	2.47E+05	3.28E+05	1.80E+04	4.04E+04	-0.542	6.59E-02	1.181	-3.775	1.73E-11	10.763
TG 42:1	3.00E+05	4.12E+05	2.55E+05	3.66E+05	1.55E+04	5.47E+04	-0.237	3.98E-01	0.401	-4.035	5.71E-10	9.244
TG 42:2	1.60E+05	1.94E+05	1.63E+05	2.08E+05	1.02E+04	6.01E+05	0.027	9.15E-01	0.039	-4.000	1.99E-01	0.702
TG 42:3	2.93E+04	2.83E+04	3.90E+04	3.61E+04	4.25E+03	4.06E+03	0.412	3.09E-02	1.510	-3.199	5.01E-19	18.300
TG 43:0	4.56E+04	5.92E+04	3.19E+04	2.48E+04	7.89E+03	8.71E+03	-0.515	2.94E-02	1.531	-2.016	2.17E-17	16.664
TG 44:0	3.53E+05	4.08E+05	3.22E+05	2.88E+05	3.91E+04	6.43E+04	-0.133	5.23E-01	0.282	-3.039	6.94E-19	18.159
TG 44:1	6.00E+05	6.84E+05	5.99E+05	6.06E+05	5.30E+04	8.92E+04	-0.002	9.92E-01	0.003	-3.498	4.13E-17	16.384
TG 44:2	3.44E+05	3.53E+05	3.41E+05	3.15E+05	2.53E+04	4.69E+04	-0.012	9.49E-01	0.023	-3.752	3.29E-20	19.483
TG 45:0	4.99E+04	5.88E+04	3.98E+04	2.60E+04	1.24E+04	9.16E+03	-0.326	1.08E-01	0.967	-1.682	4.79E-20	19.320
TG 45:1	7.03E+04	7.58E+04	7.05E+04	5.02E+04	1.46E+04	1.32E+04	0.006	9.75E-01	0.011	-2.273	1.29E-22	21.890
TG 46:0	4.04E+05	3.57E+05	5.16E+05	3.05E+05	1.12E+05	1.02E+05	0.354	1.48E-02	1.829	-2.203	1.14E-28	27.945
TG 46:1	1.00E+06	9.21E+05	1.30E+06	1.05E+06	2.23E+05	2.10E+05	0.382	2.59E-02	1.586	-2.550	7.03E-21	20.153
TG 46:2	8.30E+05	6.66E+05	1.01E+06	6.95E+05	1.38E+05	1.84E+05	0.283	5.58E-02	1.254	-2.868	1.61E-26	25.792
TG 46:3	3.00E+05	2.28E+05	3.35E+05	2.10E+05	3.72E+04	7.53E+04	0.158	2.50E-01	0.602	-3.172	4.45E-30	29.352
TG 47:0	5.08E+04	5.17E+04	5.17E+04	2.89E+04	2.00E+04	1.80E+04	0.026	8.71E-01	0.060	-1.372	1.91E-16	15.719
TG 47:1	1.15E+05	1.12E+05	1.29E+05	7.43E+04	3.93E+04	2.38E+04	0.160	3.01E-01	0.521	-1.713	5.85E-25	24.233
TG 47:2	1.01E+05	7.45E+04	1.17E+05	5.76E+04	2.82E+04	1.39E+05	0.214	7.81E-02	1.107	-2.058	7.35E-07	6.134
TG 47:3	4.43E+04	2.62E+04	6.13E+04	2.50E+04	1.01E+04	9.73E+03	0.471	2.37E-06	5.625	-2.596	1.83E-48	47.739
TG 48:0	5.08E+05	3.37E+05	8.43E+05	3.43E+05	3.15E+05	1.71E+05	0.731	1.26E-11	10.901	-1.419	6.36E-33	32.196
TG 48:1	2.06E+06	1.33E+06	3.28E+06	1.67E+06	9.31E+05	5.71E+05	0.674	1.30E-08	7.887	-1.817	2.82E-31	30.549
TG 48:2	2.06E+06	1.10E+06	3.05E+06	1.42E+06	7.91E+05	5.16E+05	0.571	3.70E-08	7.432	-1.950	2.13E-36	35.671
TG 48:3	1.18E+06	6.35E+05	1.47E+06	6.83E+05	3.01E+05	2.15E+05	0.309	2.02E-03	2.694	-2.287	6.87E-41	40.163
TG 48:4	4.53E+05	2.90E+05	4.81E+05	2.48E+05	9.12E+04	5.75E+04	0.085	4.60E-01	0.337	-2.399	1.38E-37	36.860
TG 49:0	6.36E+04	4.66E+04	7.75E+04	3.19E+04	3.51E+04	3.75E+04	0.287	1.17E-02	1.933	-1.143	5.76E-14	13.239
TG 49:1	2.49E+05	1.65E+05	3.34E+05	1.34E+05	1.01E+05	4.94E+04	0.426	5.14E-05	4.289	-1.721	4.84E-41	40.315
TG 49:2	2.62E+05	1.32E+05	3.44E+05	1.21E+05	1.07E+05	5.21E+04	0.394	3.97E-06	5.401	-1.683	1.76E-46	45.753
TG 49:3	1.30E+05	5.20E+04	1.71E+05	5.47E+04	4.42E+04	2.49E+04	0.394	7.34E-08	7.134	-1.954	3.45E-56	55.462
TG 50:0	6.10E+05	3.42E+05	1.05E+06	3.43E+05	4.20E+05	2.34E+05	0.789	6.85E-18	17.165	-1.327	1.78E-36	35.750
TG 50:1	3.48E+06	1.57E+06	6.72E+06	1.81E+06	3.12E+06	1.56E+06	0.949	1.17E-31	30.933	-1.537	2.13E-52	51.671
TG 50:2	4.83E+06	1.60E+06	8.03E+06	1.93E+06	1.73E+06	9.45E+05	0.734	3.61E-29	28.443	-1.363	2.20E-59	58.658
TG 50:3	3.52E+06	1.05E+06	5.39E+06	1.36E+06	5.91E+05	3.44E+05	0.616	3.15E-23	22.502	-1.644	1.24E-58	57.906
TG 50:4	1.65E+06	5.48E+05	2.20E+06	6.47E+05	1.30E+05	7.47E+04	0.411	2.95E-10	9.530	-1.896	1.53E-46	45.816
TG 50:5	3.86E+05	1.68E+05	4.72E+05	1.74E+05	2.51E+04	1.88E+04	0.290	3.25E-04	3.488	-1.863	1.85E-38	37.732
TG 50:6	8.33E+04	4.92E+04	9.27E+04	3.78E+04	3.46E+05	1.15E+05	0.154	1.22E-01	0.913	-1.883	6.11E-38	37.214
TG 51:1	2.33E+05	1.29E+05	3.57E+05	1.27E+05	1.18E+05	7.87E+04	0.615	2.53E-11	10.597	-1.605	7.36E-39	38.133
TG 51:2	5.30E+05	1.70E+05	7.12E+05	1.99E+05	2.12E+05	9.52E+04	0.427	1.23E-11	10.909	-1.747	6.56E-61	60.183
TG 51:3	1.31E+04	9.40E+03	1.61E+04	1.12E+04	4.07E+03	7.51E+03	0.301	3.41E-02	1.467	-1.985	1.78E-15	14.751
TG 51:4	2.34E+05	5.56E+04	2.72E+05	6.88E+04	7.14E+04	4.75E+04	0.215	1.94E-05	4.712	-1.927	1.42E-62	61.847
TG 51:5	9.15E+04	3.74E+04	9.64E+04	3.45E+04	3.03E+04	1.56E+04	0.076	3.18E-01	0.497	-1.669	7.90E-45	44.103
TG 52:0	3.18E+05	1.62E+05	4.87E+05	1.62E+05	1.70E+05	9.24E+04	0.616	9.11E-13	12.040	-1.515	1.89E-43	42.724
TG 52:1	2.43E+06	9.93E+05	3.98E+06	1.15E+06	1.32E+06	6.69E+05	0.712	4.49E-21	20.348	-1.591	4.46E-53	52.351
TG 52:2	8.74E+06	1.87E+06	1.29E+07	2.47E+06	4.17E+06	1.86E+06	0.556	6.75E-31	30.171	-1.623	7.68E-76	75.114
TG 52:4	7.73E+06	2.17E+06	1.07E+07	2.66E+06	2.49E+06	1.34E+06	0.465	4.18E-16	15.379	-2.102	1.28E-74	73.893
TG 52:5	2.36E+06	7.60E+05	3.13E+06	9.51E+05	9.07E+05	4.58E+05	0.404	7.17E-10	9.145	-1.786	2.63E-56	55.580
TG 52:6	5.15E+05	2.00E+05	6.16E+05	2.15E+05	2.58E+05	2.01E+05	0.258	5.14E-04	3.289	-1.257	5.41E-25	24.267
TG 53:0	5.42E+04	2.24E+04	5.09E+04	1.68E+04	4.92E+04	1.99E+04	-0.091	2.22E-01	0.654	-0.050	3.65E-01	0.438
TG 53:1	1.00E+05	5.09E+04	1.39E+05	5.36E+04	3.65E+04	1.95E+04	0.470	1.95E-07	6.709	-1.928	2.33E-46	45.632
TG 53:2	3.12E+05	8.98E+04	4.53E+05	1.42E+05	1.19E+05	5.33E+04	0.536	1.73E-15	14.762	-1.932	5.09E-59	58.294
TG 53:3	3.86E+05	8.44E+04	4.32E+05	1.15E+05	1.23E+05	7.00E+04	0.164	9.49E-04	3.023	-1.813	7.49E-61	60.126
TG 53:4	1.78E+04	1.25E+04	2.19E+04	1.27E+04	7.36E+03	5.96E+04	0.304	1.72E-02	1.764	-1.574	1.53E-01	0.816
TG 53:5	1.15E+05	3.08E+04	1.17E+05	2.69E+04	5.23E+04	2.37E+04	0.025	6.12E-01	0.213	-1.164	3.29E-46	45.483
TG 54:0	8.28E+04	4.96E+04	1.07E+05	4.94E+04	3.87E+04	4.38E+04	0.367	5.26E-04	3.279	-1.463	1.25E-19	18.903
TG 54:1	5.81E+05	3.50E+05	8.30E+05	3.51E+05	2.26E+05	1.98E+05	0.513	5.57E-07	6.254	-1.875	8.62E-37	36.064
TG 54:2	1.92E+06	7.31E+05	2.67E+06	8.45E+05	7.82E+05	4.67E+05	0.481	3.88E-11	10.411	-1.774	1.02E-51	50.992
TG 54:3	2.90E+06	9.43E+05	3.16E+06	8.10E+05	1.26E+06	5.93E+05	0.127	2.83E-02	1.548	-1.331	3.02E-50	49.519
TG 54:4	3.76E+06	1.22E+06	3.85E+06	9.54E+05	1.42E+06	2.93E+06	0.035	5.39E-01	0.268	-1.439	3.19E-11	10.496
TG 54:5	3.40E+06	1.21E+06	3.54E+06	1.02E+06	1.53E+06	6.48E+05	0.061	3.39E-01	0.470	-1.216	3.13E-43	42.505
TG 54:6	2.17E+06	9.75E+05	2.37E+06	8.75E+05	1.17E+06	4.94E+05	0.129	1.12E-01	0.951	-1.012	3.67E-27	26.436
TG 54:7	7.75E+05	4.09E+05	8.54E+05	4.07E+05	5.22E+05	2.68E+05	0.140	1.59E-01	0.798	-0.711	1.31E-11	10.883
TG 54:8	1.69E+05	9.03E+04	1.75E+05	9.19E+04	1.20E+05	6.82E+04	0.045	6.69E-01	0.174	-0.539	1.13E-06	5.948
TG 55:1	2.04E+04	1.28E+04	3.17E+04	1.57E+04	7.70E+03	5.69E+03	0.631	4.07E-08	7.391	-2.039	2.14E-34	33.670
TG 55:2	3.92E+04	1.65E+04	5.49E+04	1.89E+04	1.22E+04	1.04E+04	0.486	7.84E-10	9.106	-2.170	9.14E-51	50.039
TG 55:3	5.28E+04	1.53E+04	7.08E+04	2.33E+04	2.12E+04	8.20E+04	0.423	2.53E-10	9.597	-1.740	2.98E-06	5.526
TG 56:0	1.14E+04	1.04E+04	1.73E+04	1.23E+04	4.02E+03	1.70E+05	0.606	1.85E-04	3.733	-2.110	7.98E-01	0.098
TG 56:1	8.09E+04	8.13E+04	1.16E+05	8.56E+04	1.81E+04	5.21E+06	0.524	2.24E-03	2.649	-2.688	2.90E-01	0.538
TG 56:2	1.69E+05	1.32E+05	2.58E+05	1.36E+05	4.67E+04	7.21E+04	0.614	2.32E-06	5.635	-2.465	1.83E-32	31.736
TG 56:3	2.32E+05	1.27E+05	3.33E+05	1.29E+05	8.99E+04	6.23E+04	0.520	3.57E-08	7.448	-1.887	3.24E-43	42.489
TG 56:4	2.62E+05	7.50E+04	3.12E+05	8.00E+04	2.15E+05	9.82E+04	0.249	6.20E-06	5.208	-0.535	1.23E-14	13.911
TG 56:5	5.58E+05	1.38E+05	5.65E+05	1.26E+05	5.46E+05	2.27E+05	0.016	7.32E-01	0.136	-0.048	2.87E-01	0.542
TG 56:6	7.94E+05	2.13E+05	7.81E+05	1.81E+05	8.57E+05	3.27E+05	-0.024	6.32E-01	0.199	0.134	9.21E-02	1.036
TG 56:7	9.08E+05	3.17E+05	1.03E+06	3.58E+05	1.08E+06	4.65E+05	0.175	1.23E-02	1.911	0.081	4.62E-01	0.335
TG 56:8	5.90E+05	2.59E+05	6.79E+05	2.85E+05	6.72E+05	3.72E+05	0.202	1.86E-02	1.731	-0.015	7.20E-01	0.143
TG 56:9	1.70E+05	8.65E+04	1.73E+05	8.41E+04	1.74E+05	1.23E+05	0.024	8.07E-01	0.093	0.009	4.39E-01	0.358
TG 56:10	2.83E+04	2.02E+04	2.65E+04	2.02E+04	2.52E+04	1.38E+04	-0.098	5.03E-01	0.298	-0.070	5.75E-01	0.240
TG 58:1	2.38E+04	3.33E+04	3.49E+04	3.38E+04	5.99E+03	2.32E+04	0.551	1.70E-02	1.770	-2.541	7.48E-12	11.126

TG 58:2	6.69E+04	1.21E+05	9.33E+04	9.77E+04	1.22E+04	7.43E+04	0.479	8.20E-02	1.086	-2.929	2.30E-09	8.637
TG 58:3	5.57E+04	7.91E+04	8.44E+04	7.43E+04	1.30E+04	3.36E+04	0.600	6.96E-03	2.157	-2.700	9.39E-17	16.027
TG 58:4	3.76E+04	2.38E+04	5.88E+04	2.57E+04	1.98E+04	2.90E+04	0.646	2.29E-09	8.639	-1.572	2.07E-18	17.684
TG 58:5	6.87E+04	2.43E+04	1.25E+05	4.30E+04	6.68E+04	5.25E+04	0.868	5.19E-25	24.285	-0.909	4.48E-15	14.348
TG 58:6	1.14E+05	3.56E+04	1.55E+05	4.67E+04	1.31E+05	5.30E+04	0.451	5.62E-12	11.250	-0.245	3.38E-04	3.471
TG 58:7	1.80E+05	6.57E+04	2.08E+05	7.25E+04	2.35E+05	8.22E+04	0.202	4.79E-03	2.319	0.180	1.75E-02	1.757
TG 58:8	2.34E+05	9.61E+04	2.47E+05	1.03E+05	3.27E+05	1.22E+05	0.074	3.67E-01	0.435	0.408	1.39E-06	5.858
TG 58:9	2.12E+05	1.03E+05	2.08E+05	9.06E+04	2.96E+05	1.19E+05	-0.021	8.15E-01	0.089	0.508	2.80E-08	7.552
TG 58:10	1.37E+05	7.90E+04	1.33E+05	6.60E+04	1.98E+05	9.25E+04	-0.049	6.47E-01	0.189	0.579	3.65E-08	7.438
TG 58:11	3.89E+04	2.98E+04	3.72E+04	3.01E+04	4.91E+04	8.27E+04	-0.067	6.68E-01	0.175	0.402	1.39E-02	1.858
TG 60:10	4.46E+04	2.39E+04	4.14E+04	2.12E+04	1.06E+05	4.83E+05	-0.108	3.02E-01	0.519	1.361	6.54E-03	2.185
TG 60:11	4.57E+04	3.07E+04	4.57E+04	2.99E+04	1.23E+05	2.07E+05	0.000	9.98E-01	0.001	1.432	1.41E-06	5.850
TG 60:12	3.50E+04	3.21E+04	3.67E+04	3.47E+04	8.94E+04	6.04E+04	0.072	6.97E-01	0.156	1.284	2.69E-14	13.570
TG 62:1	1.93E+03	4.73E+03	2.33E+03	4.20E+03	3.98E+02	1.29E+04	0.274	5.12E-01	0.291	-2.548	8.30E-01	0.081
TG 62:12	1.03E+04	8.51E+03	1.08E+04	8.37E+03	3.16E+04	1.70E+04	0.075	6.35E-01	0.197	1.548	3.28E-23	22.484
TG 62:14	6.71E+03	7.30E+03	6.43E+03	7.09E+03	2.35E+04	4.47E+05	-0.061	7.78E-01	0.109	1.872	1.15E-01	0.940

Supplementary Table S2. Lipid group classification. Lipidomics dataset was collapsed into groups of lipids based on the lipid class and the number of double bonds within the fatty acid tails. Group names, the number of lipids within each group, and a list of the individual lipids composed in the group are reported.

Lipid Class	Group Name	Saturation	Number of lipids	Lipid Species
acylcarnitine	AC	all	6	Acylcarnitine 16:0, Acylcarnitine 18:0, Acylcarnitine 24:0, Acylcarnitine 26:0, Acylcarnitine 18:1, Acylcarnitine 18:2
cholesteryl ester	CE	saturated, monounsaturated	5	16:0 Cholesteryl ester, 18:0 Cholesteryl ester, 16:1 Cholesteryl ester, 17:1 Cholesteryl ester, 18:1 Cholesteryl ester
	CE-poly	polyunsaturated	8	18:2 Cholesteryl ester, 18:3 Cholesteryl ester, 20:3 Cholesteryl ester, 20:4 Cholesteryl ester, 22:4 Cholesteryl ester, 20:5 Cholesteryl ester, 22:5 Cholesteryl ester, 22:6 Cholesteryl ester
ceramide	CER-sat	saturated	10	Cer[NDS] 34:0, Cer[NDS] 36:0, Cer[NDS] 38:0, Cer[NDS] 40:0, Cer[NDS] 41:0, Cer[NDS] 42:0, Cer[EODS] 53:0, Cer[NP] 34:0, Cer[NP] 40:0, Cer[NP] 42:0,
	CER-mono	monounsaturated	16	Cer[NS] 32:1, Cer[NS] 33:1, Cer[NS] 34:1, Cer[NS] 35:1, Cer[NS] 36:1, Cer[NS] 37:1, Cer[NS] 38:1, Cer[NS] 39:1, Cer[NS] 40:1, Cer[NS] 41:1, Cer[NS] 42:1, Cer[NS] 43:1, Cer[NS] 44:1, Cer[AS] 41:1, Cer[EODS] 49:1, Cer[NP] 42:1
	CER-poly	polyunsaturated	15	Cer[NS] 34:2, Cer[NS] 36:2, Cer[NS] 38:2, Cer[NS] 39:2, Cer[NS] 40:2, Cer[NS] 41:2, Cer[NS] 42:2, Cer[NS] 43:2, Cer[AS] 42:2, Cer[EODS] 57:2, Cer[NS] 41:3, Cer[NS] 42:3, Cer[NS] 43:3, Cer[NS] 33:4, Cer[NS] 37:4
diglyceride	DG-sat	saturated	13	DG 30:0, DG 31:0, DG 32:0, DG 33:0, DG 34:0, DG 36:0, DG 38:0, DG 30:1, DG 32:1, DG 33:1, DG 35:1, DG 36:1, DG 38:1
	DG-mono	monounsaturated	9	DG 32:2, DG 34:2, DG 35:2, DG 36:2, DG 38:2, DG 34:3, DG 35:3, DG 36:3, DG 38:3
	DG-poly	polyunsaturated	8	DG 36:4, DG 38:4, DG 36:5, DG 38:5, DG 38:6, DG 38:7, DG 40:7, DG 40:8
free fatty acid	FFA-sat	saturated	5	FFA18:0, FFA16:0, FFA20:0, FFA22:0, FFA24:0
	FFA-mono	monounsaturated	4	FFA18:1, FFA20:1, FFA22:1, FFA24:1
	FFA-poly	polyunsaturated	7	FFA18:2, FFA20:2, FFA22:2, FFA24:2, FFA22:3, FFA24:3, FFA20:4
glucosylceramidase	GlcCer	all	9	GlcCer[NS] 32:1, GlcCer[NS] 34:1, GlcCer[NS] 38:1, GlcCer[NS] 40:1, GlcCer[NS] 41:1, GlcCer[NS] 42:1, GlcCer[NS] 43:1, GlcCer[NS] 41:2, GlcCer[NS] 42:2
lysophosphatidylcholine	LysoPC-sat	saturated	10	LysoPC 14:0, LysoPC 15:0, LysoPC 16:0, LysoPC 17:0, LysoPC 18:0, LysoPC 19:0, LysoPC 20:0, LysoPC 22:0, LysoPC 24:0, LysoPC 26:0
	LysoPC-mono	monounsaturated	7	LysoPC 16:1, LysoPC 17:1, LysoPC 18:1, LysoPC 19:1, LysoPC 20:1, LysoPC 24:1, LysoPC 26:1
	LysoPC-poly	polyunsaturated	12	LysoPC 18:2, LysoPC 20:2, LysoPC 26:2, LysoPC 18:3, LysoPC 20:3, LysoPC 20:4, LysoPC 22:4, LysoPC 24:4, LysoPC 26:4, LysoPC 20:5, LysoPC 22:5, LysoPC 22:6
lysophosphatidylethanolamine	LysoPE-sat	saturated	3	LysoPE 16:0, LysoPE 18:0, LysoPE 24:0
	LysoPE-poly	polyunsaturated	5	LysoPE 18:2, LysoPE 20:3, LysoPE 20:4, LysoPE 22:5, LysoPE 22:6
phosphatidylcholine	PC-sat	saturated	27	PC 22:0, PC 23:0, PC 24:0, PC 26:0, PC 28:0, PC 29:0, PC 30:0, PC 31:0, PC 32:0, PC 33:0, PC 34:0, PC 36:0, PC 40:0, PC 25:1, PC 26:1, PC 28:1, PC 30:1, PC 31:1, PC 32:1, PC 33:1, PC 34:1, PC 35:1, PC 36:1, PC 37:1, PC 38:1, PC 40:1, PC 42:1
	PC-mono	monounsaturated	24	PC 30:2, PC 31:2, PC 32:2, PC 33:2, PC 34:2, PC 35:2, PC 36:2, PC 37:2, PC 38:2, PC 40:2, PC 42:2, PC 44:2, PC 30:3, PC 32:3, PC 33:3, PC 34:3, PC 35:3, PC 36:3, PC 37:3, PC 38:3, PC 39:3, PC 40:3, PC 42:3, PC 44:3
	PC-poly	polyunsaturated	45	PC 40:10, PC 42:10, PC 42:11, PC 32:4, PC 34:4, PC 35:4, PC 36:4, PC 37:4, PC 38:4, PC 39:4, PC 40:4, PC 42:4, PC 44:4, PC 46:4, PC 34:5, PC 35:5, PC 36:5, PC 37:5, PC 38:5, PC 39:5, PC 40:5, PC 41:5, PC 42:5, PC 44:5, PC 46:5, PC 35:6, PC 36:6, PC 37:6, PC 38:6, PC 39:6, PC 40:6, PC 41:6, PC 42:6, PC 44:6, PC 35:7, PC 36:7, PC 37:7, PC 38:7, PC 39:7, PC 40:7, PC 42:7, PC 40:8, PC 42:8, PC 40:9, PC 42:9
phosphatidylethanolamine	PE-sat	saturated	12	PE 30:0, PE 32:0, PE 33:0, PE 34:0, PE 35:0, PE 36:0, PE 32:1, PE 33:1, PE 34:1, PE 35:1, PE 36:1, PE 38:1
	PE-mono	monounsaturated	13	PE 32:2, PE 33:2, PE 34:2, PE 35:2, PE 36:2, PE 37:2, PE 38:2, PE 40:2, PE 34:3, PE 35:3, PE 36:3, PE 37:3, PE 38:3
	PE-poly	polyunsaturated	19	PE 42:10, PE 34:4, PE 35:4, PE 36:4, PE 37:4, PE 38:4, PE 40:4, PE 36:5, PE 37:5, PE 38:5, PE 40:5, PE 36:6, PE 37:6, PE 38:6, PE 39:6, PE 40:6, PE 38:7, PE 40:7, PE 40:8
phosphatidylglycerol	PG-sat	saturated	6	PG 32:0, PG 33:0, PG 34:0, PG 36:0, PG 32:1, PG 34:1
	PG-mono	monounsaturated	2	PG 34:2, PG 36:2
phosphatidylinositol	PI-sat	saturated	2	PI 34:1, PI 36:1
	PI-mono	monounsaturated	4	PI 34:2, PI 36:2, PI 36:3, PI 38:3
	PI-poly	polyunsaturated	6	PI 36:4, PI 38:4, PI 38:5, PI 40:5, PI 38:6, PI 40:6
Plasmenyl-phosphatidylcholine	PLPC-sat	saturated	17	PL-PC 24:0, PL-PC 26:0, PL-PC 30:0, PL-PC 32:0, PL-PC 34:0, PL-PC 35:0, PL-PC 36:0, PL-PC 40:0, PL-PC 42:0, PL-PC 32:1, PL-PC 34:1, PL-PC 35:1, PL-PC 36:1, PL-PC 37:1, PL-PC 40:1, PL-PC 42:1, PL-PC 44:1
	PLPC-mono	monounsaturated	16	PL-PC 34:2, PL-PC 35:2, PL-PC 36:2, PL-PC 37:2, PL-PC 40:2, PL-PC 42:2, PL-PC 44:2, PL-PC 34:3, PL-PC 35:3, PL-PC 36:3, PL-PC 37:3, PL-PC 38:3, PL-PC 40:3, PL-PC 42:3, PL-PC 43:3, PL-PC 44:3
	PLPC-poly	polyunsaturated	21	PL-PC 34:4, PL-PC 36:4, PL-PC 37:4, PL-PC 38:4, PL-PC 40:4, PL-PC 41:4, PL-PC 42:4, PL-PC 43:4, PL-PC 44:4, PL-PC 46:4, PL-PC 36:5, PL-PC 38:5, PL-PC 39:5, PL-PC 40:5, PL-PC 42:5, PL-PC 44:5, PL-PC 36:6, PL-PC 37:6, PL-PC 38:6, PL-PC 40:6, PL-PC 44:6
Plasmenyl-phosphatidylethanolamine	PLPE-sat	saturated	7	PL-PE 32:0, PL-PE 32:1, PL-PE 34:1, PL-PE 35:1, PL-PE 36:1, PL-PE 38:1, PL-PE 40:1
	PLPE-mono	monounsaturated	11	PL-PE 32:2, PL-PE 34:2, PL-PE 35:2, PL-PE 36:2, PL-PE 37:2, PL-PE 38:2, PL-PE 40:2, PL-PE 34:3, PL-PE 36:3, PL-PE 38:3, PL-PE 40:3
	PLPE-poly	polyunsaturated	18	PL-PE 34:4, PL-PE 35:4, PL-PE 36:4, PL-PE 37:4, PL-PE 38:4, PL-PE 39:4, PL-PE 40:4, PL-PE 42:4, PL-PE 36:5, PL-PE 37:5, PL-PE 38:5, PL-PE 40:5, PL-PE 36:6, PL-PE 37:6, PL-PE 38:6, PL-PE 39:6, PL-PE 40:6, PL-PE 42:6
	SM-sat	saturated	24	SM 30:0, SM 32:0, SM 33:0, SM 34:0, SM 36:0, SM 38:0, SM 40:0, SM 41:0, SM 29:1, SM 30:1, SM 31:1, SM 32:1, SM 33:1, SM 34:1, SM 35:1, SM 36:1, SM 37:1, SM 38:1, SM 39:1, SM 40:1, SM 41:1, SM 42:1, SM 43:1, SM 44:1

sphingomyelin	SM-mono	monounsaturated	23	SM 30:2, SM 32:2, SM 33:2, SM 34:2, SM 35:2, SM 36:2, SM 37:2, SM 38:2, SM 39:2, SM 40:2, SM 41:2, SM 42:2, SM 43:2, SM 44:2, SM 34:3, SM 36:3, SM 38:3, SM 39:3, SM 40:3, SM 41:3, SM 42:3, SM 43:3, SM 44:3
	SM-poly	polyunsaturated	18	SM 36:4, SM 38:4, SM 40:4, SM 42:4, SM 43:4, SM 44:4, SM 45:4, SM 38:5, SM 40:5, SM 42:5, SM 44:5, SM 47:5, SM 35:6, SM 42:6, SM 44:6, SM 42:7, SM 43:7, SM 44:7
triglyceride	TG-sat	saturated	50	TG 36:0, TG 38:0, TG 39:0, TG 40:0, TG 41:0, TG 42:0, TG 43:0, TG 44:0, TG 45:0, TG 46:0, TG 47:0, TG 48:0, TG 49:0, TG 50:0, TG 51:0, TG 52:0, TG 53:0, TG 54:0, TG 56:0, TG 40:1, TG 42:1, TG 44:1, TG 45:1, TG 46:1, TG 47:1, TG 48:1, TG 49:1, TG 50:1, TG 51:1, TG 52:1, TG 53:1, TG 54:1, TG 55:1, TG 56:1, TG 58:1, TG 62:1, TG 42:2, TG 44:2, TG 46:2, TG 47:2, TG 48:2, TG 49:2, TG 50:2, TG 51:2, TG 52:2, TG 53:2, TG 54:2, TG 55:2, TG 56:2, TG 58:2
	TG-mono	monounsaturated	28	TG 42:3, TG 46:3, TG 47:3, TG 48:3, TG 49:3, TG 50:3, TG 51:3, TG 52:3, TG 53:3, TG 54:3, TG 55:3, TG 56:3, TG 58:3, TG 48:4, TG 50:4, TG 51:4, TG 52:4, TG 53:4, TG 54:4, TG 56:4, TG 58:4, TG 50:5, TG 51:5, TG 52:5, TG 53:5, TG 54:5, TG 56:5, TG 58:5
	TG-poly	polyunsaturated	21	TG 50:6, TG 52:6, TG 54:6, TG 56:6, TG 58:6, TG 54:7, TG 56:7, TG 58:7, TG 54:8, TG 56:8, TG 58:8, TG 56:9, TG 58:9, TG 56:10, TG 58:10, TG 60:10, TG 58:11, TG 60:11, TG 60:12, TG 62:12, TG 62:14

Supplementary Table S3. Debiased Sparse Partial Correlation coefficients depicting relationship between lipid clusters within and between M1, M3, and CB. Partial correlation coefficients represented have an FDR adjusted p-value <0.1.

Lipid Group 1	Lipid Group 2	r-value	unadjusted p-value	adjusted p-value
M1 CE	M1 CE-poly	0.459	0.000619	0.0159
M1 CE	M1 PC-sat	0.536	0.000695	0.0172
M1 CE-poly	M1 PC-poly	0.589	0.00027	0.00714
M1 CE-poly	M1 DG-mono	-0.573	0.000713	0.0172
M1 CE-poly	M1 SM-sat	0.538	0.00163	0.0351
M1 CER-mono	M1 CER-poly	1	3.83E-17	7.84E-15
M1 CER-mono	M1 SM-sat	1	1.19E-05	0.000374
M1 CER-sat	M1 CER-mono	1	9.30E-08	4.24E-06
M1 DG-mono	M1 DG-poly	1	1.79E-14	2.09E-12
M1 DG-mono	M1 TG-mono	0.912	1.10E-06	4.11E-05
M1 DG-poly	M1 TG-poly	1	2.90E-15	3.96E-13
M1 DG-sat	M1 TG-sat	1	1.63E-20	6.70E-18
M1 DG-sat	M1 DG-mono	1	7.98E-07	3.12E-05
M1 FFA-mono	M1 FFA-poly	1	3.30E-22	2.71E-19
M1 FFA-sat	M1 FFA-mono	0.42	0.00323	0.0602
M1 GlcCer	M1 PLPC-sat	0.685	2.57E-05	0.000726
M1 LysoPC-mono	M1 LysoPC-poly	1	2.81E-06	1.00E-04
M1 LysoPC-poly	M1 LysoPE-poly	1	9.76E-19	2.67E-16
M1 LysoPC-poly	M1 LysoPE-sat	0.578	0.00312	0.0595
M1 LysoPC-sat	M1 LysoPC-mono	1	8.30E-12	7.56E-10
M1 LysoPC-sat	M1 LysoPE-sat	0.801	1.67E-05	0.000507
M1 PC-mono	M1 PE-mono	0.815	2.54E-05	0.000726
M1 PC-mono	M1 TG-mono	0.534	0.00188	0.0385
M1 PC-poly	M1 TG-poly	0.926	7.49E-07	3.07E-05
M1 PC-poly	M1 PE-poly	0.809	3.56E-06	0.000122
M1 PC-sat	M1 PC-mono	0.89	4.47E-06	0.000147
M1 PC-sat	M1 PE-sat	0.676	0.00106	0.0236
M1 PC-sat	M1 PLPE-sat	0.59	0.00181	0.038
M1 PC-sat	M1 TG-sat	0.606	0.00295	0.0577
M1 PE-mono	M1 PE-poly	0.918	2.95E-07	1.27E-05
M1 PE-mono	M1 PG-mono	0.474	0.00254	0.0509
M1 PE-sat	M1 PE-mono	1	2.41E-09	1.32E-07
M1 PE-sat	M1 PE-poly	0.615	0.000929	0.0218
M1 PE-sat	M1 TG-sat	0.599	0.00541	0.0986
M1 PI-mono	M1 PI-poly	1	9.23E-11	6.31E-09
M1 PI-sat	M1 PI-mono	1	8.16E-10	5.15E-08
M1 PI-sat	M1 PI-poly	1	5.49E-08	2.65E-06
M1 PLPC-mono	M1 PLPC-poly	0.766	3.84E-05	0.00105
M1 PLPC-mono	M1 PLPE-mono	0.627	0.001	0.0228
M1 PLPC-poly	M1 PLPE-poly	1	3.78E-11	2.82E-09
M1 PLPC-sat	M1 PLPC-mono	1	3.24E-11	2.66E-09
M1 PLPE-mono	M1 PLPE-poly	1	5.46E-08	2.65E-06
M1 PLPE-sat	M1 PLPE-mono	1	5.95E-12	6.09E-10
M1 SM-mono	M1 SM-poly	1	5.90E-17	9.68E-15

M1 SM-sat	M1 SM-mono	1	1.02E-09	6.00E-08
M3 AcylCN	M3 LysoPC-mono	0.812	1.85E-05	0.000583
M3 AcylCN	M3 FFA-poly	0.586	0.00011	0.00292
M3 CE	M3 CE-poly	0.546	9.39E-05	0.00257
M3 CE	M3 PI-sat	0.382	0.00248	0.0462
M3 CE	M3 PC-sat	0.465	0.00275	0.0481
M3 CE-poly	M3 DG-mono	-0.6	0.000637	0.0145
M3 CE-poly	M3 PC-poly	0.557	0.000879	0.0176
M3 CE-poly	M3 TG-mono	-0.444	0.00535	0.082
M3 CER-mono	M3 CER-poly	1	1.33E-17	1.09E-14
M3 CER-mono	M3 SM-sat	0.799	7.04E-05	0.00199
M3 CER-mono	M3 SM-poly	-0.495	0.00276	0.0481
M3 CER-poly	M3 SM-mono	0.637	0.000627	0.0145
M3 CER-sat	M3 CER-mono	0.799	9.30E-06	0.000318
M3 DG-mono	M3 DG-poly	1	4.31E-10	2.72E-08
M3 DG-mono	M3 TG-mono	0.759	2.34E-05	0.000711
M3 DG-poly	M3 TG-poly	1	2.01E-11	1.50E-09
M3 DG-sat	M3 TG-sat	1	2.58E-14	5.30E-12
M3 DG-sat	M3 DG-mono	1	4.64E-10	2.72E-08
M3 FFA-mono	M3 FFA-poly	1	2.31E-14	5.30E-12
M3 FFA-sat	M3 FFA-mono	0.428	0.00258	0.047
M3 FFA-sat	M3 LysoPE-poly	0.354	0.0054	0.082
M3 GlcCer	M3 PLPC-sat	0.787	1.09E-06	4.27E-05
M3 LysoPC-mono	M3 LysoPC-poly	1	2.62E-12	2.39E-10
M3 LysoPC-poly	M3 LysoPE-poly	1	5.55E-09	2.84E-07
M3 LysoPC-sat	M3 LysoPC-mono	1	6.05E-11	4.13E-09
M3 LysoPC-sat	M3 LysoPE-sat	1	8.29E-09	4.00E-07
M3 LysoPC-sat	M3 LysoPC-poly	0.812	0.000762	0.0164
M3 PC-mono	M3 PE-mono	0.644	0.000934	0.0182
M3 PC-poly	M3 TG-poly	0.989	2.03E-08	9.27E-07
M3 PC-poly	M3 PE-poly	0.735	2.35E-06	8.76E-05
M3 PC-poly	M3 PI-poly	0.501	0.00362	0.0619
M3 PC-poly	M3 PLPC-poly	0.487	0.00514	0.0811
M3 PC-sat	M3 PC-mono	1	8.94E-14	1.47E-11
M3 PC-sat	M3 TG-sat	1	1.61E-07	6.94E-06
M3 PE-mono	M3 PG-mono	0.521	0.00208	0.0397
M3 PE-sat	M3 PE-mono	1	1.32E-11	1.09E-09
M3 PE-sat	M3 PE-poly	0.535	0.000784	0.0165
M3 PE-sat	M3 TG-sat	0.552	0.00443	0.0712
M3 PG-mono	M3 PI-mono	0.563	0.000212	0.00543
M3 PG-mono	M3 TG-mono	0.43	0.00439	0.0712
M3 PG-sat	M3 PI-poly	0.604	1.41E-05	0.000461
M3 PI-mono	M3 PI-poly	1	2.73E-13	3.74E-11
M3 PI-sat	M3 PI-poly	0.539	0.000355	0.00883
M3 PI-sat	M3 PI-mono	0.496	0.000807	0.0165
M3 PLPC-mono	M3 PLPC-poly	0.559	0.000381	0.00919
M3 PLPC-mono	M3 PLPE-mono	0.552	0.000741	0.0164
M3 PLPC-poly	M3 PLPE-poly	1	7.87E-10	4.30E-08

M3 PLPC-sat	M3 PLPC-mono	0.776	1.00E-06	4.11E-05
M3 PLPC-sat	M3 PLPE-mono	0.503	0.00412	0.069
M3 PLPE-mono	M3 PLPE-poly	0.821	3.45E-06	0.000123
M3 PLPE-sat	M3 PLPE-mono	1	2.37E-15	9.70E-13
M3 SM-mono	M3 SM-poly	1	4.02E-13	4.56E-11
M3 SM-sat	M3 SM-mono	1	4.45E-13	4.56E-11
M3 TG-sat	M3 TG-mono	0.724	4.27E-05	0.00125
CB AcylCN	CB SM-poly	0.331	0.00447	0.0833
CB CE	CB LysoPC-mono	0.456	0.00226	0.0514
CB CE	CB CE-poly	0.377	0.00425	0.081
CB CE-poly	CB PLPC-poly	0.694	4.01E-05	0.00132
CB CE-poly	CB LysoPC-poly	0.664	0.00023	0.00672
CB CE-poly	CB DG-mono	-0.594	0.00191	0.046
CB CE-poly	CB SM-poly	0.374	0.00555	0.0989
CB CER-mono	CB CER-poly	1	6.93E-09	7.10E-07
CB CER-sat	CB CER-poly	0.936	2.55E-06	0.000105
CB CER-sat	CB LysoPC-sat	0.636	0.000333	0.00943
CB DG-mono	CB DG-poly	1	8.83E-08	5.20E-06
CB DG-mono	CB TG-mono	1	2.09E-06	9.50E-05
CB DG-mono	CB PI-mono	0.715	0.00251	0.0556
CB DG-mono	CB TG-poly	-0.476	0.00543	0.0989
CB DG-poly	CB TG-poly	0.948	4.76E-10	9.76E-08
CB DG-sat	CB TG-sat	1	2.36E-06	0.000102
CB DG-sat	CB DG-mono	1	4.01E-06	0.000149
CB FFA-mono	CB FFA-poly	1	7.13E-19	5.85E-16
CB FFA-sat	CB FFA-mono	0.552	0.00046	0.0126
CB GlcCer	CB SM-mono	0.808	0.000106	0.00335
CB LysoPC-mono	CB LysoPC-poly	0.972	3.42E-06	0.000134
CB LysoPC-poly	CB LysoPE-poly	1	1.32E-12	5.41E-10
CB LysoPC-sat	CB LysoPC-mono	1	1.31E-09	1.80E-07
CB LysoPC-sat	CB LysoPE-sat	1	9.43E-09	8.59E-07
CB PC-mono	CB PE-mono	1	6.68E-10	1.10E-07
CB PC-poly	CB PE-poly	1	1.87E-06	9.03E-05
CB PC-poly	CB TG-mono	0.608	0.00262	0.0564
CB PC-poly	CB PLPC-poly	0.523	0.00416	0.081
CB PC-sat	CB PLPC-sat	1	4.99E-08	3.41E-06
CB PE-mono	CB PE-poly	1	0.000953	0.0244
CB PE-sat	CB PE-poly	1	1.25E-07	6.81E-06
CB PE-sat	CB PLPE-mono	1	0.000136	0.00414
CB PG-sat	CB PLPC-sat	0.519	0.000555	0.0147
CB PG-sat	CB PI-poly	0.344	0.0027	0.0568
CB PI-mono	CB PI-poly	1	3.21E-11	8.77E-09
CB PI-sat	CB PI-mono	1	1.53E-06	7.83E-05
CB PI-sat	CB PLPE-mono	0.84	0.0034	0.0697
CB PLPC-mono	CB PLPC-poly	0.619	0.00206	0.0482
CB PLPC-sat	CB PLPC-mono	1	8.87E-08	5.20E-06
CB PLPE-mono	CB PLPE-poly	1	2.86E-05	0.000977
CB PLPE-sat	CB PLPE-poly	1	4.86E-09	5.69E-07

CB PLPE-sat	CB PLPE-mono	0.64	0.0037	0.074
CB SM-mono	CB SM-poly	0.939	2.93E-08	2.40E-06
CB SM-sat	CB SM-mono	1	4.45E-08	3.32E-06
CB TG-mono	CB TG-poly	0.524	0.00183	0.0455
CB TG-sat	CB TG-mono	1	9.81E-06	0.00035
M1-GlcCer	M3-GlcCer	0.701	1.99E-06	0.000165
M1-PE-poly	M3-PE-poly	0.603	3.30E-05	0.00192
M1-SM-poly	M3-SM-poly	0.537	0.000114	0.00612
M1-PE-mono	M3-PE-mono	0.72	0.000207	0.0107
M1-PI-mono	M3-PI-mono	0.62	0.000339	0.0163
M1-PLPE-poly	M3-PLPE-poly	0.622	0.00033	0.0163
M1-PC-poly	M3-PC-poly	0.545	0.0019	0.0734
M1-SM-sat	M3-SM-sat	0.577	0.0024	0.0896

Supplementary Table S4. Association between maternal baseline BMI with lipid groups. Regression models estimated the linear relationship between maternal baseline BMI and M1, M3, and CB lipid groups, adjusting for sex, parity, maternal age, and gestational age. Significance denoted by unadjusted p-value (α=0.05, highlighted in red) and an FDR adjusted p-value (α=0.10, highlighted in green).

Lipid group	*Maternal First Trimester*				*Maternal term*				*Cord blood*			
	Beta Coefficient	Std Error	unadjusted p-value	FDR	Beta Coefficient	Std Error	unadjusted p-value	FDR	Beta Coefficient	Std Error	unadjusted p-value	FDR
AC	0.0003	0.0165	0.9833	0.9876	-0.0045	0.0163	0.7822	0.8223	0.0205	0.0158	0.1941	0.9162
CE	0.0374	0.0158	**0.0180**	0.1230	-0.0353	0.0162	**0.0292**	0.1332	-0.0428	0.0153	**0.0052**	0.1854
CE-poly	0.0003	0.0165	0.9836	0.9876	-0.0143	0.0162	0.3790	0.4383	0.0062	0.0153	0.6843	0.9162
CER-sat	0.0217	0.0158	0.1698	0.3853	-0.0291	0.0159	0.0679	0.1710	-0.0152	0.0160	0.3408	0.9162
CER-mono	-0.0074	0.0164	0.6512	0.8613	-0.0292	0.0155	0.0601	0.1710	-0.0026	0.0163	0.8715	0.9162
CER-poly	-0.0086	0.0162	0.5961	0.8146	-0.0001	0.0161	0.9955	0.9955	-0.0088	0.0163	0.5906	0.9162
DG-sat	0.0089	0.0162	0.5810	0.8146	-0.0103	0.0163	0.5271	0.5841	0.0096	0.0153	0.5303	0.9162
DG-mono	0.0276	0.0158	0.0814	0.2225	-0.0143	0.0164	0.3848	0.4383	0.0046	0.0161	0.7723	0.9162
DG-poly	0.0316	0.0157	**0.0437**	0.1792	-0.0083	0.0164	0.6140	0.6624	0.0067	0.0160	0.6767	0.9162
FFA-sat	-0.0013	0.0161	0.9382	0.9876	0.0006	0.0164	0.9713	0.9955	-0.0059	0.0157	0.7061	0.9162
FFA-mono	0.0037	0.0164	0.8212	0.9876	-0.0266	0.0161	0.0975	0.1980	-0.0102	0.0157	0.5141	0.9162
FFA-poly	0.0212	0.0165	0.1988	0.3882	-0.0142	0.0163	0.3826	0.4383	0.0054	0.0159	0.7333	0.9162
GlcCer	-0.0340	0.0163	**0.0375**	0.1766	-0.0257	0.0161	0.1092	0.1980	0.0091	0.0164	0.5790	0.9162
LysoPC-sat	-0.0238	0.0163	0.1446	0.3706	-0.0285	0.0158	0.0709	0.1710	0.0188	0.0162	0.2483	0.9162
LysoPC-mono	-0.0278	0.0160	0.0814	0.2225	-0.0264	0.0159	0.0961	0.1980	0.0096	0.0164	0.5604	0.9162
LysoPC-poly	-0.0035	0.0156	0.8211	0.9876	-0.0205	0.0161	0.2019	0.2855	0.0102	0.0159	0.5205	0.9162
LysoPE-sat	-0.0294	0.0156	0.0595	0.1878	-0.0165	0.0163	0.3118	0.3873	0.0185	0.0162	0.2545	0.9162
LysoPE-poly	-0.0111	0.0151	0.4593	0.6975	-0.0297	0.0162	0.0664	0.1710	0.0066	0.0156	0.6738	0.9162
PC-sat	-0.0162	0.0161	0.3160	0.5632	-0.0551	0.0154	**0.0003**	0.0046	-0.0033	0.0168	0.8431	0.9162
PC-mono	-0.0003	0.0165	0.9876	0.9876	-0.0479	0.0160	**0.0028**	0.0222	-0.0078	0.0167	0.6396	0.9162
PC-poly	-0.0328	0.0159	**0.0388**	0.1766	-0.0580	0.0152	**0.0001**	0.0042	0.0182	0.0165	0.2699	0.9162
PE-sat	-0.0158	0.0163	0.3319	0.5670	-0.0260	0.0162	0.1100	0.1980	0.0041	0.0161	0.7975	0.9162
PE-mono	-0.0123	0.0165	0.4559	0.6975	-0.0257	0.0166	0.1215	0.1980	0.0010	0.0165	0.9539	0.9539
PE-poly	-0.0092	0.0163	0.5701	0.8146	-0.0286	0.0164	0.0815	0.1856	0.0195	0.0163	0.2313	0.9162
PG-sat	0.0132	0.0155	0.3923	0.6433	-0.0240	0.0162	0.1389	0.2034	0.0322	0.0162	**0.0460**	0.6293
PG-mono	-0.0014	0.0162	0.9304	0.9876	-0.0415	0.0160	**0.0095**	0.0488	0.0043	0.0157	0.7845	0.9162
PI-sat	0.0061	0.0162	0.7052	0.9036	-0.0252	0.0164	0.1255	0.1980	0.0031	0.0166	0.8524	0.9162
PI-mono	0.0211	0.0162	0.1926	0.3882	-0.0319	0.0162	**0.0489**	0.1672	0.0064	0.0162	0.6946	0.9162
PI-poly	0.0223	0.0160	0.1634	0.3853	-0.0331	0.0162	**0.0406**	0.1627	0.0172	0.0156	0.2700	0.9162
PLPC-sat	-0.0390	0.0162	**0.0164**	0.1230	-0.0506	0.0155	**0.0011**	0.0111	0.0150	0.0159	0.3467	0.9162
PLPC-mono	-0.0370	0.0162	**0.0223**	0.1303	-0.0193	0.0167	0.2459	0.3253	0.0212	0.0161	0.1882	0.9162
PLPC-poly	-0.0312	0.0162	0.0542	0.1878	-0.0175	0.0161	0.2758	0.3534	0.0049	0.0165	0.7692	0.9162
PLPE-sat	-0.0302	0.0159	0.0573	0.1878	-0.0451	0.0158	**0.0042**	0.0247	0.0218	0.0152	0.1520	0.9162
PLPE-mono	-0.0577	0.0153	**0.0002**	0.0023	-0.0583	0.0157	**0.0002**	0.0042	-0.0046	0.0161	0.7767	0.9162
PLPE-poly	-0.0379	0.0157	**0.0157**	0.1230	-0.0310	0.0161	0.0550	0.1710	0.0089	0.0163	0.5844	0.9162
SM-sat	0.0182	0.0164	0.2670	0.4976	-0.0199	0.0162	0.2176	0.2974	-0.0049	0.0167	0.7681	0.9162
SM-mono	0.0678	0.0153	**0.0000**	0.0002	0.0318	0.0158	**0.0436**	0.1627	0.0190	0.0162	0.2394	0.9162
SM-poly	0.0743	0.0142	**0.0000**	0.0000	0.0460	0.0156	**0.0032**	0.0222	0.0404	0.0155	**0.0090**	0.1854
TG-sat	-0.0022	0.0165	0.8944	0.9876	-0.0258	0.0163	0.1137	0.1980	0.0034	0.0160	0.8290	0.9162
TG-mono	-0.0028	0.0162	0.8608	0.9876	-0.0256	0.0165	0.1202	0.1980	0.0091	0.0152	0.5497	0.9162
TG-poly	0.0215	0.0160	0.1786	0.3853	-0.0240	0.0160	0.1331	0.2021	0.0016	0.0163	0.9207	0.9437

Supplementary Table S5. Association between maternal gestational weight gain with lipid groups. Regression models estimated the linear relationship between maternal GWG (kg) and M1, M3, and CB lipid groups, adjusting for sex, parity, maternal age, and gestational age. Significance denoted by unadjusted p-value (α=0.05, highlighted in red) and an FDR adjusted p-value (α=0.10, highlighted in green).

Lipid group	*Maternal First Trimester*				*Maternal term*				*Cord blood*			
	Beta Coefficient	Std Error	unadjusted p-value	FDR	Beta Coefficient	Std Error	unadjusted p-value	FDR	Beta Coefficient	Std Error	unadjusted p-value	FDR
AC	0.0253	0.0188	0.1781	0.5438	0.0077	0.0185	0.6788	0.9082	0.0097	0.0182	0.5949	0.9971
CE	-0.0034	0.0186	0.8541	0.8979	0.0324	0.0185	0.0805	0.2750	0.0170	0.0180	0.3442	0.9971
CE-poly	0.0041	0.0190	0.8306	0.8962	0.0178	0.0185	0.3357	0.5984	0.0099	0.0178	0.5770	0.9971
CER-sat	0.0145	0.0185	0.4340	0.7258	0.0409	0.0183	**0.0251**	0.1078	0.0332	0.0180	0.0655	0.9971
CER-mono	0.0232	0.0186	0.2122	0.5438	0.0287	0.0185	0.1219	0.3125	0.0006	0.0186	0.9756	0.9971
CER-poly	-0.0052	0.0185	0.7774	0.8704	-0.0166	0.0188	0.3793	0.6479	0.0020	0.0185	0.9149	0.9971
DG-sat	0.0187	0.0183	0.3086	0.6468	0.0194	0.0185	0.2940	0.5739	0.0202	0.0174	0.2465	0.9971
DG-mono	-0.0159	0.0182	0.3826	0.7155	-0.0036	0.0187	0.8476	0.9676	0.0057	0.0183	0.7555	0.9971
DG-poly	-0.0232	0.0181	0.1995	0.5438	0.0029	0.0188	0.8756	0.9676	0.0165	0.0183	0.3690	0.9971
FFA-sat	-0.0016	0.0189	0.9337	0.9407	-0.0002	0.0186	0.9904	0.9904	0.0126	0.0185	0.4946	0.9971
FFA-mono	-0.0093	0.0189	0.6230	0.8704	-0.0203	0.0185	0.2743	0.5624	-0.0126	0.0180	0.4833	0.9971
FFA-poly	-0.0076	0.0192	0.6902	0.8704	-0.0095	0.0188	0.6120	0.8961	-0.0013	0.0182	0.9445	0.9971
GlcCer	-0.0065	0.0190	0.7339	0.8704	-0.0182	0.0185	0.3264	0.5984	0.0007	0.0187	0.9685	0.9971
LysoPC-sat	0.0394	0.0184	**0.0325**	0.2606	0.0478	0.0179	**0.0075**	0.0439	0.0175	0.0188	0.3532	0.9971
LysoPC-mono	0.0284	0.0184	0.1224	0.4563	0.0381	0.0179	**0.0336**	0.1252	-0.0048	0.0191	0.8034	0.9971
LysoPC-poly	0.0416	0.0181	**0.0216**	0.2606	0.0566	0.0177	**0.0014**	0.0157	0.0081	0.0184	0.6593	0.9971
LysoPE-sat	0.0232	0.0181	0.1986	0.5438	0.0535	0.0180	**0.0029**	0.0238	0.0169	0.0190	0.3739	0.9971
LysoPE-poly	0.0370	0.0179	**0.0393**	0.2606	0.0680	0.0177	**0.0001**	0.0024	0.0112	0.0179	0.5297	0.9971
PC-sat	0.0377	0.0180	**0.0368**	0.2606	0.0510	0.0181	**0.0048**	0.0328	-0.0029	0.0191	0.8810	0.9971
PC-mono	0.0348	0.0185	0.0606	0.3108	0.0459	0.0185	**0.0131**	0.0673	0.0091	0.0190	0.6329	0.9971
PC-poly	0.0328	0.0184	0.0743	0.3383	0.0403	0.0181	**0.0263**	0.1078	-0.0056	0.0189	0.7683	0.9971
PE-sat	0.0212	0.0185	0.2528	0.5997	0.0206	0.0188	0.2736	0.5624	0.0003	0.0184	0.9869	0.9971
PE-mono	0.0163	0.0189	0.3872	0.7155	0.0132	0.0191	0.4892	0.8023	0.0001	0.0187	0.9971	0.9971
PE-poly	0.0076	0.0186	0.6814	0.8704	0.0118	0.0189	0.5331	0.8406	-0.0007	0.0187	0.9701	0.9971
PG-sat	0.0014	0.0190	0.9407	0.9407	-0.0006	0.0190	0.9732	0.9904	-0.0034	0.0188	0.8558	0.9971
PG-mono	-0.0185	0.0184	0.3155	0.6468	-0.0022	0.0188	0.9064	0.9676	0.0045	0.0179	0.7991	0.9971
PI-sat	0.0085	0.0186	0.6490	0.8704	0.0070	0.0189	0.7099	0.9082	-0.0063	0.0189	0.7371	0.9971
PI-mono	-0.0069	0.0186	0.7120	0.8704	0.0019	0.0188	0.9204	0.9676	-0.0206	0.0186	0.2687	0.9971
PI-poly	-0.0094	0.0185	0.6108	0.8704	0.0055	0.0188	0.7720	0.9310	-0.0382	0.0186	**0.0402**	0.9971
PLPC-sat	0.0059	0.0190	0.7570	0.8704	0.0064	0.0186	0.7310	0.9082	-0.0058	0.0182	0.7510	0.9971
PLPC-mono	0.0053	0.0189	0.7791	0.8704	0.0098	0.0190	0.6051	0.8961	-0.0195	0.0185	0.2920	0.9971
PLPC-poly	0.0144	0.0188	0.4426	0.7258	0.0020	0.0184	0.9129	0.9676	0.0042	0.0191	0.8244	0.9971
PLPE-sat	0.0273	0.0183	0.1350	0.4611	0.0307	0.0189	0.1040	0.3125	-0.0044	0.0178	0.8052	0.9971
PLPE-mono	0.0368	0.0183	**0.0445**	0.2606	0.0248	0.0190	0.1916	0.4364	0.0052	0.0184	0.7769	0.9971
PLPE-poly	0.0303	0.0182	0.0962	0.3943	0.0258	0.0185	0.1625	0.3920	0.0029	0.0188	0.8789	0.9971
SM-sat	0.0158	0.0188	0.4014	0.7155	0.0086	0.0186	0.6450	0.9082	0.0081	0.0190	0.6720	0.9971
SM-mono	-0.0469	0.0185	**0.0113**	0.2325	-0.0558	0.0176	**0.0015**	0.0157	-0.0073	0.0186	0.6961	0.9971
SM-poly	-0.0578	0.0178	**0.0011**	0.0459	-0.0786	0.0172	**0.0000**	0.0002	-0.0209	0.0184	0.2550	0.9971
TG-sat	0.0208	0.0186	0.2633	0.5997	0.0297	0.0187	0.1122	0.3125	0.0036	0.0181	0.8423	0.9971
TG-mono	0.0117	0.0186	0.5317	0.8385	-0.0067	0.0191	0.7263	0.9082	0.0029	0.0174	0.8658	0.9971
TG-poly	0.0050	0.0184	0.7854	0.8704	0.0288	0.0183	0.1158	0.3125	0.0220	0.0185	0.2340	0.9971

Supplementary Table S6. Association between lipid groups and birth weight, stratified by sex. Regression models estimated the linear relationship between M1, M3, and CB lipid groups and Fenton birth weight z-score, adjusting for sex, parity, maternal age, gestational age, maternal baseline BMI, and maternal GWG. Sex stratified models were run. Significance denoted by unadjusted p-value (α=0.05, highlighted in red) and an FDR adjusted p-value (α=0.10, highlighted in green).

Lipid Group	All				Males				Females			
	Beta Coefficient	Std Error	unadjusted p-value	FDR	Beta Coefficient	Std Error	unadjusted p-value	FDR	Beta Coefficient	Std Error	unadjusted p-value	FDR
Maternal First Trimester												
AC	0.0289	0.0766	0.7063	0.9685	0.1229	0.1121	0.2728	0.7006	-0.0415	0.1037	0.6889	0.9151
CE	-0.0365	0.0788	0.6434	0.9685	-0.1451	0.1187	0.2218	0.7006	0.0985	0.1073	0.3584	0.9151
CE-poly	0.0475	0.0752	0.5276	0.9685	0.0390	0.1264	0.7578	0.9138	0.0892	0.0958	0.3514	0.9151
CER-sat	-0.0535	0.0791	0.4987	0.9685	-0.0397	0.1107	0.7199	0.8944	-0.0531	0.1162	0.6476	0.9151
CER-mono	-0.1211	0.0754	0.1082	0.9685	-0.2700	0.1068	**0.0115**	0.1355	0.0685	0.1045	0.5120	0.9151
CER-poly	-0.1191	0.0765	0.1196	0.9685	-0.2950	0.1065	**0.0056**	0.1150	0.1247	0.1105	0.2593	0.9151
DG-sat	0.0352	0.0775	0.6495	0.9685	0.0976	0.1014	0.3357	0.7006	-0.0635	0.1338	0.6354	0.9151
DG-mono	-0.0627	0.0788	0.4266	0.9685	-0.0876	0.1413	0.5351	0.8286	-0.0601	0.0955	0.5291	0.9151
DG-poly	-0.1350	0.0784	0.0852	0.9685	-0.2449	0.1372	0.0742	0.4344	-0.0850	0.0947	0.3694	0.9151
FFA-sat	0.0889	0.0766	0.2454	0.9685	0.2884	0.1255	**0.0216**	0.1772	-0.0627	0.0939	0.5040	0.9151
FFA-mono	0.0028	0.0767	0.9704	0.9727	0.1051	0.1035	0.3100	0.7006	-0.1402	0.1123	0.2118	0.9151
FFA-poly	-0.0212	0.0760	0.7799	0.9685	0.0026	0.1067	0.9808	0.9898	-0.0682	0.1084	0.5291	0.9151
GlcCer	0.0177	0.0766	0.8172	0.9685	-0.1076	0.1158	0.3526	0.7006	0.1212	0.1012	0.2308	0.9151
LysoPC-sat	-0.0945	0.0765	0.2170	0.9685	-0.1537	0.1124	0.1715	0.7006	0.0006	0.1071	0.9955	0.9955
LysoPC-mono	-0.0493	0.0782	0.5281	0.9685	-0.0214	0.1168	0.8543	0.9533	-0.0295	0.1119	0.7923	0.9151
LysoPC-poly	-0.0246	0.0810	0.7616	0.9685	0.0655	0.1180	0.5788	0.8286	-0.0704	0.1252	0.5740	0.9151
LysoPE-sat	-0.0813	0.0794	0.3061	0.9685	-0.0620	0.1154	0.5909	0.8286	-0.0917	0.1241	0.4599	0.9151
LysoPE-poly	-0.0359	0.0831	0.6658	0.9685	0.0281	0.1141	0.8054	0.9434	-0.0821	0.1368	0.5482	0.9151
PC-sat	-0.0427	0.0783	0.5856	0.9685	-0.0684	0.1142	0.5496	0.8286	0.0513	0.1124	0.6479	0.9151
PC-mono	-0.0796	0.0766	0.2987	0.9685	-0.2831	0.1299	**0.0293**	0.2005	0.0713	0.0937	0.4467	0.9151
PC-poly	-0.0100	0.0791	0.8991	0.9704	-0.0014	0.1122	0.9898	0.9898	0.0305	0.1151	0.7909	0.9151
PE-sat	0.0155	0.0768	0.8401	0.9685	-0.0195	0.1108	0.8603	0.9533	0.1019	0.1087	0.3484	0.9151
PE-mono	0.0235	0.0754	0.7558	0.9685	-0.1206	0.1222	0.3237	0.7006	0.1458	0.0922	0.1137	0.9151
PE-poly	0.0326	0.0765	0.6706	0.9685	-0.0556	0.1045	0.5945	0.8286	0.1921	0.1118	0.0856	0.9151
PG-sat	-0.0331	0.0804	0.6801	0.9685	-0.0564	0.1094	0.6063	0.8286	-0.0101	0.1234	0.9347	0.9826
PG-mono	-0.0564	0.0769	0.4636	0.9685	-0.1859	0.1083	0.0859	0.4404	0.1220	0.1194	0.3070	0.9151
PI-sat	-0.0097	0.0771	0.8994	0.9704	0.1315	0.1545	0.3947	0.7355	-0.0602	0.0855	0.4816	0.9151
PI-mono	-0.0536	0.0767	0.4846	0.9685	-0.2608	0.2181	0.2319	0.7006	-0.0322	0.0792	0.6847	0.9151
PI-poly	-0.0537	0.0774	0.4874	0.9685	-0.1358	0.1480	0.3588	0.7006	-0.0274	0.0931	0.7681	0.9151
PLPC-sat	0.0026	0.0768	0.9727	0.9727	-0.0055	0.1219	0.9639	0.9898	0.0424	0.0994	0.6695	0.9151
PLPC-mono	-0.0145	0.0770	0.8503	0.9685	-0.0111	0.1307	0.9323	0.9898	0.0090	0.0957	0.9251	0.9826
PLPC-poly	-0.0033	0.0766	0.9656	0.9727	0.0900	0.1308	0.4916	0.8286	-0.0285	0.0937	0.7611	0.9151
PLPE-sat	-0.0566	0.0785	0.4709	0.9685	-0.0405	0.1124	0.7183	0.8944	-0.0344	0.1216	0.7773	0.9151
PLPE-mono	-0.0587	0.0815	0.4715	0.9685	-0.0571	0.1249	0.6475	0.8564	-0.0197	0.1119	0.8605	0.9535
PLPE-poly	0.0171	0.0798	0.8304	0.9685	0.1405	0.1261	0.2655	0.7006	-0.0272	0.1092	0.8035	0.9151
SM-sat	-0.1225	0.0761	0.1075	0.9685	-0.3660	0.1141	**0.0013**	0.0549	0.0947	0.1016	0.3511	0.9151
SM-mono	-0.0938	0.0817	0.2510	0.9685	-0.3231	0.1304	**0.0132**	0.1355	0.0910	0.1035	0.3792	0.9151
SM-poly	0.0269	0.0889	0.7625	0.9685	-0.1422	0.1483	0.3379	0.7006	0.1270	0.1090	0.2437	0.9151
TG-sat	0.0456	0.0760	0.5483	0.9685	0.1069	0.0998	0.2839	0.7006	0.0043	0.1319	0.9741	0.9955
TG-mono	-0.1332	0.0758	0.0789	0.9685	-0.2099	0.1484	0.1572	0.7006	-0.1062	0.0898	0.2366	0.9151
TG-poly	-0.0851	0.0776	0.2727	0.9685	-0.0608	0.1143	0.5950	0.8286	-0.0948	0.1059	0.3707	0.9151
Maternal Term												
AC	0.1059	0.0758	0.1622	0.4122	0.2052	0.1050	0.0507	0.7164	0.0182	0.1106	0.8690	0.9733
CE	-0.0112	0.0771	0.8849	0.9743	0.0150	0.1167	0.8980	0.9929	0.0242	0.1155	0.8338	0.9733
CE-poly	-0.1147	0.0760	0.1312	0.4046	0.0850	0.1234	0.4912	0.8391	-0.3029	0.1092	**0.0055**	0.0757
CER-sat	-0.0248	0.0798	0.7561	0.9394	-0.0011	0.1215	0.9929	0.9929	-0.0133	0.1080	0.9021	0.9733
CER-mono	0.0498	0.0811	0.5388	0.8574	-0.0117	0.1235	0.9244	0.9929	0.0944	0.1043	0.3653	0.7324
CER-poly	0.1323	0.0800	0.0982	0.3967	0.1008	0.1182	0.3939	0.8253	0.1724	0.1057	0.1030	0.3440
DG-sat	0.1475	0.0751	**0.0494**	0.3377	0.1260	0.0993	0.2046	0.7164	0.1842	0.1231	0.1346	0.3943
DG-mono	0.1146	0.0750	0.1265	0.4046	0.0898	0.1223	0.4630	0.8253	0.1126	0.1019	0.2692	0.6131
DG-poly	0.0109	0.0756	0.8854	0.9743	0.0291	0.1266	0.8179	0.9929	-0.0449	0.0977	0.6456	0.9127
FFA-sat	-0.0721	0.0758	0.3419	0.7009	-0.0732	0.1575	0.6420	0.9749	-0.0609	0.0838	0.4675	0.7966
FFA-mono	0.0503	0.0784	0.5214	0.8574	0.0575	0.1134	0.6124	0.9657	0.0508	0.1086	0.6396	0.9127
FFA-poly	-0.0107	0.0767	0.8893	0.9743	-0.0199	0.1169	0.8647	0.9929	-0.0022	0.1025	0.9832	0.9832
GlcCer	0.1437	0.0773	0.0629	0.3687	0.0376	0.1310	0.7740	0.9929	0.2379	0.0950	**0.0123**	0.1010
LysoPC-sat	0.1123	0.0793	0.1567	0.4122	0.1065	0.0986	0.2799	0.7267	0.1273	0.1436	0.3752	0.7324
LysoPC-mono	0.1262	0.0782	0.1064	0.3967	0.1494	0.0940	0.1122	0.7164	0.0753	0.1538	0.6242	0.9127
LysoPC-poly	0.0705	0.0800	0.3781	0.7330	0.1454	0.0958	0.1291	0.7164	-0.1060	0.1520	0.4858	0.7966
LysoPE-sat	0.1735	0.0770	**0.0244**	0.2497	0.1305	0.1048	0.2134	0.7164	0.2265	0.1148	**0.0485**	0.2810
LysoPE-poly	-0.1094	0.0799	0.1709	0.4122	-0.1774	0.1215	0.1444	0.7164	-0.0566	0.1074	0.5984	0.9127
PC-sat	0.0347	0.0815	0.6702	0.9159	-0.0123	0.1368	0.9284	0.9929	0.0986	0.1011	0.3295	0.7111
PC-mono	-0.0186	0.0783	0.8126	0.9743	-0.1345	0.1301	0.3013	0.7267	0.0813	0.0999	0.4159	0.7435
PC-poly	0.0314	0.0825	0.7038	0.9308	0.0955	0.1115	0.3917	0.8253	-0.0446	0.1262	0.7239	0.9733
PE-sat	0.0410	0.0765	0.5919	0.8667	-0.0872	0.1132	0.4413	0.8253	0.2003	0.1072	0.0617	0.2810
PE-mono	0.0360	0.0751	0.6319	0.8934	-0.1878	0.1118	0.0931	0.7164	0.2453	0.0966	**0.0111**	0.1010
PE-poly	0.0789	0.0753	0.2949	0.6365	-0.0032	0.1038	0.9758	0.9929	0.1931	0.1139	0.0900	0.3354
PG-sat	0.0831	0.0768	0.2792	0.6360	0.0246	0.1190	0.8362	0.9929	0.0931	0.1147	0.4171	0.7435
PG-mono	0.0493	0.0781	0.5281	0.8574	-0.1704	0.1254	0.1742	0.7164	0.1812	0.0963	0.0600	0.2810
PI-sat	0.0261	0.0756	0.7298	0.9350	0.0874	0.1174	0.4566	0.8253	-0.0236	0.1190	0.8425	0.9733
PI-mono	-0.0057	0.0770	0.9407	0.9802	-0.0491	0.1180	0.6772	0.9916	0.0048	0.1075	0.9645	0.9832
PI-poly	0.0467	0.0770	0.5437	0.8574	0.1022	0.1281	0.4247	0.8253	-0.0034	0.0980	0.9722	0.9832
PLPC-sat	0.1943	0.0787	**0.0135**	0.1848	0.2165	0.1129	0.0552	0.7164	0.1829	0.1142	0.1091	0.3440
PLPC-mono	0.1289	0.0736	0.0800	0.3967	0.1847	0.1234	0.1343	0.7164	0.1105	0.0974	0.2563	0.6131
PLPC-poly	0.1142	0.0770	0.1381	0.4046	0.2236	0.1098	**0.0416**	0.7164	0.0155	0.1112	0.8893	0.9733
PLPE-sat	-0.0020	0.0789	0.9802	0.9802	-0.0076	0.1163	0.9482	0.9929	0.0149	0.1088	0.8908	0.9733

PLPE-mono	0.0028	0.0795	0.9723	0.9802	0.0436	0.1231	0.7232	0.9929	-0.0230	0.1035	0.8243	0.9733
PLPE-poly	0.0428	0.0772	0.5797	0.8667	0.1379	0.1141	0.2271	0.7164	-0.0222	0.1094	0.8393	0.9733
SM-sat	0.0657	0.0769	0.3933	0.7330	-0.0144	0.1373	0.9165	0.9929	0.1355	0.0932	0.1462	0.3995
SM-mono	0.2074	0.0789	**0.0086**	0.1756	0.1411	0.1326	0.2871	0.7267	0.3025	0.0974	**0.0019**	0.0390
SM-poly	0.2570	0.0797	**0.0013**	0.0522	0.2027	0.1547	0.1902	0.7164	0.3133	0.0879	**0.0004**	0.0150
TG-sat	0.1537	0.0751	**0.0408**	0.3342	0.1086	0.1042	0.2974	0.7267	0.2486	0.1166	**0.0330**	0.2256
TG-mono	0.1265	0.0750	0.0918	0.3967	0.0310	0.1168	0.7907	0.9929	0.1921	0.1063	0.0708	0.2903
TG-poly	-0.0095	0.0781	0.9030	0.9743	0.0561	0.1015	0.5802	0.9515	-0.1461	0.1250	0.2424	0.6131
Cord Blood												
AC	-0.0717	0.0789	0.3635	0.9130	0.0240	0.1082	0.8241	0.9384	-0.2222	0.1399	0.1123	0.3441
CE	0.1076	0.0805	0.1813	0.6195	0.1017	0.1126	0.3662	0.7909	0.1824	0.1177	0.1210	0.3441
CE-poly	0.1877	0.0811	**0.0206**	0.1056	0.3184	0.1177	**0.0068**	0.1350	0.0872	0.1076	0.4181	0.6874
CER-sat	0.0220	0.0785	0.7796	0.9130	0.0216	0.1118	0.8469	0.9384	0.0393	0.1117	0.7252	0.8674
CER-mono	-0.0237	0.0760	0.7548	0.9130	-0.0922	0.0953	0.3333	0.7909	0.1500	0.1296	0.2470	0.4823
CER-poly	0.0257	0.0764	0.7365	0.9130	-0.0253	0.1120	0.8215	0.9384	0.1062	0.1053	0.3133	0.5838
DG-sat	-0.0217	0.0826	0.7923	0.9130	-0.0048	0.1120	0.9657	0.9657	-0.0517	0.1259	0.6813	0.8640
DG-mono	-0.0244	0.0775	0.7530	0.9130	-0.0577	0.1319	0.6618	0.8797	-0.0156	0.0949	0.8693	0.8911
DG-poly	-0.1851	0.0758	**0.0146**	0.0852	-0.1291	0.1106	0.2432	0.7671	-0.3337	0.1053	**0.0015**	0.0313
FFA-sat	-0.1642	0.0781	**0.0356**	0.1623	-0.1622	0.1110	0.1439	0.6700	-0.1413	0.1137	0.2138	0.4383
FFA-mono	-0.0507	0.0797	0.5247	0.9130	-0.2121	0.1292	0.1006	0.6700	0.0786	0.1009	0.4359	0.6874
FFA-poly	-0.1088	0.0778	0.1621	0.6040	-0.1556	0.1098	0.1564	0.6700	-0.0355	0.1170	0.7616	0.8674
GlcCer	0.0671	0.0755	0.3742	0.9130	-0.0405	0.1087	0.7093	0.8797	0.1845	0.1007	0.0669	0.2605
LysoPC-sat	0.2338	0.0743	**0.0017**	0.0136	0.2666	0.1033	**0.0099**	0.1350	0.2100	0.1127	0.0623	0.2605
LysoPC-mono	0.2527	0.0717	**0.0004**	0.0043	0.2510	0.1017	**0.0136**	0.1393	0.2794	0.1022	**0.0063**	0.0427
LysoPC-poly	0.2667	0.0747	**0.0004**	0.0043	0.2605	0.1152	**0.0237**	0.1943	0.2699	0.0951	**0.0045**	0.0427
LysoPE-sat	0.3224	0.0700	**0.0000**	0.0002	0.4368	0.1107	**0.0001**	0.0032	0.2506	0.0903	**0.0055**	0.0427
LysoPE-poly	0.2167	0.0773	**0.0050**	0.0344	0.1629	0.1363	0.2319	0.7671	0.2461	0.0896	**0.0060**	0.0427
PC-sat	0.0506	0.0740	0.4943	0.9130	-0.0441	0.1003	0.6603	0.8797	0.1970	0.1068	0.0652	0.2605
PC-mono	0.0125	0.0747	0.8672	0.9130	-0.0817	0.0981	0.4051	0.7909	0.2050	0.1131	0.0699	0.2605
PC-poly	-0.0119	0.0761	0.8760	0.9130	0.0437	0.1131	0.6992	0.8797	-0.0646	0.1017	0.5252	0.7425
PE-sat	-0.0318	0.0773	0.6809	0.9130	-0.0606	0.0935	0.5172	0.8695	0.1195	0.1498	0.4250	0.6874
PE-mono	-0.0233	0.0755	0.7574	0.9130	-0.0972	0.0948	0.3050	0.7909	0.1695	0.1255	0.1766	0.3863
PE-poly	-0.0296	0.0763	0.6985	0.9130	-0.0499	0.0959	0.6031	0.8797	0.0334	0.1326	0.8010	0.8876
PG-sat	-0.0267	0.0770	0.7290	0.9130	-0.1323	0.1380	0.3379	0.7909	0.0420	0.0895	0.6394	0.8457
PG-mono	-0.0414	0.0791	0.6006	0.9130	-0.0636	0.0960	0.5075	0.8695	0.0812	0.1522	0.5935	0.8112
PI-sat	-0.0190	0.0752	0.8010	0.9130	-0.1252	0.0935	0.1805	0.6728	0.2415	0.1235	0.0506	0.2605
PI-mono	-0.0123	0.0770	0.8735	0.9130	-0.1064	0.1135	0.3485	0.7909	0.0789	0.1008	0.4340	0.6874
PI-poly	-0.0248	0.0809	0.7588	0.9130	-0.1032	0.1461	0.4801	0.8695	0.0117	0.0963	0.9033	0.9033
PLPC-sat	-0.0086	0.0789	0.9130	0.9130	-0.0553	0.1125	0.6229	0.8797	0.0753	0.1130	0.5051	0.7425
PLPC-mono	-0.0096	0.0777	0.9015	0.9130	-0.0168	0.1053	0.8732	0.9422	0.0239	0.1197	0.8420	0.8911
PLPC-poly	-0.0338	0.0765	0.6592	0.9130	-0.0670	0.1067	0.5302	0.8695	0.0359	0.1117	0.7477	0.8674
PLPE-sat	-0.0211	0.0817	0.7960	0.9130	0.0102	0.1116	0.9272	0.9657	-0.0551	0.1408	0.6954	0.8640
PLPE-mono	0.0136	0.0775	0.8603	0.9130	-0.0399	0.0942	0.6722	0.8797	0.2320	0.1470	0.1145	0.3441
PLPE-poly	0.0271	0.0766	0.7238	0.9130	-0.0064	0.0971	0.9476	0.9657	0.2056	0.1471	0.1623	0.3863
SM-sat	0.0423	0.0743	0.5693	0.9130	-0.0354	0.1024	0.7295	0.8797	0.1677	0.1096	0.1259	0.3441
SM-mono	0.0901	0.0765	0.2387	0.7528	0.0472	0.1333	0.7232	0.8797	0.1215	0.0902	0.1778	0.3863
SM-poly	0.0578	0.0804	0.4723	0.9130	0.2095	0.1337	0.1173	0.6700	-0.0654	0.1006	0.5155	0.7425
TG-sat	-0.0710	0.0809	0.3800	0.9130	-0.0858	0.1004	0.3928	0.7909	-0.0260	0.1416	0.8543	0.8911
TG-mono	-0.1168	0.0808	0.1484	0.6040	-0.1131	0.1322	0.3924	0.7909	-0.1329	0.0989	0.1790	0.3863
TG-poly	-0.2757	0.0717	**0.0001**	0.0025	-0.1569	0.1126	0.1634	0.6700	-0.4253	0.0836	**0.0000**	0.0000

Supplementary Table S7. Sex-specific relationship between cord blood triglycerides and birth weight. Regression models estimated the linear relationship between CB triglycerides and Fenton birth weight z-score, adjusting for sex, parity, maternal age, gestational age, maternal baseline BMI, and maternal GWG. Sex stratified models were run. Significance denoted by unadjusted p-value (α=0.05, highlighted in red).

Lipid	All			Males			Females		
	Beta Coefficient	Std Error	unadjusted p-value	Beta Coefficient	Std Error	unadjusted p-value	Beta Coefficient	Std Error	unadjusted p-value
TG 36:0	-0.5392	0.4198	0.1991	-0.5072	0.5612	0.3661	-0.405	0.6752	0.5486
TG 38:0	-0.2387	0.2587	0.3561	-0.2743	0.3037	0.3665	0.1985	0.5801	0.7322
TG 39:0	0.3291	0.1463	0.0245	0.4047	0.2036	0.0468	0.2279	0.2103	0.2785
TG 40:0	-0.1061	0.1189	0.3722	-0.1334	0.1329	0.3156	0.1709	0.332	0.6068
TG 40:1	-0.0944	0.1497	0.5286	-0.1609	0.183	0.3794	0.1192	0.2721	0.6613
TG 41:0	0.0745	0.0864	0.3886	-0.0078	0.1061	0.9410	0.2954	0.1558	0.0580
TG 42:0	-0.0777	0.0798	0.3299	-0.0815	0.0872	0.3503	0.0567	0.2846	0.8421
TG 42:1	-0.0662	0.0813	0.4157	-0.0875	0.0921	0.3419	0.093	0.2038	0.6479
TG 42:2	-0.0758	0.079	0.3373	-0.0965	0.0913	0.2904	0.0392	0.1754	0.8232
TG 42:3	-0.0728	0.0805	0.3654	-0.1147	0.1005	0.2537	0.0178	0.1455	0.9024
TG 43:0	0.1034	0.0796	0.1939	0.0468	0.1119	0.6761	0.1593	0.1137	0.1611
TG 44:0	-0.0649	0.0777	0.4035	-0.0878	0.0872	0.3141	0.1595	0.2045	0.4356
TG 44:1	-0.0823	0.0775	0.2880	-0.0916	0.0904	0.3113	-0.0175	0.1663	0.9162
TG 44:2	-0.0879	0.0765	0.2510	-0.0957	0.0868	0.2701	-0.012	0.1909	0.9500
TG 45:0	-0.0046	0.0919	0.9600	-0.0353	0.1194	0.7672	0.0874	0.15	0.5602
TG 45:1	-0.0614	0.0938	0.5125	-0.0628	0.12	0.6010	-0.038	0.1557	0.8072
TG 46:0	-0.0351	0.0778	0.6514	-0.0771	0.0966	0.4247	0.0864	0.1344	0.5204
TG 46:1	-0.0626	0.0787	0.4267	-0.0851	0.1003	0.3959	0.0033	0.1295	0.9797
TG 46:2	-0.0983	0.0788	0.2123	-0.1109	0.1042	0.2874	-0.0669	0.1211	0.5807
TG 46:3	-0.124	0.0782	0.1127	-0.1371	0.1023	0.1802	-0.0936	0.1227	0.4456
TG 47:0	0.0033	0.0948	0.9720	-0.0309	0.1304	0.8125	0.0457	0.1384	0.7412
TG 47:1	-0.0194	0.0881	0.8256	-0.0479	0.1192	0.6879	0.0508	0.1337	0.7041
TG 47:2	-0.014	0.0852	0.8693	-0.0187	0.125	0.8810	-0.0089	0.1141	0.9376
TG 47:3	-0.0768	0.0855	0.3686	-0.1003	0.1137	0.3778	-0.0294	0.1299	0.8207
TG 48:0	-0.0483	0.0809	0.5507	-0.1017	0.1244	0.4137	-0.0164	0.1044	0.8751
TG 48:1	-0.0797	0.0793	0.3146	-0.1082	0.1157	0.3497	-0.0478	0.1066	0.6540
TG 48:2	-0.0802	0.0805	0.3193	-0.1132	0.1343	0.3991	-0.0583	0.097	0.5477
TG 48:3	-0.1353	0.082	0.0988	-0.1667	0.133	0.2101	-0.1133	0.1004	0.2593
TG 48:4	-0.0939	0.0805	0.2434	-0.1473	0.1195	0.2176	-0.0552	0.1068	0.6052
TG 49:0	0.0498	0.0796	0.5319	0.0485	0.1257	0.6994	0.0445	0.1024	0.6634
TG 49:1	0.0069	0.0821	0.9329	-0.019	0.1245	0.8789	0.025	0.1071	0.8151
TG 49:2	-0.0054	0.0839	0.9489	0.0089	0.1371	0.9481	-0.0226	0.1024	0.8253
TG 49:3	-0.0622	0.084	0.4589	-0.111	0.1408	0.4304	-0.0453	0.1006	0.6529
TG 50:0	0.0168	0.078	0.8300	-0.034	0.1476	0.8179	0.0242	0.0886	0.7844
TG 50:1	-0.0915	0.079	0.2467	-0.1373	0.1412	0.3310	-0.0758	0.0918	0.4088
TG 50:2	-0.0993	0.0802	0.2157	-0.1647	0.1561	0.2916	-0.0793	0.0893	0.3745
TG 50:3	-0.1181	0.0818	0.1488	-0.1773	0.1629	0.2764	-0.1095	0.0901	0.2245
TG 50:4	-0.1169	0.0837	0.1625	-0.1287	0.1483	0.3853	-0.1252	0.0972	0.1976
TG 50:5	-0.1106	0.0836	0.1860	-0.0996	0.1436	0.4878	-0.1321	0.0986	0.1806
TG 50:6	-0.2335	0.0804	0.0037	-0.2594	0.1342	0.0533	-0.2286	0.096	0.0172
TG 51:1	-0.0481	0.0794	0.5451	-0.1067	0.1458	0.4643	-0.0306	0.0919	0.7392
TG 51:2	-0.0777	0.0827	0.3477	-0.1024	0.1321	0.4383	-0.0518	0.1031	0.6153
TG 51:3	-0.0264	0.0752	0.7256	-0.0309	0.137	0.8214	-0.0171	0.0876	0.8454
TG 51:4	-0.0588	0.0774	0.4472	-0.0524	0.1135	0.6444	-0.0761	0.1049	0.4685
TG 51:5	-0.1237	0.0796	0.1204	-0.0468	0.1259	0.7102	-0.2037	0.0995	0.0407
TG 52:0	-0.0992	0.0798	0.2142	-0.1478	0.1311	0.2595	-0.0769	0.098	0.4326
TG 52:1	-0.094	0.0793	0.2356	-0.1491	0.1309	0.2547	-0.0627	0.0975	0.5201
TG 52:2	-0.0802	0.0779	0.3032	-0.2108	0.1416	0.1366	-0.0241	0.0895	0.7880
TG 52:4	-0.0774	0.0771	0.3158	-0.1896	0.1458	0.1933	-0.0441	0.0873	0.6140
TG 52:5	-0.0944	0.081	0.2440	-0.076	0.1444	0.5986	-0.1262	0.0945	0.1819
TG 52:6	-0.2088	0.0827	0.0115	-0.1383	0.1325	0.2966	-0.2943	0.1003	0.0033
TG 53:0	0.0743	0.0757	0.3268	0.0415	0.1127	0.7130	0.09	0.1013	0.3741
TG 53:1	-0.0866	0.0767	0.2588	-0.081	0.1192	0.4964	-0.1014	0.0987	0.3042
TG 53:2	-0.0703	0.0796	0.3770	-0.0763	0.1363	0.5757	-0.0541	0.0955	0.5711
TG 53:3	-0.0812	0.0815	0.3190	-0.095	0.1332	0.4758	-0.0757	0.1004	0.4507
TG 53:4	0.0674	0.0785	0.3910	0.0754	0.1315	0.5664	0.0313	0.1004	0.7551
TG 53:5	-0.1369	0.0838	0.1026	-0.0197	0.1269	0.8766	-0.2737	0.1063	0.0100
TG 54:0	-0.0479	0.0806	0.5521	-0.0746	0.1153	0.5175	-0.0322	0.1129	0.7755

TG 54:1	-0.1108	0.0779	0.1551	-0.1021	0.0947	0.2811	-0.1353	0.1532	0.3769
TG 54:2	-0.1098	0.0787	0.1631	-0.1405	0.1181	0.2341	-0.0819	0.1052	0.4361
TG 54:3	-0.1085	0.0783	0.1660	-0.1715	0.1299	0.1865	-0.0737	0.0962	0.4433
TG 54:4	-0.1411	0.0801	0.0779	-0.1596	0.1309	0.2227	-0.1458	0.1003	0.1458
TG 54:5	-0.1469	0.0804	0.0677	-0.0853	0.1345	0.5256	-0.2206	0.098	0.0243
TG 54:6	-0.2063	0.0793	0.0092	-0.1038	0.1274	0.4154	-0.3362	0.0958	0.0005
TG 54:7	-0.3051	0.0758	0.0001	-0.2004	0.118	0.0895	-0.4396	0.0904	0.0000
TG 54:8	-0.2944	0.0741	0.0001	-0.2095	0.1078	0.0518	-0.4424	0.0963	0.0000
TG 55:1	0.0727	0.0789	0.3572	0.0114	0.1053	0.9135	0.2014	0.121	0.0959
TG 55:2	-0.084	0.0778	0.2803	-0.074	0.1072	0.4899	-0.0871	0.1164	0.4541
TG 55:3	-0.1187	0.0811	0.1431	-0.1153	0.1322	0.3832	-0.1221	0.1	0.2221
TG 56:0	-0.067	0.0753	0.3735	-0.06	0.085	0.4807	-0.0857	0.2082	0.6805
TG 56:1	-0.085	0.0751	0.2578	-0.077	0.0801	0.3366	-0.4922	0.7148	0.4911
TG 56:2	-0.0587	0.0759	0.4393	-0.0713	0.0842	0.3976	0.078	0.2332	0.7380
TG 56:3	-0.0911	0.0767	0.2349	-0.0977	0.1149	0.3954	-0.0864	0.1019	0.3966
TG 56:4	-0.1001	0.0802	0.2119	0.0598	0.1505	0.6910	-0.1922	0.0899	0.0325
TG 56:5	-0.1171	0.0831	0.1587	0.1279	0.1379	0.3537	-0.306	0.0944	0.0012
TG 56:6	-0.1544	0.0801	0.0537	0.0394	0.1284	0.7590	-0.351	0.0943	0.0002
TG 56:7	-0.2822	0.0731	0.0001	-0.1471	0.1133	0.1940	-0.4445	0.0856	0.0000
TG 56:8	-0.2901	0.072	0.0001	-0.1815	0.1028	0.0775	-0.4948	0.0932	0.0000
TG 56:9	-0.291	0.072	0.0001	-0.1944	0.111	0.0798	-0.4349	0.0862	0.0000
TG 56:10	-0.2812	0.072	0.0001	-0.2148	0.1009	0.0333	-0.4285	0.1001	0.0000
TG 58:1	-0.0689	0.075	0.3581	-0.0697	0.0798	0.3826	0.6542	0.8422	0.4373
TG 58:2	-0.0792	0.0749	0.2903	-0.0727	0.0797	0.3621	-0.6783	0.901	0.4515
TG 58:3	-0.089	0.075	0.2351	-0.0855	0.0805	0.2881	-0.1518	0.5083	0.7652
TG 58:4	-0.1061	0.0778	0.1729	-0.0728	0.1171	0.5340	-0.1616	0.1059	0.1269
TG 58:5	-0.1018	0.0788	0.1964	0.1502	0.1572	0.3392	-0.23	0.0843	0.0064
TG 58:6	-0.1847	0.0778	0.0176	-0.0145	0.1477	0.9220	-0.2941	0.0837	0.0004
TG 58:7	-0.2101	0.0741	0.0046	-0.0373	0.1254	0.7664	-0.3455	0.0831	0.0000
TG 58:8	-0.2632	0.0709	0.0002	-0.1304	0.118	0.2693	-0.3832	0.0787	0.0000
TG 58:9	-0.2668	0.0713	0.0002	-0.1545	0.1096	0.1587	-0.431	0.0848	0.0000
TG 58:10	-0.231	0.0726	0.0015	-0.1415	0.1075	0.1882	-0.4184	0.0941	0.0000
TG 58:11	-0.2589	0.072	0.0003	-0.2016	0.1019	0.0478	-0.4055	0.1007	0.0001
TG 60:10	-0.1528	0.0763	0.0452	0.0532	0.1177	0.6512	-0.3891	0.0885	0.0000
TG 60:11	-0.2148	0.0734	0.0034	-0.0627	0.1187	0.5973	-0.382	0.0837	0.0000
TG 60:12	-0.2872	0.0693	0.0000	-0.2001	0.1105	0.0702	-0.3964	0.0814	0.0000
TG 62:1	-0.0944	0.0752	0.2092	-0.0822	0.0807	0.3089	-0.6468	0.5411	0.2319
TG 62:12	-0.2027	0.0741	0.0062	-0.0468	0.1088	0.6668	-0.4071	0.0937	0.0000
TG 62:14	-0.2617	0.0711	0.0002	-0.2094	0.1054	0.0469	-0.3587	0.0934	0.0001
